# Tracking tau and cellular responses in human iPSC‐microglia: from uptake to seedable secretion, including in extracellular vesicles

**DOI:** 10.1002/alz.71337

**Published:** 2026-04-06

**Authors:** Maria Kreger Karabova, Anna del Ser‐Badia, Anne Hedegaard, Sam J. Washer, Zeynep Baykam, Darragh P. O'Brien, Iolanda Vendrell, Svenja S. Hester, Roman Fischer, Errin Johnson, Charlotte E. Melia, Teige R. S. Matthews‐Palmer, Rishi Matadeen, Alessia Santambrogio, Michael A. Metrick, Michele Vendruscolo, Sophie Keeling, Kimberly Ai Xian Cheam, William A. McEwan, Kenneth S. Kosik, Theresa A. Day, William S. James, Sally A. Cowley

**Affiliations:** ^1^ James and Lillian Martin Centre for Stem Cell Research, Sir William Dunn School of Pathology University of Oxford Oxford UK; ^2^ Target Discovery Institute, Centre for Medicines Discovery, Nuffield Department of Medicine University of Oxford Oxford UK; ^3^ Sir William Dunn School of Pathology University of Oxford Oxford UK; ^4^ Central Oxford Structural Molecular Imaging Centre, Kavli Institute for Nanoscience Discovery, Dorothy Crowfoot Hodgkin Building University of Oxford Oxford UK; ^5^ Current affiliation: Division of Structural Biology The Institute of Cancer Research (ICR) London UK; ^6^ Centre for Misfolding Diseases, Yusuf Hamied Department of Chemistry University of Cambridge Cambridge UK; ^7^ UK Dementia Research Institute at the University of Cambridge Cambridge UK; ^8^ Neuroscience Research Institute University of California Santa Barbara Santa Barbara California USA; ^9^ Department of Molecular, Cell and Developmental Biology University of California Santa Barbara Santa Barbara California USA; ^10^ Lilly Research Laboratories Eli Lilly and Company Indianapolis USA

**Keywords:** cryo‐electron microscopy, extracellular vesicle, induced pluripotent stem cells, lipoprotein receptor‐related protein 1, LRP1, microglia, phospho‐proteome, tau

## Abstract

**INTRODUCTION:**

Microglia have been implicated in the templated spread of tau aggregates in tauopathies through mouse studies. However, it is unclear whether these findings translate to human disease.

**METHODS:**

We challenged human induced pluripotent stem cell (iPSC)‐derived microglia‐like‐cells (iMGL) with monomeric and fibrillar recombinant tau and tau purified from Alzheimer's patient brains, examining in detail the uptake, processing, release, and seeding of tau by microglia.

**RESULTS:**

iMGL take up tau via lipoprotein receptor‐related protein 1 (LRP)1 and heparan sulfate proteoglycans, with leucine‐rich repeat kinase 2 affecting LRP1 trafficking. Monomeric tau is digested effectively with minimal effects on iMGL, but recombinant or brain‐derived tau fibrils induce chemokine/interferon response subtypes, alongside downregulation of homeostatic genes. Fibrillar tau is degradation‐resistant, can escape into the cytoplasm, and becomes phosphorylated on two specific residues. iMGL release partially digested fibrillar tau, including in extracellular vesicles, visualized by cryo‐electron microscopy, that seed aggregation in neurons.

**DISCUSSION:**

Our study reveals new insights into human microglial responses to tau, highlighting opportunities to limit pathogenic tau spread.

## Highlights

- Human iPSC‐microglia internalize tau via LRP1 and HSPG pathways.
- Fibrillar tau shifts microglia toward chemokine and interferon responses.
- Microglia phosphorylate internalized tau fibrils on specific residues.
- Cryo‐EM reveals intact tau fibrils packaged inside EVs.
- Tau secreted from microglia (including in EVs) is seed‐competent and induces neuronal tau aggregation.

## 1 INTRODUCTION

In neurodegenerative diseases known as tauopathies, the microtubule‐associated protein tau (MAPT) aggregates into insoluble deposits and spreads across connected brain regions in a spatiotemporal pattern that parallels disease progression.^1^ This stereotypical tau propagation was first observed in individuals with Alzheimer's disease (AD)^2^ and later described in other tauopathies, including progressive supranuclear palsy (PSP),^3^, ^4^ argyrophilic grain disease,^5^ and Pick's disease.^6^ Beyond cell‐autonomous mechanisms, tau deposits have been demonstrated to spread intercellularly in a prion‐like manner involving a templated aggregation of naïve tau by an internalized proteopathic tau seed. The prion‐like tau propagation was first demonstrated in a landmark study that injected P301S tau transgenic mice brain extracts into the brains of wild‐type human tau‐expressing mice.^7^ The findings have since been widely replicated using diverse tau seeds and in various in vivo^8^, ^9^, ^10^, ^11^ and in vitro^12^, ^13^, ^14^, ^15^ settings.

The prion‐like spread of tau pathology has been largely investigated in neurons, as tau is predominantly a neuronal protein. The results have established that fibrillar tau endocytosis^16^ and subsequent seeded tau aggregation in cultured neurons are mediated by the low‐density lipoprotein receptor LRP1^17^ and heparan sulfate proteoglycans (HSPGs)^18^, ^19^ and are highly dependent on the levels of membrane cholesterol.^20^ Tau aggregates can then be trafficked, including inside exosomes, to synaptically connected neurons,^21^ a process that is facilitated by neuronal activity, triggering seeded aggregation in neighboring neurons.^22^ Notably, high‐resolution cryo‐electron microscopy (cryo‐EM) and proteomic analyses have recently shown truncated hyperphosphorylated tau tethered inside extracellular vesicles (EVs) isolated from AD brains,^23^ though their precise cellular origin remains unclear, as pathological tau propagation may also involve other brain cell types as well as neurons.

Microglia are the major brain‐parenchyma resident phagocytes, maintaining brain homeostasis through constant surveillance and clearance of dead cells, redundant synapses, and protein aggregates. Dysregulated microglial function has been consistently associated with tauopathy risk and progression.^24^ *Post mortem* tissue analyses^25^ and positron emission tomography (PET) studies of living subjects^26^ have correlated proinflammatory microglial phenotypes with pathological tau deposition and cognitive decline. Importantly, genome‐wide association studies (GWAS) have identified certain AD risk variants for AD that are exclusively or predominantly expressed in microglia,^27^ further underscoring the necessity of deciphering the role of microglia in disease progression. However, most functional studies on the topic utilize mouse models or immortalized cell lines that poorly recapitulate human physiology.^28^, ^29^, ^30^, ^31^, ^32^, ^33^, ^34^, ^35^, ^36^, ^37^, ^38^ Many AD‐relevant gene variants, particularly those expressed in microglia, are not well represented in rodent models,^39^ so studying pathophysiological mechanisms in a human microglia model is relevant for generating translationally relevant information. Induced pluripotent stem cell (iPSC)‐derived microglia represent an accurate model for studying human microglia in vitro, being terminally differentiated and retaining the genetic background of the donors.

**FIGURE 1:**
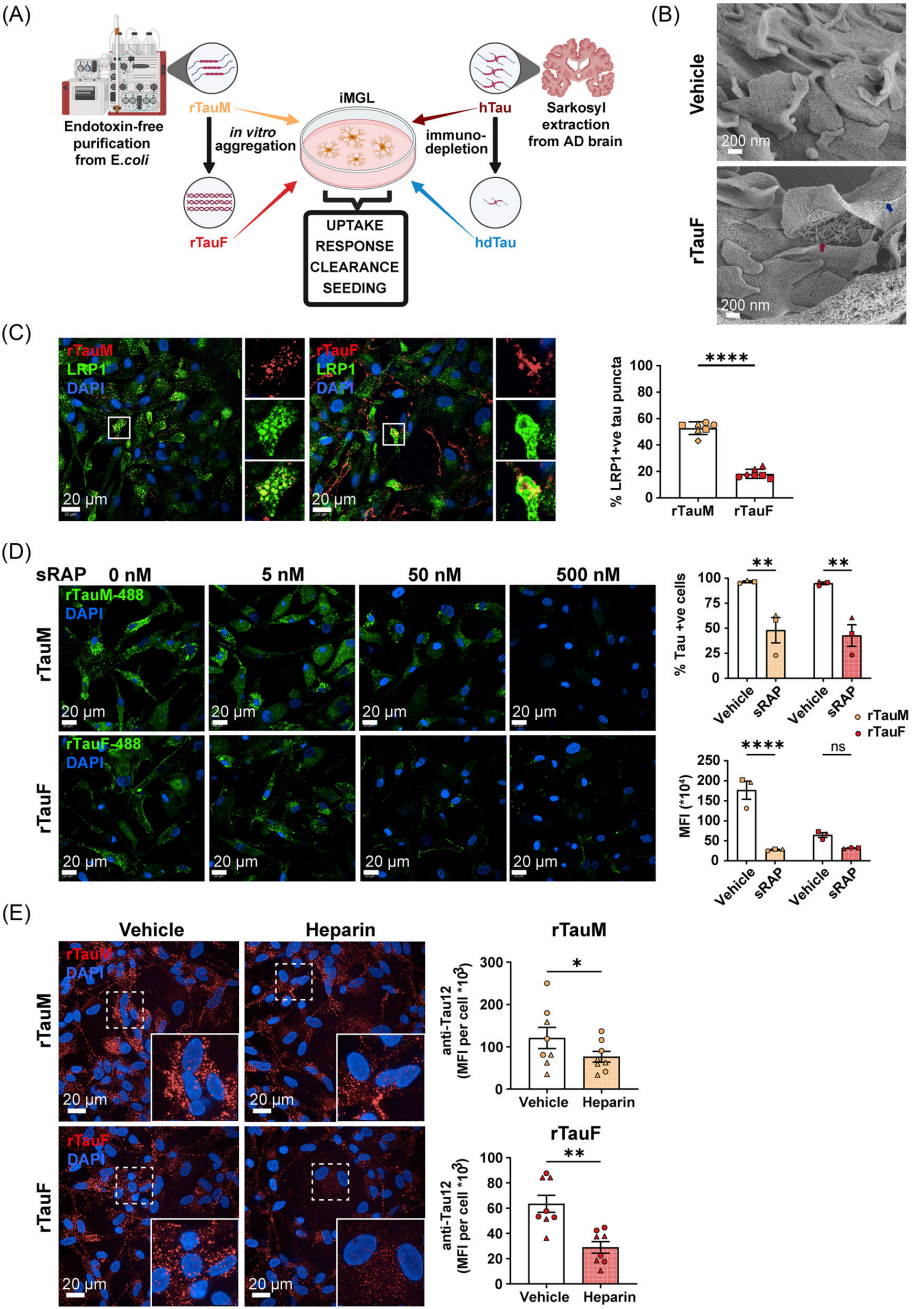
Monomeric and fibrillar tau internalization by iMGL is mediated by LRP1 and HSPGs. (A) Schematic overview of experimental setup. (B) Scanning electron micrographs depicting vehicle‐treated iMGL (top) or rTauF tethered to the iMGL plasma membrane (bottom, blue arrow) and during uptake (red arrow). Magnification = 40.48 K. (C) Representative confocal microscopy images of iMGL showing LRP1 co‐localizing with anti‐Tau12 immunostained tau monomer and fibrils after 2 h incubation. Quantification of (C) on the right, two‐way ANOVA with Šídák multiple comparison test, *n* = 1–2 in four control cell lines. (D) 2 h incubation of iMGL with sRAP and DyLight 488‐conjugated rTauM or rTauF reduces tau uptake in a dose‐dependent manner. On the right, Flow cytometry analysis of 2 h DyLight 488‐conjugated rTauM and rTauF uptake in the presence of 500 nM sRAP. Data are shown as mean ± SEM of *n* = 1 in three control cell lines. Two‐way ANOVA with Bonferroni's multiple comparison test was used; **p < 0.01, ****p < 0.0001. (E) Representative confocal microscopy images and quantification of Tau12 mean integrated density in iMGL incubated with 10 µg/mL heparin for 1 h, and challenged with 1 µg/mL rTauM or rTauF for 2 h. Data are represented as mean ± SEM of *n* = 4 in two control cell lines. One‐way ANOVA with Dunn's multiple comparison test; *p < 0.05, **p < 0.01. HSPG, heparan sulfate proteoglycans; iMGL, induced pluripotent stem cell (iPSC)‐derived microglia‐like cells; rTauM, recombinant tau monomer; rTauF recombinant tau fibrils; hTau, human Alzheimer's brain‐derived tau; hdTau, tau‐depleted hTau; LRP1, lipoprotein receptor‐related protein 1; sRAP, Stable Receptor‐Associated Protein; SEM, standard error of the mean.

Here, we use human iPSC‐derived microglia models (iPSC‐derived microglia‐like‐cells [iMGL] and related iPSC‐primitive‐macrophages [iMac]) to explore the molecular mechanisms underlying the processing and spread of tau by human microglia. As with all myeloid cells, microglia are known to be exquisitely sensitive to bacterial endotoxin (lipopolysaccharide [LPS]), which binds Toll‐like receptor 4 [TLR4] and the myeloid‐cell‐specific co‐receptor CD14. Therefore, reagents for microglial studies, especially *Escherichia coli*‐derived recombinant proteins, need to have negligible endotoxin levels (Table S1). We have therefore optimized the production of endotoxin‐free recombinant tau to avoid inadvertent TLR‐mediated inflammatory signaling by residual LPS. We use this, alongside human AD brain‐derived tau, to challenge iPSC‐microglia (Figure 1A). We show that tau internalization by human microglia is mediated primarily by HSPGs for fibrils and by LRP1 for monomeric species (with leucine‐rich repeat kinase 2 [LRRK2] influencing LRP1 trafficking). Human microglia cannot fully degrade fibrillar tau, and their transcriptome shifts toward chemokine and interferon response subtypes. Furthermore, we show that microglia undergo extensive changes to their phosphoproteome upon tau fibril uptake and can post‐translationally modify internalized recombinant tau fibrils at two specific residues. Microglia secrete undegraded seeding‐competent tau to the conditioned medium (CM), including in EVs, and tau fibrils are clearly visible by cryo‐EM as packaged inside these EVs, supporting the role of human microglia in tau spread in tauopathies.

## 2 METHODS

See Supplementary Materials for extended methods and reagent tables.

### 2.1 iPSC culture and ethics statement

Human iPSC lines from four healthy donors (one also gene‐edited to knockout LRRK2) and three Parkinson's disease (PD) patients harboring a LRRK2 G2019S allele were used. One healthy donor and one LRRK2 donor were heterozygous for ApoE ε4. See Table S2 for line details and Table S3 for which lines were used in which experiments. The human iPSC lines used in this study were previously derived from dermal fibroblasts from donors recruited as part of the Oxford Parkinson's Disease Centre/EU IMI program StemBANCC, using Cytotune Sendai reprogramming viruses (A16517 Thermo Fisher Scientific). SFC840‐03‐03, SFC841‐03‐01, SFC856‐03‐04, SFC832‐03‐06, and SFC855‐03‐06 were all published previously^40^, ^41^, ^42^, ^43^, ^44^ and are deposited in the European Bank for induced pluripotent Stem Cells (EBiSC) (STBCi026‐A, STBCi044‐A, STBCi063‐A). SFC833‐03‐05 is deposited in EBiSC (STBCi005‐B), and its characterization data are available on hPSCReg. SFC840‐03‐03 LRRK2−/− D10 was edited using CRISPR/Cas9 to knock out both alleles of LRRK2, was published previously,^43^ and is also deposited in EBiSC (STBCi026‐A‐1). KOLF2.1S was also generated using Cytotune as part of the Human Induced Pluripotent Stem Cells Initiative and was published previously.^45^ iPSC lines were curated in the James & Lillian Martin Centre for Stem Cell Research (Sir William Dunn School of Pathology, University of Oxford) as quality controlled frozen banked stocks and cultured in mTeSR1 (Catalog No. 85850, STEMCELL Technologies) or OXE8^46^ on Geltrex (Catalog No. A1413302, Invitrogen) at 37°C, 5% CO_2_ with minimal subsequent passaging (as clusters using 0.5 mM EDTA [#15575‐020, Invitrogen]) to maintain karyotypic integrity, as previously described.^42^

**RESEARCH IN CONTEXT**

1. **Systematic review**: The authors reviewed studies investigating the role of microglia in tauopathies using PubMed and recent conference proceedings. Prior work (conducted mostly in rodent models of the disease or immortalized cell lines) implicated microglia in tau uptake and release but lacked direct human evidence.
2. **Interpretation**: This study demonstrates that human iMGL internalize tau via LRP1 and HSPGs depending on tau conformation but fail to degrade internalized fibrillar tau, which they phosphorylate and secrete in seeding‐competent form, including packaged as fibrils within extracellular vesicles, as visualized by cryo‐EM. These findings establish mechanistic parallels with, but also human‐specific differences from, previous animal data, clarifying microglial contributions to tau propagation.
3. **Future directions**: Future studies should determine how genetic risk variants expressed in human microglia influence tau processing by microglia and assess mechanisms regulating tau release to therapeutically limit pathogenic tau spread in tauopathies.

Ethical approval for the derivation and use of iPSC lines with the prefix SFC was previously obtained with written informed consent by donors who specifically stated that their skin biopsies would be used for the derivation of pluripotent stem cell lines (Ethics Committee: National Health Service, Health Research Authority, NRES Committee South Central, Berkshire, UK, Research Ethics Committee [REC] 10/H0505/71). KOLF2.1S was obtained under a material transfer agreement from the Wellcome Sanger Institute, Cambridge, UK (REC Reference 09/H0304/77, covered under Human Materials and Data Management Committee 14/013 and REC reference 14/LO/0345). All experiments were performed in accordance with UK guidelines and regulations and as set out in the REC.

### 2.2 Differentiation of macrophage precursors and macrophages (iMac) from iPSCs

iPSCs were differentiated along a primitive myeloid route using our previously developed serum‐ and feeder‐free protocol.^47^ This differentiation pathway has been shown to be c‐myb‐independent,^48^ implying a primitive ontogeny that approximates the embryonic/fetal origins of primitive macrophages, including microglia.

Briefly, iPSCs were lifted with TryplE (Catalog No. 12604013, Gibco) and seeded into AggreWell 800 plates (Catalog No. 34815, STEMCELL Technologies) at 4 × 10^6^ cells per AggreWell in OXE8 medium (with 10 µM ROCK inhibitor Y‐27632, Catalog No. 1201029, Abcam, on day of seeding) supplemented with 50 ng/mL BMP4 (Catalog No. PHC9534, Peprotech), 50 ng/mL vascular endothelial growth factor (Catalog No. PHC9394, Peprotech), and 20 ng/mL SCF (Catalog No. 130‐096‐695, Miltenyi Biotec) to promote embryoid body (EB) formation directed to mesoderm and hemogenic endothelium. After daily feeding for 5 to 7 days, EBs were transferred to T175 flasks (“factories”) and cultured with 25 ng/mL IL‐3 (Catalog No. PHC0033, Invitrogen) and 100 ng/mL Macrophage Colony‐Stimulating Factor (M‐CSF)_ (Catalog No. PHC9501, Invitrogen) to promote primitive myeloid differentiation. Non‐adherent primitive macrophage precursors were harvested weekly between weeks 5 and 12 of the factory lifetime for final differentiation.

For final differentiation to primitive generic macrophages, precursors were cultured for 7 days in macrophage medium^46^ containing 100 ng/mL M‐CSF (Table S4) at a density of 8 × 10^4^/cm^2^ on standard tissue culture plates (Corning) or imaging plates (Perkin Elmer). Experiments with iMac were limited to those conducted early in the study before a new fully defined iPSC‐microglia protocol utilizing ITMG medium was established, which was used for all experiments except those marked in the supplementary Figures.

### 2.3 Differentiation of iMGL cells from macrophage precursors

iPSC‐microglia were differentiated from the aforementioned myeloid differentiation cultures as described by Washer et al.^49^ Briefly, harvested precursors were cultured for 14 days (incorporating 3 × 50% medium changes) in ITMG microglia medium (Table S4), containing 100 ng/mL IL‐34, 25 ng/mL M‐CSF, and 10 ng/mL Granulocyte‐Macrophage‐CSF to aid microglia survival and 50 ng/mL transforming growth factor beta 1 to promote microglia identity and maturation.

### 2.4 Recombinant tau production

Recombinant full‐length 2N4R tau (rTauM) expressed in *E. coli* was purified by sequential column chromatography that included an initial on‐column Triton X‐114 step^50^ to deplete endotoxin from the lysed material (Figure S1A) to less than 0.01 EU/mL (at 1 mg/mL tau concentration). See Supplementary Methods and Tables S5 and S6 for details.

### 2.5 In vitro synthetic tau fibril assembly

After confirmation of purified human 2N4R tau monomers by intact mass spectrometry (Figure S1B), aggregation was induced by heparin at a 4:1 tau:heparin ratio and 2 mM dithiothreitol (DTT) for 11 days at 37°C with orbital shaking at 250 rpm (Figure S1C). Tau fibril concentration was determined by Pierce BCA Protein Assay Kit – Reducing Agent Compatible due to the presence of β‐mercaptoethanol and DTT in the sample. The formation of fibrils was evaluated by electron microscopy and Thioflavin T assay (Figure S1,D,E), and seedability was assessed by 4R tau real‐time quaking‐induced conversion (RT‐QuIC) and cell‐based seeding assays (Figure S1F,G). Toxicity in iMGL was assessed with resazurin viability assay (Figure S2D).

### 2.6 Enrichment of tau PHFs from human AD brains

Brain‐derived tau (hTau) was enriched from Alzheimer's patient brains obtained from the London Neurodegenerative Diseases Brain Bank at King's College London and quality controlled by EM and Western blot (Figure S2A–C) and toxicity assessed (Figure S2D,E). A treatment dose of 0.25 µg/mL was chosen as this gave a final endotoxin level of 0.001 EU/mL in the culture medium and conferred no toxicity to iMGL after 24 h incubation. See Supplementary Methods for details.

### 2.7 Immunodepletion of hTau

Immunodepletion of tau from the hTau sample was performed using the Dynabeads Antibody Coupling Kit (Catalog No. 14311D, Life Technologies), following the manufacturer's protocol.

In short, two preparations of 5 mg of beads were washed and then mixed with either 50 µg of mouse IgG1 antibody (control depletion, Catalog No. SC‐3877, Santa Cruz) or 50 µg of HT7 antibody (tau depletion,Catalog No. MN1000, Invitrogen), achieving a coupling of 10 µg antibody/mg of beads. The mixtures were incubated on a roller overnight at 37°C. The next day, antibody‐coupled beads were washed sequentially in 800 µL of HB and LB buffers, supplemented with 0.1% Tween 20 (Catalog No. P7949, Sigma–Aldrich). Beads then underwent two quick washes in SB buffer, followed by a 15 min wash while on a roller at RT and final storage in SB buffer at 25 mg/mL. For depletion, 5 mg of beads (control and tau‐antibody coupled) were each mixed with 1 µg of hTau and left on a rotating wheel overnight at 4°C. Beads were removed from immuno‐depleted solutions using a magnet and retained to run on a gel as a control.

### 2.8 Tau, pharmacological, and LentiCRISPR treatments of iMac/iMGL

Unless otherwise indicated, iMac/iMGL were incubated with 1 µg/mL human recombinant 2N4R tau monomer (rTauM) or heparin‐aggregated fibrils (rTauF) or 0.25 µg/mL human AD brain‐derived tau (hTau). A 2.5% TryplE incubation for 1 min followed by 1× PBS wash was used to remove uninternalized soluble tau based on previously published protocol. For tau uptake experiments, cells were preincubated with 500 nM Stable Receptor‐Associated Protein (sRAP)^51^ (Catalog No. 153996, Ximbio), 10 µg/mL heparin (Catalog No. H3393, Sigma‐Aldrich), or 5 ng/mL FKN (Catalog No. 300‐31, PeproTech) for 1 h before tau treatment (Table S7). For tau degradation experiments, cells were incubated with a cocktail of protease inhibitors (50 µM Leupeptin, 50 µM Pepstatin A, and 50 µM E64d) or 25 µM MG‐132 for 24 h. All incubation times are indicated in the figure legend.

Three LRP1 CRISPR/Cas9 lentiviral vectors were prepared to knock down LRP1. The guide RNA (gRNA) sequences targeted exons 12, 13, and 38 of LRP1 (Table S4). In addition, eight intergenic control (INTG) CRISPR/Cas9 lentivectors, predicted not to affect LRP1 or other protein‐coding sequences, were prepared as a negative control of DNA damage response. Golden Gate Assembly via *BsmB*I was used to clone gRNA oligos into pLentiCRISPRv3 backbone, a novel version of pLentiCRISPRv2 (Addgene, Catalog No. 52961) with a modified gRNA scaffold.^52^ Plasmids were transformed into chemically competent Stbl3 *E*. *coli* (Thermo Fisher Scientific, C737303) for amplification and plasmid purification. Lentiviral production was carried out using HEK293T, JetPRIME transfection reagent (Polyplus, 101000046), 5.5 µg of psPAX2 lentiviral packaging plasmid DNA, 4.4 µg of pMD2.G lentiviral envelope plasmid DNA, and 9 µg of pooled LRP1 gRNA plasmids or pooled INTG gRNA plasmids. Virus was collected at 48 and 72 h and concentrated by ultracentrifugation. Virus transduction was carried out during plating of myeloid precursors, in differentiation medium supplemented with 4 ug/mL of polybrene (Sigma, TR‐1003‐G), together with 1:400 dilution of Vpx packaged in virus‐like‐particles (VLPs) (pSIV3+_Vpx plasmid, Cosset lab, Inserm, France). The human immunodeficiency virus type 2 (HIV‐2)‐ and simian immunodeficiency virus (SIV)‐encoded accessory protein Vpx counteracts the cytoplasmic nucleotide depletion by SAM Domain and HD domain‐containing protein 1 (SAMHD1), thereby improving lentiviral transduction efficiency in SAMHD1‐expressing myeloid cells such as macrophages and microglia.^53^

### 2.9 Immunocytochemistry and flow cytometry

Cells were fixed with 2% Paraformaldehyde (PFA) for 10 min at room temperature (RT), washed with PBS, and permeabilized with 0.1% Triton‐X100 for 10 min at RT. Cells were incubated with blocking buffer (10% donkey serum, 5% BSA, and 0.01% sodium azide in PBS) for 1 h at RT and incubated overnight at 4°C with primary antibodies diluted in blocking buffer (Tables S8 and S9). After PBS washes, cells were incubated with secondary antibodies diluted in blocking buffer for 1 h at RT and with DAPI diluted in PBS for 10 min at RT (Table S8). Cells were washed with PBS and imaged using the automated spinning disk confocal Opera Phenix High Content Screening System (Perkin Elmer). Image analysis was performed with Columbus Image Data Storage and Analysis System (CambridgeSoft).

### 2.10 Transmission electron microscopy (TEM)

The formation of in vitro aggregated recombinant tau fibrils was assessed by negative stain TEM. Tau monomer and fibril preparation were diluted 1:20 in 25 mM HEPES (Catalog No. H0887, Sigma‐Aldrich), pH 7.1. 10 µL of each sample was applied to freshly glow‐discharged (Pelco EasiGlow) 300‐mesh carbon‐coated copper grids (Catalog No. C267, TAAB Laboratories) for 2 min. After removing excess sample with filter paper, grids were washed once with ddH_2_O, negative‐stained with 2% uranyl acetate (Catalog No. R1260A, Agar Scientific) for 20 s, and air‐dried.

TEM was also used to assess the uptake of tau fibrils by iMac. Cells were differentiated on glass coverslips inserted into a 24‐well tissue culture plate (Catalog No. 3524, Costar). Following overnight incubation with vehicle or 1 µg/mL of rTauF, cells were fixed with pre‐warmed 2.5% glutaraldehyde (Catalog No. R1020, Agar Scientific) and 2% PFA in 0.1 M PIPES buffer (Catalog No. P6757, Sigma‐Aldrich) pH 7.2, for 1 h at RT. After five washes with 0.1 M PIPES buffer, cells were incubated with 50 mM glycine (Catalog No. G7126, Sigma‐Aldrich) in 0.1 M PIPES buffer for 15 min at RT, then washed again with 0.1 M PIPES buffer. Secondary fixation was carried out with 1% osmium tetroxide (Catalog No. O001/1, TAAB Laboratories) + 1.5% potassium ferrocyanide (Catalog No. 223111000, Acros Organics) in 0.1 M PIPES buffer at 4°C for 1 h. Samples were then washed five times with ddH_2_O, stained with 0.5% uranyl acetate (Catalog No. R1260A, Agar Scientific) overnight at 4°C in the dark, then washed again twice with ddH_2_O, 10 min each time while being protected from light. Consecutive, 10‐min‐long, ice‐cold ethanol incubations (30%, 50%, 70%, 80%, 90%, and 95%) on ice were used to dehydrate the samples. A final 20‐min incubation in 100% dry ethanol was repeated twice. After epoxy resin infiltration (Catalog No. AGR1140, Agar 100‐Hard epoxy resin, Agar Scientific), coverslips were removed from the wells, inverted onto BEEM capsules (Catalog No. AGG360‐1, Type 00, Agar Scientific), filled with fresh 100% resin, and blocks were polymerized for 24 h at 60°C. Ultrathin (90 nm) sections of the resin‐embedded cells were obtained with a Diatome diamond knife on a Leica UC7 ultramicrotome. Individual sections were mounted onto 200‐mesh carbon‐coated copper grids (Catalog No. C101, TAAB Laboratories), stained with Reynold's lead citrate (Catalog No. 1963, Reynolds) for 5 min at RT, washed with five droplets of degassed ddH_2_O, and air‐dried. All images were acquired using a Gatan OneView camera on a FEI Tecnai T12 transmission electron microscope operated at 120 kV.

### 2.11 Scanning electron microscopy (SEM)

SEM was used to visualize iMac/iMGL following rTauF incubation. As with the TEM protocol, cells were differentiated on glass coverslips inserted into 24‐well tissue culture plates (Catalog No. 3524, Costar), then incubated overnight with vehicle or 1 µg/mL of tau fibrils. Primary fixative of 2.5% glutaraldehyde in 0.1 M PIPES buffer, pH 7.2, was added for 1 h at RT for cross‐linking. Cells were rinsed three times with 0.1 M PIPES buffer, then fixed with 1% osmium tetroxide in 0.1 M PIPES buffer for 1 h at 4°C. After three washes with ddH_2_O, samples were dehydrated in increasingly more concentrated ethanol (50% to 70% to 90% to 95%) for 5 min each, then three times in 100% ethanol for 10 min each. Cells were then chemically dried using hexamethyldisilazane (HMDS, Catalog No. 440191, Sigma‐Aldrich) as follows: 1:1 100% ethanol:HMDS for 3 min, followed by two consecutive 2‐min incubations with pure HMDS, after which the HMDS was removed, and the samples were left to dry overnight. Samples were sputter coated with ∼15 nm of gold using a Quorum Technologies Q150R ES coating unit. Images were captured with a Zeiss Sigma‐Aldrich 300 Field Emission Gun Scanning Electron Microscope (FEG‐SEM) operated at an accelerating voltage of 2 kV.

### 2.12 Correlative light and electron microscopy (CLEM)

iMGL, previously fed with 488‐labeled rTauF, were fixed in 4% formaldehyde and 2.5% glutaraldehyde in 0.1 M PIPES buffer pH 7.2 for 1 h at room temperature. Cells were then stained and processed into resin as described in the earlier TEM section. The resulting sample block was trimmed to the grid co‐ordinate noted during light microscopy imaging that contained cells of interest. Sections of 200 nm were cut from the resin blocks using a Leica UC7 Ultramicrotome and collected onto formvar‐coated 3 mm copper slot grids (Agar). The sections were then post‐stained with lead citrate and imaged using a JEOL JEM‐1400Flash 120 kV TEM equipped with a Gatan Rio camera or JEOL JEM‐2100Plus 200 kV TEM equipped with a Gatan OneView camera. Tilt series were collected at 200 kV over 116° to 120° around the regions of interest at 1° increments using SerialEM (4.0.26).

TEM images of the regions of interest were overlaid with the corresponding confocal *z*‐plane using linear transformations in Adobe Photoshop 2020. Tilt series alignment and tomogram reconstruction by weighted back‐projection were carried out in IMOD (4.11.25), and the resulting tomograms were binned (bin2) and filtered (gaussian filter sigma 1.5) in ImageJ (Fiji 1.54f). Images stacks were segmented using Microscopy Image Browser (version 2.91) and visualized with 3D Slicer (5.8.0 r33216).

### 2.13 Protein quantification

#### 2.13.1 Protein sample collection

For total protein extraction from cells, cultures were washed once with ice‐cold 1× PBS, then lysed directly with appropriate amounts of ice‐cold radioimmunoprecipitation assay (RIPA) buffer (Catalog No. 89901, Thermo Fisher Scientific) supplemented with cOmplete ULTRA Tablets, Mini, EDTA‐free, EASYpack Protease Inhibitor Cocktail (Catalog No. 5892791001, Roche) and Pierce Phosphatase Inhibitor Mini Tablets (Catalog No. A32957, Thermo Fisher Scientific). Cell lysates were scraped into LoBind tubes (Catalog No. 15178344, Eppendorf) and centrifuged at 21,000 × g for 30 min at 4°C. Cleared lysates were snap‐frozen in liquid nitrogen and stored at −80°C for further use.

#### 2.13.2 Cell‐surface biotinylation

Cell surface proteins were extracted using Pierce Cell Surface Biotinylation and Isolation Kit (Catalog No. A44390, Thermo Fisher Scientific). The manufacturer's protocol was adapted as follows. Adherent cells were washed once with BupH PBS at RT and biotinylated with 2 mL of 1× EZ‐Link Sulfo‐NHS‐SS‐Biotin for 10 min at RT. After two washes with ice‐cold BupH Tris Buffered Saline (TBS), cells were scraped into 1 mL of TBS, transferred to Eppendorfs, and centrifuged at 500 × g for 5 min at 4°C. Supernatants were discarded. Cell pellets were lysed on ice for 30 min with 200 µL of kit‐provided lysis buffer, supplemented with Halt Protease and Phosphatase Inhibitor Cocktail, EDTA‐free (100×) (Catalog No. 78441, Thermo Fisher Scientific), then centrifuged at 21,000 × g for 5 min at 4°C. Biotinylated proteins in the clarified supernatant were captured by 1 h RT incubation with NeutrAvidin Agarose in 1:1 ratio. The captured protein–resin complex was washed four times before final protein elution with 50 µL of kit‐provided elution buffer, supplemented with 10 mM DTT (Catalog No. 43816, Sigma‐Aldrich). Eluted protein samples were snap‐frozen in liquid nitrogen and stored at −80°C until required.

#### 2.13.3 Sample quantification

Protein concentration in all cell lysate and EV samples was determined with Pierce BCA Protein Assay Kit (Catalog No. 23227, Thermo Fisher Scientific), diluting the sample in appropriate buffer where necessary to remain within the standard curve.

To assess the total protein content of purified EVs, samples were lysed using 10× RIPA buffer (Catalog No. 9806, Cell Signaling) due to the small volumes (<100 µL). 10× RIPA was diluted to 1× by adding appropriate amounts to quantified volumes of EV suspensions after which samples were briefly vortexed and incubated for 30 min on ice. EV samples were lysed immediately prior to experimentation.

#### 2.13.4 Western blot

Levels of total and cell‐surface proteins of interest in cell lysates, purified EVs and tau samples were analyzed with Western blot. Samples were mixed with NuPAGE 4× LDS sample buffer (Catalog No. NP0007, Invitrogen) and NuPAGE 10× Sample Reducing Agent (Catalog No. NP0009, Invitrogen), unless eluted in buffer supplemented with DTT (Catalog No. 43816, Sigma‐Aldrich). Samples were then heated at 70°C for 10 min. A total of 25 µL of sample containing a minimum of 10 to 50 µg of protein was loaded onto Novex WedgeWell 8% to 16%, Tris‐Glycine pre‐cast mini gels (Catalog No. XP08165BOX, Invitrogen), along with 5 µL of Precision Plus Protein Dual Color Standards (Catalog No. 161‐0374, Bio‐Rad), and run in Novex Tris‐Glycine SDS Running Buffer (Catalog No. LC2675, Invitrogen) at 180 V for ∼45 to 60 min.

Following the electrophoresis, gels were rinsed with ddH_2_O and 1× Trans‐Blot Turbo Transfer Buffer (Catalog No. 10026938, Bio‐Rad) before semi‐dry protein transfer to a low‐fluorescence PVDF transfer membrane (#22860, Thermo Fisher Scientific), using the Mixed Molecular Weight (1.3 A, 25 V, 7 min) pre‐programmed settings on a Trans‐Blot Turbo transfer machine (Bio‐Rad). Membranes were blocked with iBind Flex blocking solution (Catalog No. SLF1020, Thermo Fisher Scientific) for 1 h at RT and probed with relevant primary antibodies, diluted in iBind Flex (Table S9), overnight at 4°C. After three washes with 1× PBS supplemented with 0.1% Tween 20 (PBS‐T, Catalog No. P7949, Sigma‐Aldrich) membranes were further stained with relevant, LI‐COR fluorophore‐conjugated secondary antibodies (Table S8) diluted in iBind Flex supplemented with 1:200 10% SDS (Catalog No. 75746, Sigma‐Aldrich). Unbound antibody was removed with six 1× PBS‐T washes. Blots were visualized using the Odyssey Sa Infrared Imaging System (LI‐COR Biosciences). Image Studio Lite open‐source software version 5.2 was used for densitometric analysis.

### 2.14 Enzyme‐linked immunosorbent assay (ELISA)

Levels of intracellular tau and tau released to CM were quantified using Invitrogen Tau (Total) Human ELISA kit (Catalog No. KHB0041, Thermo Fisher Scientific). Cytokine secretion was quantified using Human Uncoated IL‐1β and IL‐6 ELISA Kits (Catalog No. 88‐7261‐88 and 88‐7066‐88, Thermo Fisher Scientific). Protocols were followed as per the manufacturer's instructions. Total protein was extracted and quantified from cells following vehicle or tau treatment, as described earlier. CM for tau or cytokine quantification was centrifuged at 400 × g for 5 min at 4°C to remove any floating cells and cell debris, aliquoted to 96‐well V‐bottom plates, parafilmed, and stored at −80°C until required.

After thawing, sample dilution was optimized to 1:100 for tau quantification in cell lysates, 1:10 for tau quantification in supernatants, and 1:10 and 1:100 for cytokine quantification in supernatants. Absorbance (450 nm) was measured using a Spectramax M5e Multi‐mode Microplate reader (Molecular Devices). Background reading was subtracted from all values. Tau or cytokine concentration in samples was calculated by interpolation from standard curve (sigmoidal, 4PL, X is concentration) in GraphPad Prism version 9.0. Values above and below the range of the standard curve were excluded. If multiple dilutions of a sample were assessed, values presented are an average of the dilutions.

### 2.15 EV purification

To enhance the release of EVs, iMGL cells were treated with 5 mM ATP for 15 min, added directly to the culture medium. The CM was then collected and cell lysates processed as described in the “Protein quantification” section. The CM underwent a series of differential centrifugation steps following Thery et al^54^, with minor alterations to purify mixed‐size EV populations.

In short, the first centrifugation at 500 × g for 5 min pellets any floating cells, the second at 2000 × g for 20 min pellets dead cells and the third at 10,000 × g for 30 min pellets cell debris. At centrifugation Steps 1–3, the supernatant was retained and the pellet discarded, and to finally pellet the EVs, the CM was ultracentrifuged at 100,000 × g for 90 min in either re‐usable 3.5‐mL polycarbonate tubes (Catalog No. 349622, Beckman Coulter) or single‐use 13.2‐mL ultra‐clear polypropylene tubes (Catalog No. 344059, Beckman Coulter), depending on the starting volume. Small volumes were processed in a Beckman Optima MAX‐XP tabletop centrifuge using a TLA 100.3 fixed‐angle rotor and larger volumes in a Beckman Optima XPN‐80 using a SW41Ti swinging bucket rotor. Samples were often frozen at −70°C after the second spin, and the protocol continued at a later date. After the last ultracentrifugation step, the supernatant was poured off and the tubes kept upside down for ∼1 min to let leftover supernatant run off and concentrate the EVs. For experiments where high concentrations of EVs were required (see section “Cryo‐electron tomography”), the pellet was re‐suspended without further addition of PBS; otherwise, the EV‐containing pellet was re‐suspended in 50 to 100 µL of PBS. To enhance re‐suspension, care was taken to wash around the bottom of the tube during re‐suspension, and tubes were kept on a rocker overnight at 4°C before being transferred to protein LoBind tubes (Catalog No. 10316752, Eppendorf) and stored at −70°C until further experimentation.

### 2.16 Nanoparticle tracking analysis (NTA)

The size distribution of EVs was measured using a NanoSight LM10 (Malvern Instruments) equipped with a 405‐nm laser.

Samples of purified EVs were diluted in filtered PBS to achieve an appropriate concentration for NTA; 1:500 gave a particle concentration of ∼10^9^ particles/mL. Approximately 1 mL of the diluted suspension was injected into the sample chamber via a syringe, from which it was introduced into the flow cell. Particle tracking commenced with the following settings: infusion rate = 50, temperature = 26°C, frame rate = 25 FPS, slider shutter = 1300, slider gain = 512. Each measurement was captured over a 60‐s duration and repeated five times prior to averaging. The size distribution was calculated using the Stokes‐Einstein equation, which relates the diffusion coefficient to particle size.

### 2.17 Viability assays

#### 2.17.1 Resazurin assay

Resazurin (Catalog No. 199303‐5G, Sigma‐Aldrich) was used to determine the cytotoxicity effect of drugs, rTauM and rTauF on iMac/iMGL. At the end of the pharmacological or tau treatment, cells were incubated with 10 µg/mL resazurin for 2 h at 37°C, 5% CO_2_. Viable cells with intact mitochondria reduced the non‐fluorescent blue resazurin to red fluorescent resorufin (Ex: 530 to 560 nm, Em: 590 nm), which was detected on the SpectraMax M5 microplate reader using the SoftMaxPro software version 5. The amount of resorufin produced was proportional to the number of viable cells.

#### 2.17.2 LDH assay

The cytotoxicity of the human AD brain tau seed preparation was assessed using a lactate dehydrogenase (LDH) detection kit (Catalog No. 426401, Biolegend), following the manual for “Assay using cell supernatant.” In short, macrophage precursors were plated at 32,000 cells/well in 96‐well plates and differentiated to iMGL. At DIV13, iMGL cells received a full medium change in preparation for hTau treatment to ensure equal volume in each well. The hTau seeds were pre‐mixed in 10 µL/well of iMGL media at 10× final concentrations to achieve 0.1, 0.25, 0.5, 0.75 a 1 µg/mL in test wells and added directly to the existing iMGL medium in each well to avoid tau sticking to the plastic. When handling the hTau preparation, LoBind plasticware was used throughout. At 23.5 h after treatment, wells designated for the “max LDH release” control condition were exposed to 10% triton‐x to lyse the cells and incubated at 37°C for 30 min until harvesting of the supernatant at 24 h. Vehicle‐control wells contained untreated iMGL and background control wells contained iMGL medium. Supernatants were centrifuged at 500 × g for 5 min in a V‐bottom plate to pellet cells and debris, then moved to a 96‐well plate for immediate assaying, following the kit instructions. Absorbance was read at 490 nm on a Spectramax M5e Multi‐mode Microplate reader (Molecular Devices) and the averaged background subtracted from all values.

### 2.18 RNA sequencing (RNA‐seq) analysis

Raw sequencing reads were trimmed of adapter sequences using Trim‐Galore before quality control (QC) with multiQC.^55^ Transcripts were quantified using Kallisto (version 0.44.0) with default options, with the additional “*bootstrapping*” command set to 50.^56^ The paired‐end sequencing reads for each sample were mapped to the human reference transcriptome (GRCh38.p14), which combined all cDNA sequences. The transcript abundances were imported and summarized to gene‐level counts using R library “tximport.”^57^

Data were prefiltered to include only genes with counts >10 in three or more samples. This reduced the dataset to 15,132 genes for the recombinant analysis, 15,090 for the brain tau analysis. Differentially expressed genes (DEGs) between the vehicle versus rTauM, vehicle versus rTauF, and hdTau versus hTau was performed using DESeq2 (version 1.42.1) using the Wald test, log_2_FC shrinkage was performed using the “apeglm” method. A gene was considered a DEG at false discovery rate (FDR) < 0.05 with a log_2_FC > 0.5 (upregulated) or < −0.5 (downregulated).^58^ GO analysis was performed using GOSeq (version 1.54.0).^59^ Gene lists for subtyping iMGL were taken from single‐cell Xenotransplanted samples from ref. ^60^ The list of AD GWAS hits was extracted from Bellenguez et al.^27^

Code is deposited in GitHub: https://github.com/S‐Washer/Karabova_2025_Tracking_tau_and_cellular_responses_in_microglia_RNAseq

### 2.19 Flow cytometry

Cells differentiated on 24‐well tissue culture plates (Catalog No. 3524, Corning) and treated according to experimental design were lifted by 10‐min incubation with StemProTM AccutaseTM (Catalog No. A1110501, Gibco) at 37°C, 5% CO_2_. Lifted cells in suspension were transferred to a 96‐well V‐bottom plate (Catalog No. 611V96 and 642000, Thermo Fisher Scientific) and kept at 4°C or on ice throughout all steps, unless stated otherwise. All centrifugation was carried out at 400 × g for 5 min.

For live‐cell flow cytometry analyses of phagolysosomal proteolysis and tau internalization, cells were spun, washed with 1× PBS, spun again, and resuspended in 40 µL/well of ice‐cold Invitrogen Live Cell Imaging Solution (LCIS) (Catalog No. A14291DJ, Thermo Fisher Scientific) in preparation for acquisition.

For cell surface marker staining, cells were lifted into ice‐cold 1× PBS, counted, spun, and resuspended in ice‐cold fluorescence‐activated cell sorting (FACS) buffer (1× PBS supplemented with 1% FBS [Catalog No. F9665, Sigma‐Aldrich], 10 µg/mL human‐IgG [Catalog No. I8640‐100MG, Sigma‐Aldrich], and 0.01% sodium azide) before being transferred into a 96‐well V‐bottom plate at 100,000 cells/well. Non‐specific Fc‐receptor interactions were blocked by 30‐min incubation in FACS buffer. Cells were spun again and stained with primary antibodies diluted in FACS buffer (Table S8) directly without fixation for 1 h in the dark. If fluorescent primary antibodies were used, isotype controls conjugated to the same fluorophore, obtained from the same company, were used at the same final antibody concentration. If non‐fluorescent primary antibodies were used, isotype control was achieved by staining with secondary antibody only.

Whenever secondary antibody incubation was necessary, cells were washed once with 1× FACS buffer following primary antibody incubation, then stained with secondary antibodies diluted in FACS buffer (Table S8) for 30 min in the dark. After staining, cells were washed once with FACS buffer and fixed by resuspension in 2% PFA (Catalog No. J61899.AP, Thermo Fisher Scientific) in FACS buffer. The protocol steps for total marker staining were identical to those used for surface marker staining, with the following exception: Cell fixation was carried out with 2% PFA in FACS buffer for 10 min at RT immediately after transfer to a 96‐well V‐bottom plate. Cells were then washed once with FACS buffer and permeabilized with 0.1% Triton‐X100 (Catalog No. T8787, Sigma–Aldrich) in 1× PBS for 10 min at RT, before blocking and staining.

Forward scatter (FSC‐H), side scatter (SSC‐A), and fluorescence measurements were obtained by passing single‐cell suspension through a Cytoflex LX (Beckman Coulter) flow cytometer. A minimum of 20,000 events were recorded per condition. Laser settings and gating strategies are described in detail in individual results sections. Acquired data were analyzed using the FlowJo version 10 software.

### 2.20 Proteomics

See Supplementary Methods for details of Sample preparation, proteomic digestion, phosphopeptide enrichment, manual tip enrichment for purified tau inputs, LC‐MS/MS of total proteome and phosphoproteome samples, EV and CM samples, and data analysis.

### 2.21 Cryo‐electron tomography (Cryo‐ET)

EVs were purified as described earlier from CM harvested from 10‐cm dish cultures of iMGL cells, treated with either vehicle or 1 µM rTauF overnight. Extra care was taken to reduce the final sample volume to ∼10 µL to concentrate the EVs on the grids. 2.5 µL of EV sample was applied to glow‐discharged lacey‐carbon grids (Catalog No. AGS187‐4, Agar scientific) three times using a Vitrobot (Mark IV, Thermo Fisher Scientific) using a blotforce of 6 for 3 s before plunge‐freezing in liquid ethane. No fiducials were added to avoid diluting the samples. The grids were then stored in liquid nitrogen until use.

#### 2.21.1 Microscope setup

Data were collected using a Thermo Fisher Scientific 300 kV Titan Krios electron microscope with a Gatan K3 Bioquantum direct electron detector. The pixel size was1.4 Å and the energy filter slit width 20 eV. Tilt series from specified areas of interest were acquired using a dose‐symmetric scheme with a tilt range of ± 51° at 3° increments, making the total electron dose 3 e^−^/area^2^ * 35 tilts = 105 e^−^/area^2^. The defocus range was set between −2.5 and −5.5 µm to optimize contrast.

#### 2.21.2 Tomogram reconstruction

The fiducial‐free tilt series were aligned using patch tracking in Aretomo version 1 from the IMOD software package using the simultaneous iterative reconstructive technique (SIRT). Tomograms were binned at either 4 or 8, making the reconstructed tomogram pixel sizes 5.6 Å (bin4) or 11.2 Å (bin8), and a lowpass filter of 0.035 (cut‐off radius), 0.35 (sigma) was applied via the “mtffilter” function in IMOD.

#### 2.21.3 Tomogram analysis and measurements

Tomograms were viewed and analyzed using 3dmod from the IMOD software package. For generating Z‐projections, tomograms with binning setting 8 were opened and rotated in the Slicer window and appropriate Z‐projections made, typically of ∼5 slices, so ∼11 nm in thickness. EV diameters were measured manually using the drawing tool “Measure” and panning through each tomogram to find the maximum diameter of each EV within the field of view. Fibrils were drawn as open contours through the 3D volume of EVs while tomograms were open in the slicer window with the image thickness set to 5 to enhance contrast. Measurements were made from EVs containing obvious fibrils, and within such an EV, only the fibrils with clear start and endpoints were measured, so it is not claimed that these quantifications capture the entire population and probably provide an underestimate in terms of EVs containing fibrils. All fibril measurements were expressed as absolute length (in comparison with length of uninternalized rTauF), as well as matched up with the diameter of the EV from which the fibril originated and then expressed both as a function of EV diameter and as a percentage of EV diameter. Similarly, for fibril crossover distances, in a subset of EVs where this phenomenon was very obvious, the intervals between nodes were drawn as open contours and averaged per fibril.

### 2.22 Thioflavin T (ThT)assay

ThT assay was used to verify the presence of β‐sheets in tau fibril preparations. 100‐µL reactions were prepared in black, clear‐bottom 96‐well PhenoPlate (Catalog No. 6055300, PerkinElmer) by mixing 28 µM tau monomer, 7 µM heparin, and 25 µM ThT (Catalog No. T3516‐5G, Sigma) in 1× PBS, pH 7.2. The sealed plate was incubated at 37°C with 250 rpm agitation for 11 days. ThT fluorescence readings (excitation wavelength 440 nm/emission wavelength 510 nm) were taken at regular intervals using a Spectramax M5e Multi‐mode Microplate reader (Molecular Devices). Background fluorescence of 1× PBS buffer was subtracted from all values at corresponding time points.

### 2.23 Tau RT‐QuIC assay

A modified version of the 4R tau RT‐QuIC seed amplification assay^61^, ^62^ was used to determine the seeding capacity of soluble and insoluble intracellular tau and tau secreted to CM by iMGL.

Cells were differentiated in six‐well tissue culture plates and treated overnight with 1 µg/mL of rTauM, rTauF, hTau, or vehicle. To remove uninternalized tau cells were incubated with 2.5% TryplE for 1 min^63^ followed by 1× PBS wash. Cells were left to process internalized tau in standard, tau‐free, iMGL differentiation medium at 37°C, 5% CO_2_. After 24 h, CM and cell lysates were collected. The CM was spun at 400 × g for 5 min at 4°C to remove floating cells and at 2000 × g for 20 min to remove cell debris, before being frozen on dry ice and stored at −80°C until further processing. Cells were lysed with 100 µL of ice‐cold Triton lysis buffer (1% Triton‐X100 in 50 mM Tris [Catalog No. T1503‐500G, Sigma‐Aldrich], 150 mM NaCl, pH 7.6) supplemented with EASYpack Protease Inhibitor Cocktail (Catalog No. 5892791001, Roche) and Pierce Phosphatase Inhibitor Mini Tablets (Catalog No. A32957, Thermo Fisher Scientific). Cell lysates were collected by scraping, then centrifuged at 21,000 × g for 30 min at 4°C. Supernatants containing total cell protein including soluble tau (the Triton fraction) were transferred to Eppendorfs. Pellets containing insoluble tau were solubilized in SDS lysis buffer (1% SDS in 50 mM Tris, 150 mM NaCl, pH 7.6) supplemented with EASYpack Protease Inhibitor Cocktail and Pierce Phosphatase Inhibitor Mini Tablets to form the SDS fraction.^63^ The protein concentration of both fractions was quantified with a Pierce BCA Protein Assay Kit (Catalog No. 23227, Thermo Fisher Scientific) and normalized to equal amounts by dilution with ddH_2_O. Samples were snap‐frozen in liquid N_2_, then stored at −80°C. The amount of tau in supernatants, triton, and SDS‐fractions was quantified with Invitrogen Tau (Total) ELISA as described earlier.

Medium and cell lysate aliquots were shipped on dry ice for RT‐QuIC analysis by Alessia Santambrogio at the Yusuf Hamied Department of Chemistry, University of Cambridge. K11 tau (tau residues 244–394) was used as tau seeding substrate. One aliquot of lyophilized K11 purified from *E. coli* was dissolved in 1 mL of 8 M GuHCl (Catalog no. G3272, Merck) prior to size‐exclusion chromatography (SEC) separation on a Superdex 75 10/300 GL column (Catalog no. 17517401, Cytiva) equilibrated in 20 mM sodium phosphate, 200 mM NaCl, pH 7.4. 50 µL/well total volume reactions were prepared in a 384‐well optical BTM Polybase Black plate (Catalog No. 242764, Thermo Fisher Scientific) by adding the individual samples (i.e., the Triton/SDS cell lysate fractions and the CM) in respective 1:10,000 and 1:100 final dilutions to the reaction buffer. The reaction buffer was composed of 3 µM K11 tau monomer, 10 µM ThT, 500 mM Na_2_SO_4_, and 10 mM HEPES buffer at pH 7.4. Reactions were transferred to a 384‐well Nunc microplate (Catalog No. 242764, non‐treated polymer base) covered with aluminum sealing cover to prevent evaporation and subjected to rounds of 60 s shaking (500 rpm, orbital) and 60 s rest with periodic ThT readings every 15 min at 37°C in a FLUOstar Omega lite microplate reader (BMG labtech).

### 2.24 Tau seeding assay in fluorescence resonance energy transfer (FRET) biosensor cell line

A tau RD P301S FRET biosensor cell line (ATCC, CRL‐3275) was used to evaluate the seeding competency of human recombinant 2N4R tau fibrils and the presence of seed‐competent tau species in iMGL‐derived samples.^64^ This biosensor consists of HEK293T cells that stably express tau RD harboring the P301S mutation fused to either Cyan Fluorescent Protein (CFP) or Yellow Fluorescent Protein (YFP). Internalization of tau seeds induces the aggregation of the tau reporter proteins, producing a FRET signal that can be measured by flow cytometry. Cells were plated in 96‐well plates at 25,000 cells per well. The following day, transfection complexes were prepared by combining the transfection reagent mix (1 µL Lipofectamine2000 + 9 µL OptiMEM) with 10 µL of CM or protein mix (50 nM rTauF, 1 µg of Triton/SDS lysates or 2 µg of EVs diluted in OptiMEM). Liposomes were incubated for 20 min at RT before being added to the cells. At 48 h after transfection, cells were lifted with StemPro Accutase and fixed with 2% PFA for 10 min at RT. BD Fortessa X20 was used to perform FRET flow cytometry. To measure CFP and FRET signals, cells were excited with a 405‐nm laser, and fluorescence was detected with 405/50 nm and 525/50 nm bandpass filters, respectively. The FRET‐positive gate was adjusted to cells that were transfected with lipofectamine alone. Each sample was tested in triplicates, and 10,000 events per replicate were analyzed using FlowJo version 10 software. Data are represented as integrated FRET density (percentage of FRET‐positive cells multiplied by the median fluorescence intensity of FRET‐positive population). Integrated FRET density from iMGL samples were normalized to total tau levels in each analyzed fraction, which was quantified by ELISA. To visualize intracellular tau inclusions in the FRET‐positive population, cells were imaged using Amnis ImageStreamX MkII and IDEAS 6.2 software.

### 2.25 Tau seeding assay in iNeurons

Human iPSCs were differentiated into cortical neurons by the forced expression of the neuronal transcription factor neurogenin 2 (Ngn2), as previously described,^65^ with slight modifications. iPSCs (125,000 cells/cm^2^) co‐expressing the reverse tetracycline‐controlled transactivator (rtTA) (pUbiq‐rtTA, 19780, Addgene) and Ngn2 under the TetO promoter (pTetO‐Ngn2‐T2A‐Puro, 52047, Addgene) constructs were plated in 96‐well plates precoated with 0.01% poly‐L‐ornithine and 10 µg/mL laminin. Cells were grown in Neurobasal plus medium (A3582901, Thermo Fisher Scientific) supplemented with 10 ng/mL human brain‐derived neurotrophic factor (450‐02‐10, Peprotech), 0.2 µM L‐ascorbic acid (A0278, Sigma‐Aldrich), 10 ng/mL human NT‐3 (450‐03‐10, Peprotech), 3.3 µg/mL geltrex (A1413302, Thermo Fisher Scientific), GlutaMAX (35050061, Thermo Fisher) and B‐27 plus (A35828‐01, Thermo Fisher) (Table S4). Doxycycline (4 µg/mL, D9891, Sigma–Aldrich) was added on DIV0 to induce TetO‐dependent Ngn2 expression and kept in the cultured until the end of the experiment. The media was also supplemented with 10 µM ROCK inhibitor (Y‐27632, Catalog No. 1201029, Abcam) and kept in culture until DIV4. After 24 h puromycin (A1113803, Thermo Fisher Scientific), selection was performed on DIV2 and DIV3 at 2 and 3 µg/mL, respectively.

Adeno‐associated viral (AAV) particles expressing human 0N4R P301S tau fused to Venus (AAV2.1/2.2‐hSyn‐TauP301S‐Venus) were generated by the McEwan lab. Briefly, chimeric AAV1/2 capsid particles encoding P301S tau‐venus under the hSyn promoter were generated by transfection of AAVPro HEK293T cells (632273, Takara), followed by two rounds of iodixanol (D1556, Sigma‐Aldrich) gradient ultracentrifugation as previously described.^66^ Viral titers and purity were assessed using qPCR for the AAV Inverted Terminal Repeat and SDS‐PAGE, respectively. On DIV4, iNeurons were transduced with the AAV particles. On DIV5, a full medium change was performed and 1 µM AraC (C1768, Sigma‐Aldrich) was added to prevent the growth of undifferentiated cells. Half of the medium was changed every 3 days.

At DIV11, iNeurons were transfected using Lipofectamine200 with 2 µg of EVs as described earlier. At 65 h after transfection, iPSC‐neurons were fixed with 100% pre‐chilled methanol at −20°C for 15 to remove any soluble tau^9^ and immunostained with anti‐MAP2 and anti‐AT8 as described earlier. The integrated Venus or AT8 density was calculated as the product of Venus‐ or AT8‐positive cells and mean Venus or AT8 intensity, normalized to the number of MAP2‐positive cells. The percentage of co‐localization was calculated as the ratio of the number of cells with more than 50% overlap of Venus and AT8 signals to the number of MAP2‐positive cells, times 100.

### 2.26 Statistical information

Statistical analyses were performed in GraphPad Prism version 9.0 (GraphPad Software Inc.) using one‐way ANOVA, two‐way ANOVA, or Student's *t*‐test, with Tukey's, Dunnett's, or Šídák's multiple comparison tests, as appropriate. Specific methods are detailed in individual figures. Data are presented as mean ± standard error of the mean (SEM), unless stated otherwise. Significance was defined as *p ≤ 0.05, **p ≤ 0.01, ***p ≤ 0.001, ****p ≤ 0.0001. n.s. = not significant.

## 3 RESULTS

### 3.1 iMGL take up tau in monomeric and aggregated form

Tau uptake was initially explored using healthy control‐derived iMGL by confocal microscopy (Figure 1A). Recombinant, full‐length 2N4R tau monomer (rTauM) was purified following an initial Triton X‐114 on‐column wash to remove endotoxin from the lysate before it could strongly associate with tau. Characterization showed it to have endotoxin levels of <0.01 EU/mL (at a tau concentration of 1 mg/mL) and to be seed‐competent when fibrilized with heparin (Figure S1A–G). When fed to iMGL, monomer, fibrils (rTauF), and human brain‐derived tau (hTau, Figure S2A–E) all showed signal overlap with CellTracker‐labeled iMGL cytoplasm (Figure S3A) by immunocytochemistry, with tau fibrils also visibly lining the cell periphery. To provide high‐resolution visual evidence of tau fibril interaction with the cell surface, rTauF‐fed or vehicle‐treated iMGL cells were imaged using scanning electron microscopy (Figure 1B, blue arrow indicates a fibril on the iMGL surface). Tau fibrils appeared surrounded by plasma membrane protrusions, suggesting active engagement of the plasma membrane during the fibril uptake process (Figure 1B, red arrow). Additional scanning and TEM from iMac confirm fibrillar structures associated with protrusions and phagocytic cups (Figure S3B) only on rTauF‐fed cells.

### 3.2 LRP1 and HSPGs mediate tau uptake by iMGL

Tau monomer fed to iMac and iMGL co‐localized with LRP1 by confocal microscopy, while fibrils co‐localized less strongly (Figure 1C [iMGL], Figure S3C [iMac]). Tau internalization was significantly reduced by the LRP1 competitive binder sRAP (with less inhibition of fibrils than monomer; Figure 1D) and also by LRP1 knockdown using LentiCRISPR (vs an intergenic region‐targeting control gRNA; Figure S4A,B). In control cells (vehicle/INTG gRNA), internalized tau was detected in the surface LRP1‐negative cell population as a result of receptor internalization upon association with tau. This effect was significantly lower in cells treated with sRAP or LRP1 gRNA (Figure S4B righthand graph). Tau internalization by iMGL was also significantly blocked by heparin, which binds HSPGs, with tau monomer being inhibited less strongly than fibrils (Figure 1E). Fractalkine (CX3CL1, which binds to CX3CR1 on myeloid cells) had no significant effect on tau uptake (Figure S4C).

A recent study demonstrated that tau uptake by iPSC neurons depends also on kinase LRRK2, aside from LRP1.^67^ LRRK2 is a large, multidomain, multifunctional protein, mutations of which are typically linked with familial and sporadic forms of PD.^68^, ^69^, ^70^ Interestingly, the G1029S LRRK2 mutation, which increases the kinase activity two‐ to eight‐fold, has been shown to increase extracellular tau uptake and transmission in neurons.^71^ These findings suggest that aside from its role in PD, LRRK2 may also influence tau propagation in tauopathies. As LRRK2 is highly expressed in microglia, particularly under inflammatory conditions,^43^ and has been implicated in regulating phagolysosomal clearance in myeloid cells,^72^ we next assessed the potential role of LRRK2 in tau uptake. APOE genotype has been shown to influence LRP1‐mediated tau uptake and microglial tau responses^73^, ^74^ – cell lines used were balanced for APOE4 gene dosage (one control and one LRRK2 donor are heterozygous for APOE4). iMac harboring the G2019S LRRK2 mutation took up significantly more tau monomer, while LRRK2 knockout had the opposite effect (Figure S5A). LRRK2 puncta could be visualized overlapping with LRP1–rTauM complexes in WT cells (Figure S5B). LRRK2 knockout significantly reduced surface LRP1 levels (Figure S5C,D). Upon addition of tau, surface LRP1 decreased significantly in G2019S cells but accumulated significantly in LRRK2 KO cells, suggesting that LRRK2 KO cells have a reduced ability to traffic LRP1–tau complexes from the surface to the cell interior (Figure S5C,D).

### 3.3 Tau fibril challenge promotes downregulation of homeostatic and major histocompatibility complex (MHC) gene expression and upregulation of chemokine gene expression in iMGL

To explore the response of iMGL to tau exposure, we initially looked for secretion of the inflammatory cytokines IL‐1β and IL‐6 by ELISA. No significant response to 24 h rTauM, rTauF, or hTau was observed, although iMGL did respond, as expected, to the positive control stimulation LPS (Figure S6A,B).

**FIGURE 2:**
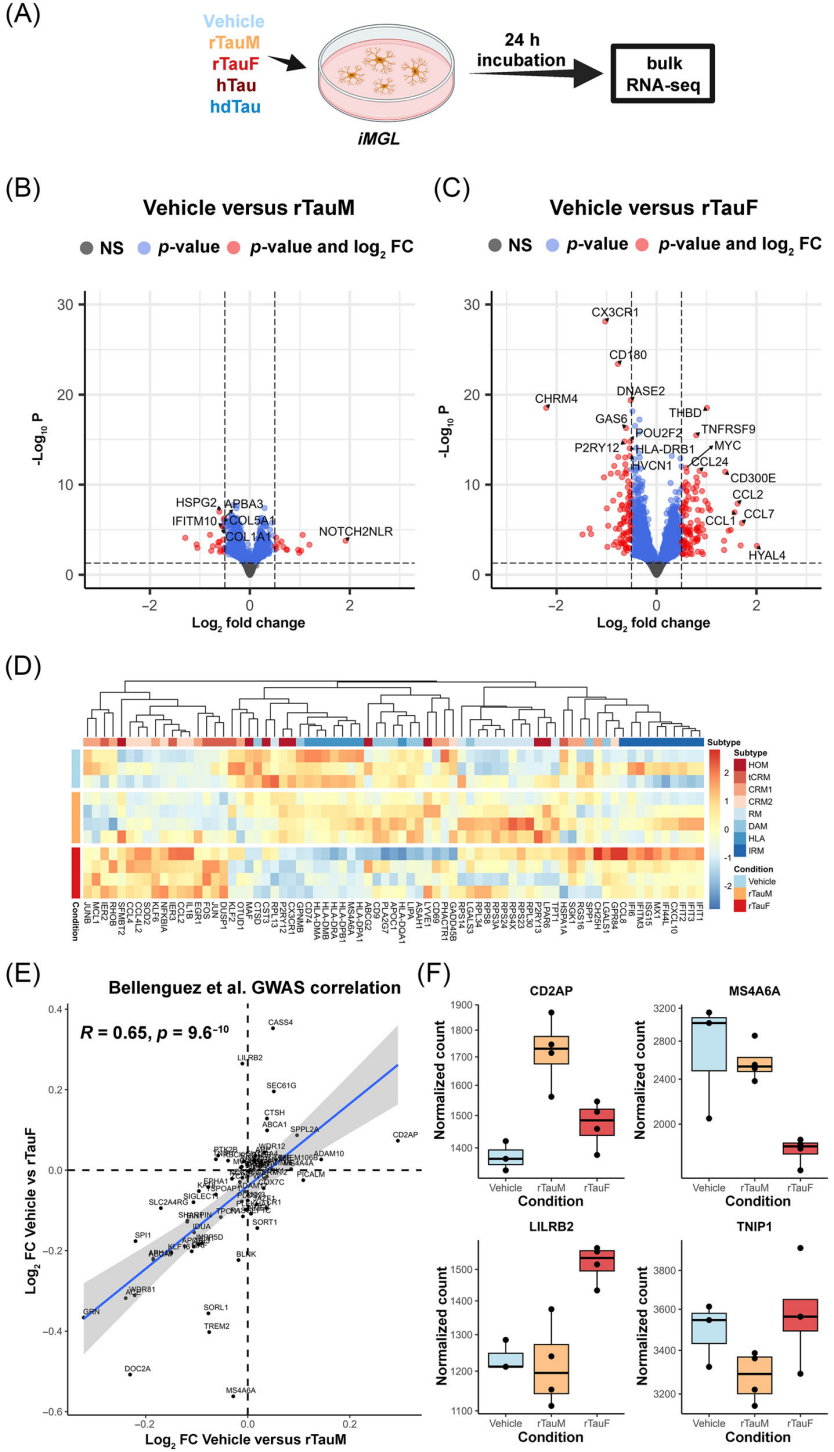
Transcriptomic analysis of iMGL exposed to rTau. (A) Experimental overview of rTau and hTau iMGL exposure for bulk RNA‐seq. (B) DESeq2 volcano plot of vehicle versus rTauM hits. iMGL treated with rTauM show little expression changes when compared to vehicle control. Thresholds are log_2_FC +0.5 or −0.5, and *p* value cut‐off at 0.05. Note these are non‐FDR‐corrected *p* values. (C) DESeq2 volcano plot of vehicle versus rTauF hits. iMGL treated with rTauF show larger expression changes than that of rTauM. Thresholds are log_2_FC +0.5 or −0.5 and *p* value cut‐off at 0.05. Note these are non‐FDR‐corrected *p* values. (D) Heatmap of marker genes of iMGL subtypes described by Mancuso et al. Normalized expression scaled by gene is shown. (E) Correlation of log_2_FC of AD GWAS genes from Bellenguez et al. across the vehicle versus rTauM and vehicle versus rTauF. (F) Example AD GWAS genes showing unique expression changes in only rTauM or rTauF in both directions. *CD2AP*, upregulated in rTauM (log_2_FC 0.29, FDR < 0.01). *LILRB2*, upregulated in rTauF (log_2_FC 0.26, FDR < 0.01) *MS4A6A*, downregulated in rTauF (log_2_FC −0.56, FDR < 0.01), *TNIP1*, trending toward downregulation in rTauM (log_2_FC −0.0.063, FDR > 0.05). Normalized count data shown, points indicate individual samples. AD, Alzheimer's disease; FDR, false discovery rate; GWAS, genome‐wide association study.

We therefore went on to use a global approach (RNA‐seq) to determine what, if any, iMGL responses were induced by tau exposure (Figure 2A). iMGL exposed to monomeric tau (rTauM) resulted in very few changes to gene expression versus vehicle control (Figure 2B), with only five genes downregulated (*HSPG2*, *IFITM10*, *COL1A1*, *COL5A1*, and *APBA3*, log_2_FC < −0.5, FDR < 0.05) and no genes reaching the threshold for upregulation. A much greater change was observed when iMGL were exposed to fibrillar tau (rTauF, Figure 2C), with 87 genes downregulated and 96 genes upregulated, including notable downregulation of homeostatic markers *CX3CR1* and *P2RY12*. In contrast, the top upregulated genes were linked to cytokine and chemokine responses, notably *CCL7*, *CCL2*, *CCL1*, *CXCL5*, *NLRP3*, and *IL1B* gene upregulation reached significance (log_2_FC 0.188, *p*adj = 0.0486; log_2_FC 0.932, *p*adj = 0.000364; Supplementary Data S1 and Supplementary Data S2). Gene Ontology (GO) analysis of vehicle versus rTauF DEGs showed an overrepresentation of chemokine and chemotaxis pathways in the upregulated gene set and MHC‐associated genes in their downregulated counterparts (Figure S6C,D, Supplementary Data S3). Of note, ESCRT pathway genes were not substantively dysregulated (only *VPS28* showed a significant change, in rTauM versus control, log_2_FC –0.215, *p*adj = 0.00303; Figure S6E), and MAPT transcripts were barely detectable (Figure S6F).

Next, we explored whether rTau exposure resulted in microglial subtype switching by examining the expression of a panel of known subtype marker genes^60^ from xenotransplanted iMGL cells (Figure 2D). Expression of genes associated with cytokine response microglia (CRM) and interferon response microglia (IRM) increased following exposure to rTauF, with downregulation of HLA and homeostatic (HOM) markers (mapping to the gene ontology analysis). In contrast, microglia exposed to rTauM had an expression profile similar to that of vehicle, apart from slight upregulation of ribosomal microglial (RM) genes.

Examination of the expression of the most recently published AD GWAS loci^27^ (Figure 2E) showed a positive correlation between vehicle versus rTauF and vehicle versus rTauM log_2_FC (*R* = 0.65, *p* = 6.9 × 10^−6^), suggesting that AD‐risk gene expression changes are universal to tau exposure and are not aggregate specific. However, we identified several AD‐risk genes that showed an effect only in rTauM exposure (*CD2AP*, log_2_FC 0.29, FDR < 0.01, *TNIP1*, log_2_FC −0.063, FDR > 0.05) or only in rTauF exposure (*MS4A6A*, log_2_FC −0.56, FDR < 0.01, *LILRB2*, log_2_FC 0.26, FDR < 0.01) (Figure 2F), indicating specific expression changes dependent on rTau conformation.

**FIGURE 3:**
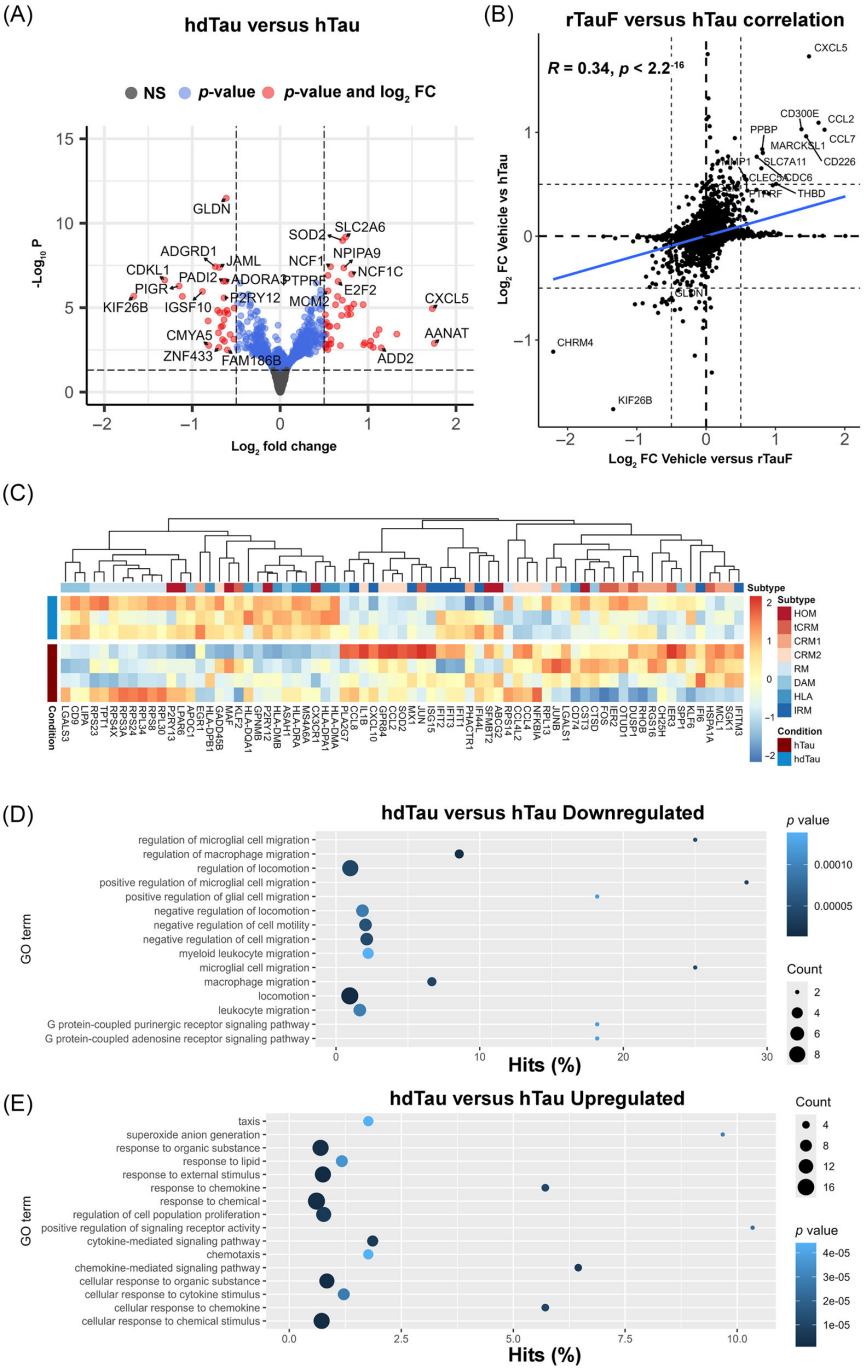
Transcriptomic analysis of iMGL exposed to hTau. (A) DESeq2 volcano plot of hdTau versus hTau hits. iMGL treated with hTau show similar level of expression changes to vehicle versus rTauF. Thresholds are log_2_FC +0.5 or −0.5 and *p* value cut‐off at 0.05. Note these are non‐FDR‐corrected *p* values. (B) The log_2_FC between vehicle versus rTauF and hdTau versus hTau show some correlation of gene expression changes regardless of tau origin. (C) Heatmap of marker genes of iMGL subtypes described by Mancuso et al. Normalized expression scaled by gene is shown. (D) GOSeq of downregulated genes identifies enrichment in pathways for negative regulation of chemotaxis, locomotion, and G‐coupled protein receptor pathways. (E) GOSeq of upregulated genes identified enrichment in pathways for chemotaxis, DNA replication/proliferation, and various chemical response pathways. FDR, false discovery rate; iMGL, induced pluripotent stem cell (iPSC)‐derived microglia.

We then examined the effect of exposure of iMGL cells to tau purified from human AD brain (hTau) to assess shared transcriptional responses between hTau and rTauF. To control for all material present in the AD brain‐derived preparation, we also exposed iMGL cells to the same preparation that had been immuno‐depleted to reduce the level of tau (hdTau) (Figure S7A–C). Comparison of vehicle‐exposed iMGL cells to either hTau or hdTau exposure identified substantial gene changes regardless of tau depletion state (Figure S7D). To correct for the non‐tau‐specific background, we then compared hTau to hdTau to uncover tau‐specific iMGL responses. This resulted in 29 upregulated genes (log_2_FC > 0.5, FDR < 0.05) and 23 downregulated genes (log_2_FC < −0.5, FDR < 0.05) (Figure 3A). Genes involved in microglial homeostatic signaling were downregulated (*CX3CR1*, *P2RY12*, *OLFML3*) and chemotaxis genes were upregulated (*CCL2*, involved in tissue recruitment of blood monocytes, and *CXCL5*). NLRP3‐inflammasome‐associated genes were not significantly upregulated in hTau versus hdTau, although *IL1B* was significantly upregulated in hTau versus vehicle (log_2_FC 0.768, *p*adj = 3.38 × 10^−8^, Supplementary Data S1 and S2). There is a modest correlation in directionality in log_2_FC between vehicle versus rTauF and hdTau versus hTau (*R* = 0.34, *p* < 2.2 × 10^−16^), indicating shared signal regardless of tau origin (Figure 3B). Notably, the top correlated upregulated genes are all involved in chemotaxis (*CXCL5*, *CCL2*, *CD300E*, *CCL7*, *CD226*), and the two most downregulated genes (*CHRM4*, *KIF26B*) in intracellular signaling.

Examining hTau‐challenged iMGL subtypes, hTau showed an upregulation of CRM and IRM subtype genes and downregulation of HOM, DAM, and RM subtypes (Figure 3C), similar to the subtypes observed for rTauF. Finally, GO analysis for hTau identified similar pathways to rTauF, notably upregulation of chemotaxis‐positive genes and downregulation of chemotaxis inhibitor pathways (Figure 3D,E).

Together, this shows that while monomeric tau challenge has negligible effects on iMGL gene expression, tau fibrils, whether recombinant or brain‐derived, induce downregulation of homeostatic genes, upregulation of chemokine genes, and a shift toward chemokine and interferon response subtypes.

### 3.4 Tau fibrils are degraded poorly by iMGL and can escape into the cytoplasm

**FIGURE 4:**
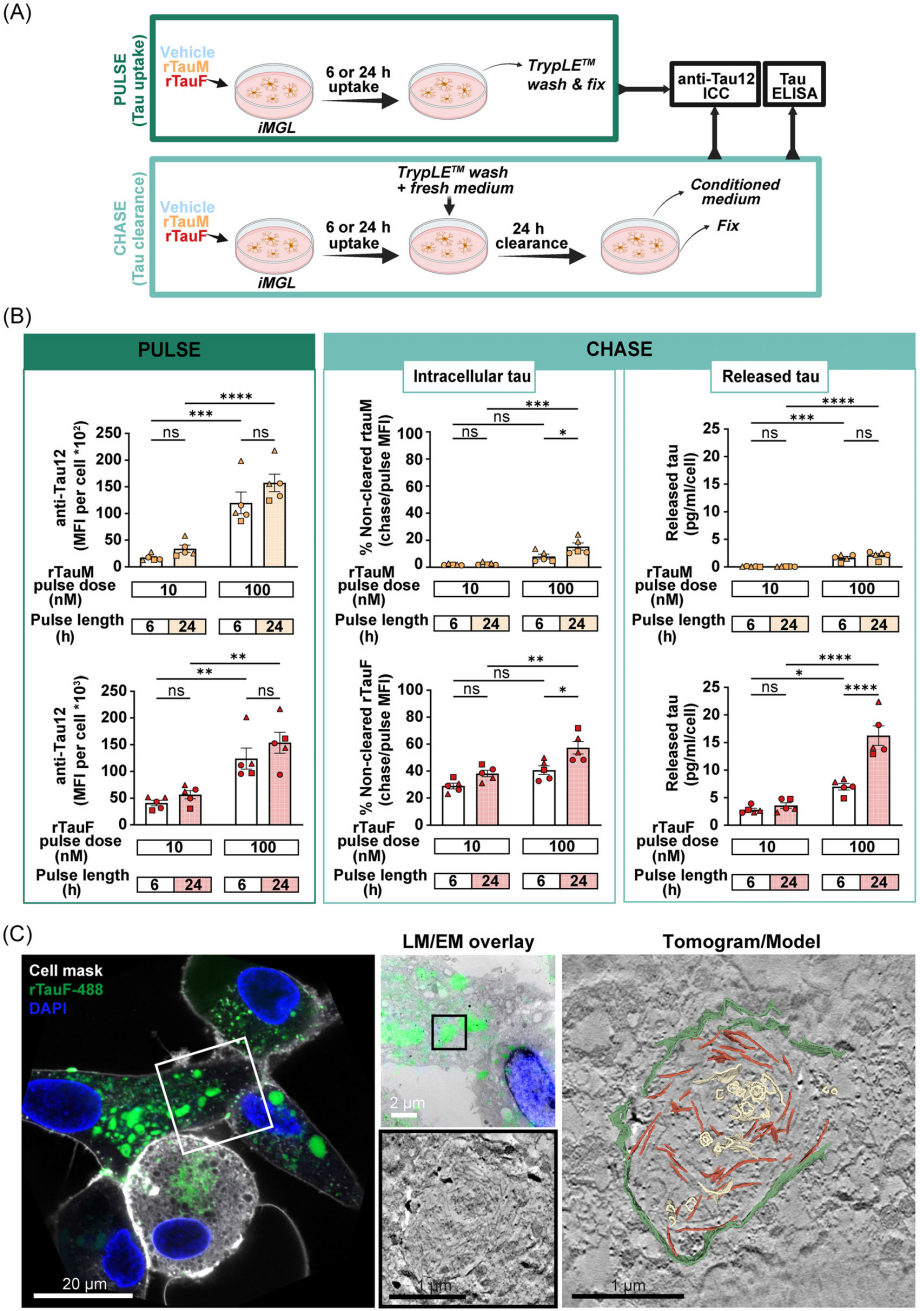
Fibrillar tau escapes degradation and is freely present in the cytosol. (A) Schematic of tau pulse‐chase experiment. (B) rTauM/rTauF internalization (pulse) and clearance (chase) by iMGL. Intracellular tau levels in pulse and chase were quantified by anti‐Tau 12 immunostaining (MFI) relative to the number of cells. The amount of released tau in the CM was determined with total tau ELISA relative to the number of cells. *N* = 1–2 in three control cell lines, two‐way ANOVA with Tukey's multiple comparisons test, mean ± SEM, *p < 0.05, **p < 0.01, ***p < 0.001. (C) CLEM overlay (left) and region of interest (EM data boxed region) of iMGL incubated overnight (16 h) with Dylight‐488‐labeled rTauF. Tomogram slice with 3D model (right) of the same region indicates rTauF (red) and membranous structures (yellow) within a partial enclosing membrane (green). CLEM, correlative light and electron microscopy; CM, conditioned medium; ELISA, enzyme‐linked immunosorbent assay; EM, electron microscopy; iMGL, induced pluripotent stem cell (iPSC)‐derived microglia; SEM, standard error of the mean.

To better understand the ability of iMGL to degrade tau, we challenged iMGL cells with low or high doses of tau (pulse), then removed soluble exogenous tau with a mild TryplE wash^63^ and assessed the remaining tau content in the cells, in addition to released tau, by Immunocytochemistry and ELISA after defined periods (chase) (Figure 4A). There is no treatment compatible with cell viability that could selectively and completely remove cell surface‐bound, protease‐resistant insoluble tau, and this remains a general technical limitation for conducting pulse‐chase assays. As a control condition, to determine whether tau detected in the supernatant reflected iMGL‐processed and released species rather than residual fibrils adhering to the culture plate surface, cell‐free wells of a 96‐well plate were coated with a high concentration (10 µg/mL) of rTauM or rTauF for 6 or 24 h, then subjected to the TrypLE/PBS wash procedure before incubating with iMGL medium. After 24 h, tau levels in the medium were below the total tau ELISA detection limit (<31 pg/mL), confirming that contamination from residual plastic‐adherent tau does not contribute to the released‐tau measurements.

The pulse phase showed dose‐ and time‐dependent uptake of rTauM and rTauF, with approximately 10‐fold more signal detectable in the rTauF cells. The majority of rTauM was degraded within 6 h of chase, and relatively little was released into the supernatant. Conversely, rTauF was poorly degraded, with the majority of internalized tau still present in the cells, even at the lower dose and longer chase time, with the amount released into the supernatant increasing significantly with dose, pulse, and chase time (Figure 4B). Protease inhibition significantly increased the amount of rTauF detected in the cells, implying at least some capacity to degrade tau fibrils, likely by lysosomal enzymes (Figure S8A,B).

To understand whether internalized tau fibrils remained in the endophagolysosomal system of iMGL, we performed CLEM using 488‐labeled tau fibrils. Electron tomography of 488‐positive regions revealed accumulations of tau fibrils within partial or damaged membrane‐bound compartments, suggesting that fibrils can compromise the integrity of their transport vesicles and escape into the cytosol (Figure 4C, Figure S8C, Supplementary Videos S1 and S2).

### 3.5 Tau‐fibril‐challenged iMGL phosphorylate tau and undergo extensive phosphoproteome remodeling

**FIGURE 5:**
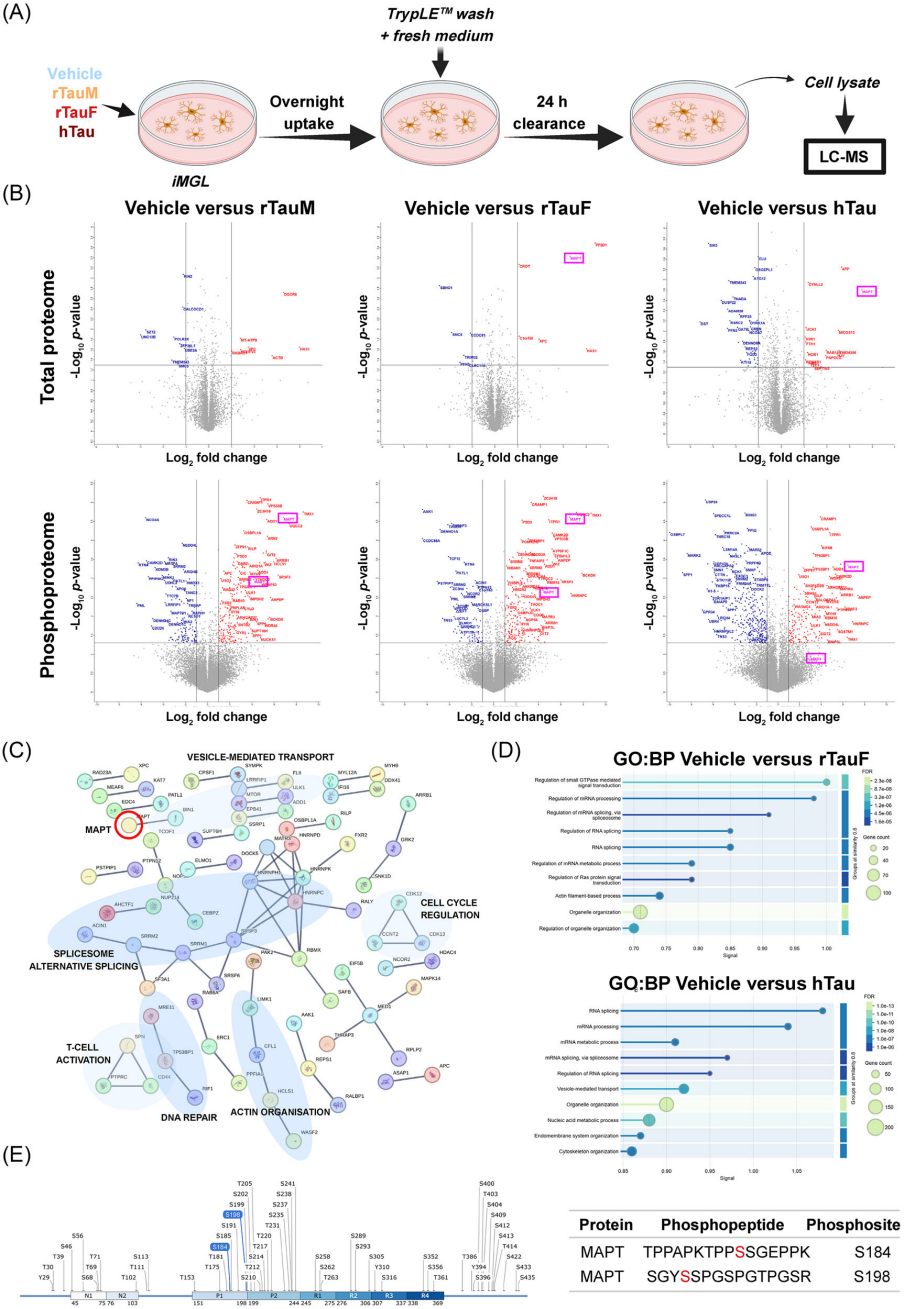
Proteomics and phosphoproteomics of iMGL lysates. (A) Experimental workflow. Cells were treated overnight with rTauM, rTauF, or hTau and allowed to process internalized tau for 24 h. Cell lysates, CM, and EVs were collected for proteomic analysis. (B) Differentially expressed proteins (upper panel) and phosphopeptides (lower panel) in iMGL treated with rTauM, rTauF, or hTau compared to vehicle‐treated cells. Vertical line: fold change above 2; horizontal line: significant proteins (−log*p* > 1.301 or *p* < 0.05). MAPT highlighted in pink boxes. (C) STRING analysis of differentially expressed phosphopeptides in iMGL treated with rTauF. Only high‐confidence interactions (interaction score > 0.9) are displayed. MAPT highlighted with red circle. (D) Classification of differentially expressed phosphopeptides in rTauF and hTau iMGL based on GO biological processes. (E) Map of all 2N4R tau phosphosites, with the upregulated phosphoresidues (S184 and S194) found in iMGL treated with rTauF highlighted in blue (image created with SnapGene). MAPT phosphopeptide sequences are indicated in table. CM, conditioned medium; EV, extracellular vesicle; GO, Gene Ontology; iMGL, induced pluripotent stem cell (iPSC)‐derived microglia; MAPT, microtubule‐associated protein tau.

AD brains have been characterized by proteomic and phosphoproteomic analysis to identify biochemical and cellular pathways altered during disease progression.^75^, ^76^, ^77^, ^78^ However, it is still unclear how the proteome of each brain cell type is modified in AD. Here, using mass spectrometry, we analyzed the proteome and phosphoproteome of iMGL cells treated with rTauM, rTauF, and hTau (Figure 5A). Samples clustered primarily by cell line rather than cell treatment by principal component analysis (PCA), indicating that the main source of variation in our data arises from the genetic background of each donor (Figure S9A). A total of 8101 proteins were identified in the cell lysates (Supplementary Data S4), but no major differences were found when comparing vehicle versus rTauM, rTauF, or hTau conditions, with 17, 12, and 58 differentially expressed proteins (DEPs) found in each, respectively (Figure 5B, Supplementary Data S4).

In agreement with our RNA‐seq data (Figure S6F), tau (MAPT) was not identified in untreated iMGL. Tau was only detected by proteomics when cells had been treated with exogenous rTauF or hTau, and notably no other MAPs changed as a result of the tau treatment (Figure S9B), indicating that, if present at all, endogenous tau protein expression in iMGL is extremely low. This contrasts with a recent paper^79^ that proposed that the level of tau expression in iPSC‐microglia had biological relevance. Notably, the absence of tau in lysates from iMGL treated with rTauM indicates the ability of microglia to fully degrade monomeric, but not fibrillar, tau (Figure 5B).

Mapping the peptides detected by liquid chromatography tandem mass spectrometry (LC‐MS/MS) onto the 2N4R tau sequence (Figure S9C,D, Supplementary Data S4) showed no peptides corresponding to the N‐terminal domain of tau, but peptides were detected across the proline‐rich domain, the microtubule‐binding domain, and the C‐terminus for rTauF and hTau in the cell lysates. Abundance was relatively higher for peptides in the core regions of the protein (Figure S9D).

Phosphoproteomic analysis of microglia cell lysates identified 43,456 phosphopeptides across all samples (Supplementary Data S5), with 329, 308, and 547 differentially expressed phosphopeptides identified in rTauM, rTauF, and hTau samples, respectively (Figure 5B, Figure S10A–C, Supplementary Data S5). STRING network and GO biological process (BP) analysis identified functional categories common to all tau‐treated conditions studied, including vesicle‐mediated transport, actin reorganization, and alternative splicing (Figure 5C,D, Figure S10A,B). Notably, phosphosites associated with actin cytoskeleton remodeling (HCLS1, WASF2), autophagy (MTOR, ULK1), endolysosomal pathway (RAB42, RAB10, RILP), vesicle sorting (BIN1), and mRNA processing (SRSF3, HNRNPC) were upregulated in microglia treated with rTauF and hTau (Figure 5C).

Tau phosphorylation is highly correlated with tau pathology in AD and other tauopathies.^77^ Whether microglia could be contributing to tau phosphorylation remains unknown. Since iMGL cells do not endogenously express tau, and *E. coli*‐derived recombinant tau (rTauF) is not post‐translationally modified (Supplementary Data S4 and S6), we could observe in our phosphoproteomics dataset that tau residues S184 and S198 were phosphorylated in iMGL treated with rTauF (Figure 5B,E, Supplementary Data S5). These two phosphorylated sites were also found in the human AD tau pooled sample (Supplementary Data S3) and in hTau‐treated microglia (Figure 5B, Supplementary Data S5). The two specific phosphorylations (pS184 and pS198) added by microglia to internalized rTauF have been identified in AD (but not in healthy) brains.^80^ The identification of the kinase(s) responsible merits further exploration. These phosphorylations could have significance, as they are in the proline‐rich, microtubule‐binding domain, and pS198 is increased at early Braak stages, correlating with the formation of early misfolded tau oligomers.^81^ The development of sensitive reagents, including antibodies and ELISAs, to accurately quantify these specific phosphorylations would be valuable.

Together this shows that endogenous tau protein is undetectable in iMGL, and that iMGL can degrade exogenous monomeric tau to completion, but internalized fibrillar tau is not efficiently degraded and is actually phosphorylated in iMGL on two specific residues. It also shows that although the total cellular proteome does not change substantively over the relatively short tau‐challenge timeframe studied here, the phosphoproteome shows extensive engagement of cytoskeletal remodeling and metabolic pathways.

### 3.6 Tau‐challenged iMGL cells secrete tau, including associated with EVs

**FIGURE 6:**
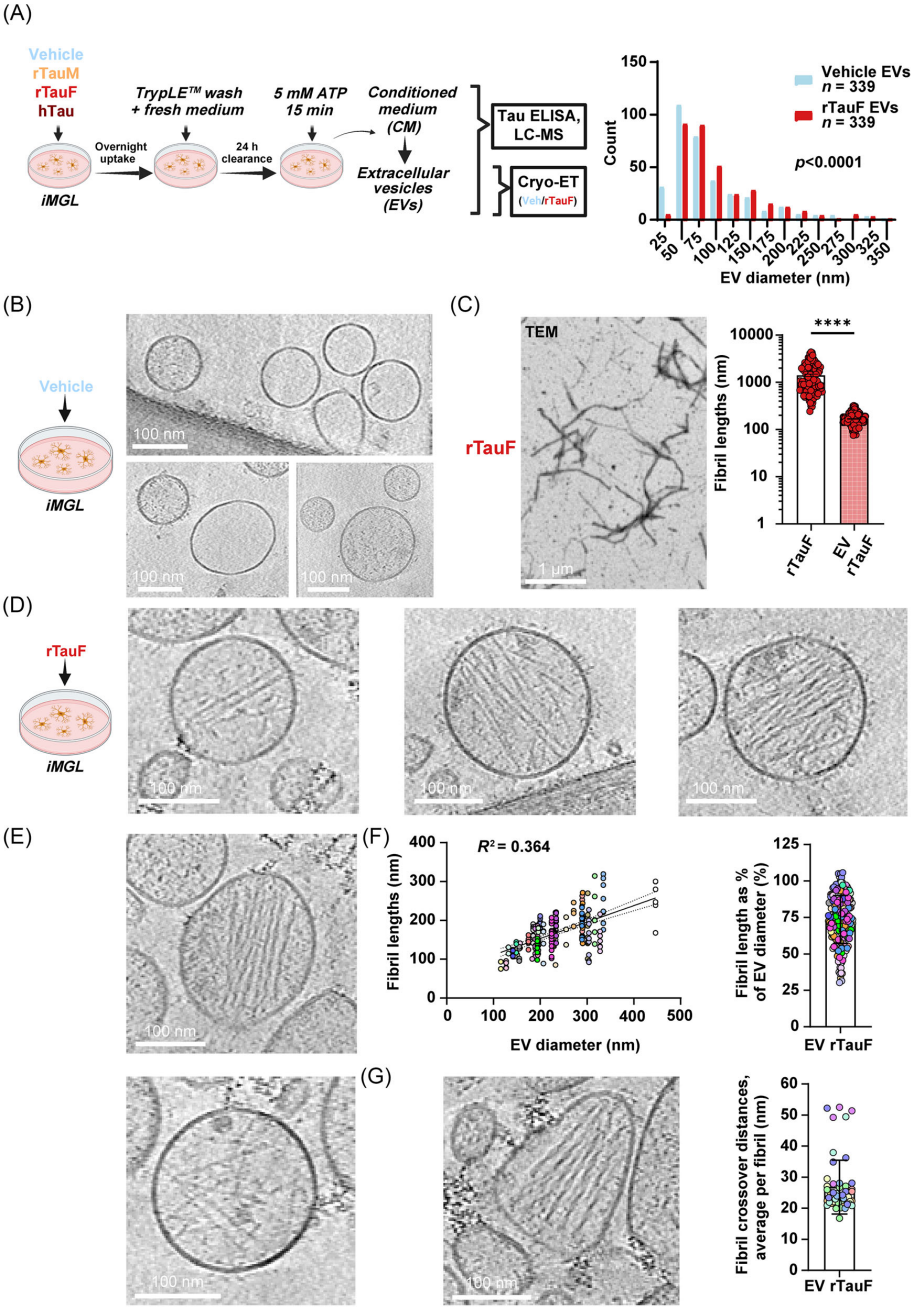
Cryo‐ET reveals tau fibrils packaged inside secreted EVs. (A) Experimental overview (left). Cells were treated overnight with rTauM, rTauF or hTau, and allowed to process internalized tau for 24 h. iMGL were stimulated with 5 mM ATP (15 min) to enhance the release of EVs to the CM. Diameter distribution of EVs secreted from vehicle or rTauF treated iMGL cells (right). Data are represented as frequency distributions of *n* = 339 EVs measured from 42 (vehicle EVs) or 22 (rTauF EVs) tomograms. Kolmogorov‐Smirnov test for distribution similarity, ****p < 0.0001. (B) EVs from vehicle‐treated iMGLs. (C) Appearance and length of rTauF used for treatments, compared to fibril lengths measured inside EVs. Data are represented as mean ± SEM of *n* = 113 fibrils (rTauF) measured across seven TEM images and *n* = 223 fibrils (rTauF EV fibrils) measured from 35 EV tomograms. Two‐tailed Mann–Whitney test. ****P < 0.0001. (D) Example images of low, medium, and high amounts of fibrils contained in EVs from rTauF‐treated iMGLs. (E) Example images of fibrils either aligning inside the EVs to a high degree (top) or having more random orientations (bottom). (F) Fibril length as a function of EV diameter (left) or expressed as a percentage of EV diameter (right), same rTauF EV fibril data as displayed in (C). Each dot represents an individual fibril, color‐coded to show which EV they belong to. Linear regression with *R* ^2^ displayed. Data are represented as mean ± SD of *n* = 223 fibrils measured from 35 EV tomograms. (G) Representative image of the subset of EVs that contain visibly twisted fibrils. Quantification of helical cross‐over distances, averaged per fibril. Each dot color represents an individual EV from which several fibrils were often measured. Data are represented as mean ± SD of *n* = 54 fibrils measured from eight EV tomograms. ET, electron tomography; EV, extracellular vesicle; iMGL, induced pluripotent stem cell (iPSC)‐derived microglia; SD, standard deviation; SEM, standard error of the mean; TEM, transmission electron microscopy.

We next followed up on our earlier observation (Figure 4) that showed secretion of internalized tau species by iMGL cells. Following 16 h tau pulse and 24 h chase (Figure 6A), rTauF‐challenged iMGL cells released significantly more tau to the CM than rTauM‐challenged iMGL, as assayed by total tau ELISA. Tau release to the medium in hTau‐treated iMGL was negligible as assayed by this ELISA, an observation that was consistent across CM collected from different centrifugation stages of the EV purification protocol (Figure S11A–C). Tau species are prone to truncation in AD brains,^82^, ^83^ so the epitopes recognized by the ELISA (proprietary commercial information) might no longer be present, potentially leading to an underestimation of released hTau. Indeed, by mapping the peptides detected by LC‐MS/MS onto the 2N4R tau sequence (Figure S9C,D), only one peptide corresponding to the proline‐rich domain of tau was found in the CM from hTau‐treated iMGL, while a broader coverage was found in CM samples from rTauM‐ and rTauF‐treated cells. Proteomic analyses revealed that MAPT was the only DEP in the supernatant of rTauM‐ and rTauF‐treated iMGL (Figure S11D, Supplementary Data S7). Although not statistically significant, MAPT was detected in the CM of hTau‐treated iMGL, and additional DEPs were identified, notably the upregulation of IL‐6 and IL1RN (Figure S11D, Supplementary Data S7).

We went on to examine the protein content of EVs isolated from the CM of tau‐treated iMGL (Figure S11E,F; EV characterization, Figure S12A–D). Using ELISA, tau was detected in EVs secreted by rTauF‐ and hTau‐treated iMGL, but the proportion of tau packaged in EVs was substantially lower than the amount released freely into the CM (0.8% and 2%, respectively, Figure S11C). LC‐MS/MS analysis identified a total of 3740 proteins from our EV samples, including canonical EV markers important for EV formation, targeting and uptake (CD9, CD63, CD81, TSPAN14, MFGE8), cargo sorting (ANXA1/2/4/5/11, HSP90), membrane and vesicle trafficking (RAP1A/B, TSG101), and MHC molecules (HLA‐A/B/C, HLA‐DRB3) (Supplementary Data S5). Markers of cellular organelles not associated with EVs, including calnexin (endoplasmic reticulum), GM130 (Golgi), and cytochrome C (mitochondria), were low in abundance or not detected. rTauM induced only 21 DEPs versus vehicle‐treated iMGL, whereas rTauF and hTau induced 109 and 55 DEPs, respectively (mostly downregulations). MAPT was detectable in EVs from rTauF‐ and hTau‐challenged iMGL, but only reached significance following the hTau challenge (Figure S11E, Supplementary Data S8 and S9).

All this showed that EVs expressing typical markers were successfully isolated from iMGL CM and that tau secreted by fibrillar tau‐challenged iMGL could be detected in supernatants and associated with EVs.

### 3.7 Tau fibrils can be visualized packaged inside EVs secreted by tau‐challenged iMGL

Having demonstrated that fibrillar tau associated with EVs, we considered whether it might be detectable inside EVs released by iMGL. We performed cryo‐ET on purified and concentrated EV samples from iMGL treated with rTauF, comparing them to EVs from vehicle‐treated cells. We did not include EV samples from rTauM‐treated iMGL, as we had demonstrated that it is not detected in EV preparations by mass spectroscopy, nor did we include hTau‐treated iMGL in the cryo‐ET workflow due to the contaminating presence of EV structures in the original brain‐derived hTau preparation (as seen in Figure S2B). The majority of isolated EVs were within the range expected for exosomes (30 to 150 nm diameter),^84^ with a significant increase in size distribution of EVs from rTauF‐treated iMGL versus vehicle (Figure 6A, right‐hand panel). EVs isolated from vehicle‐treated iMGL did not visually contain filaments (95% of tomograms from the vehicle or TauF‐treated datasets were correctly assigned to each group by a blinded independent assessor) (Figure 6B). rTauF fibrils fed to iMGL are at least 10‐fold longer (as conservatively measured from negative stain EM) than within fibril‐containing EVs (cryo‐ET measurements) (Figure 6C), suggesting that the packaged fibrils have undergone partial cleavage inside the iMGL. To examine whether these fibrillar structures were most likely to be tau as opposed to other filamentous proteins (including actin or tubulin), we verified from the EV‐proteomics dataset that only the abundance of tau increased with the treatments and no other candidate filamentous proteins (Figure 11F). The quantity of fibrils inside EVs varied widely, from singular filaments to numerous and densely packed (Figure 6D). Fibrils were frequently aligned in bundles, but also occasionally oriented in opposing directions (Figure 6E). Plotting individual fibril length against the diameter of the EV that contained them revealed a positive correlation (Figure 6F). A subset of EVs contained fibrils with visible helical twists, with crossover distances that aligned well with published cryo‐tomography data from recombinantly produced 2N4R tau^85^ (Figure 6G). We cannot fully rule out that some fibrils seen inside an EV might be, for example, actin, but since visible filaments inside EVs are almost unique to the rTauF condition, the abundances of other filamentous proteins are unchanged across conditions, and subsets of fibrils display helical twists, this corroborating evidence strongly indicates that most fibrils visualized are indeed tau. These results demonstrate that EVs can be a route through which iMGL package and dispose of rTauF. Notably, iMGL seemingly cleave the long rTauF fibrils and form EVs to match this length.

### 3.8 Fibrillar tau secreted in CM and EVs from iMGL is seed‐competent

**FIGURE 7:**
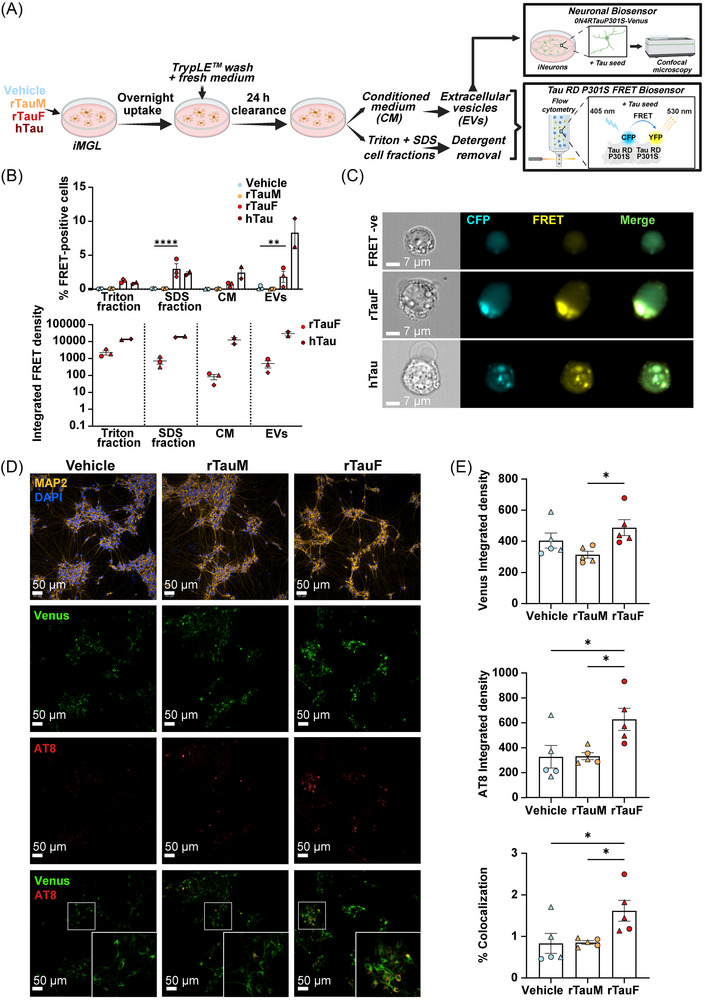
Undigested tau fibrils are present in iMGL cell lysates, CM, and EVs and have differential seeding capacities. (A) Experimental workflow. Cells were treated overnight with rTauM, rTauF or hTau, and allowed to process internalized tau for 24 h. Soluble (triton) and insoluble (SDS) cell fractions, CM and EVs were collected and transfected into tau RD P301S FRET Biosensor HEK cell line. iNeurons expressing 0N4RTauP301S‐Venus were also transfected with EVs. (B) Percentage of FRET‐positive cells and normalized integrated FRET density in Tau RD P301S FRET Biosensor HEK cells incubated for 48 h with Triton/SDS fractions (1 µg), CM (10 µL) or EVs (2 µg) from iMGL treated with rTauM, rTauF or hTau. Data are represented as mean ± SEM of *n* = 1 (run in triplicates) from 2 to 3 control cell lines. Two‐way ANOVA with Tukey's multiple comparison test was used. hTau samples were excluded from the analysis due to small sample size (*n* = 2); **p < 0.01, ****p < 0.0001. (C) Representative images of Tau RD P301S FRET Biosensor HEK cells transfected with EVs isolated from rTauF‐ or hTau‐treated iMGL with or without (upper panel) intracellular tau inclusions. Cells were imaged in suspension by Amnis ImageStreamX MkII. (D) Representative images of iNeurons seeded with EVs from vehicle‐, rTauM‐, or rTauF‐treated iMGL. (E) Quantification of seeded tau aggregation measured by Venus and AT8 integrated density and percentage of Venus/AT8 co‐localization. Data are represented as mean ± SEM of *n* = 2 to 3 (run in duplicates) from two control cell lines. One‐way ANOVA with Tukey's multiple comparison test was used; *p < 0.05. CM, conditioned medium; EV, extracellular vesicle; HEK, human embryonic kidney; iMGL, induced pluripotent stem cell (iPSC)‐derived microglia; SEM, standard error of the mean.

Finally, we assessed the seeding competency of undigested tau within iMGL cell lysates (soluble and insoluble fractions), as well as in the CM and EVs from iMGL treated with vehicle, rTauM, rTauF, or hTau (Figure 7A). First, a K11 RT‐QuIC assay was used to determine the presence of seeding‐competent tau aggregates in the cell lysates and CM (Figure S13A–D). The K11 tau substrate used in the assay is a four‐repeat (4R) tau construct spanning aa residues 244–400 (Figure S13B), previously shown to detect 4R and 3R/4R tauopathy‐associated pathology and differentiate tau conformer strains.^86^ Seeding of recombinant K11 tau substrate by undigested, insoluble rTauF and hTau in SDS iMGL lysates was evident. In comparison, soluble rTauF and hTau in Triton cell lysates, as well as CM from iMGL‐treated with rTauF and hTau, exhibited more residual seeding, with larger ThT amplitude variability (Figure S13C,D). Of note, iMGL samples treated with rTauF versus hTau showed reliably divergent ThT amplitudes, consistent with structural differences previously reported by cryo‐EM^85^ and indicative of faithful propagation of two unique tau amyloid strains through cellular uptake and processing.

We next corroborated our findings using a well‐established cell‐based assay, the Tau RD P301S FRET Biosensor cell line^64^ (Figure 7A). Tau present in the soluble (Triton lysate) and insoluble (SDS lysate) fractions, and CM and EVs from iMGL treated with rTauF and hTau induced aggregation in the FRET Biosensor cell line, seen as an increase in the percentage of FRET‐positive events (Figure 7B). Since the samples included both total and enriched fractions (EVs), the integrated FRET density (calculated as the product of the percentage of FRET‐positive cells and the FRET median fluorescence intensity) was normalized to the total tau levels (pg) detected in each fraction by ELISA. This allowed the direct comparison of intrinsic seeding activity across cellular fractions containing different amounts of tau. ImageStream enabled visualization of intracellular tau aggregate formation in biosensor cells transfected with EVs isolated from rTauF‐ and hTau‐treated iMGL (Figure 7C).

We proceeded to evaluate the seeding competency of EVs isolated from iMGL cultures treated with rTauM or rTauF using a more physiologically relevant novel human neuronal biosensor consisting of iNeurons expressing human 0N4R tau harboring the P301S mutation fused to Venus (Figure 7A). Treated iNeurons were fixed with methanol to remove soluble tau, and Venus and AT8 (pTau S202/T205) antibody signal intensity was quantified, in addition to Venus/AT8 co‐localization. EVs from rTauF‐treated iMGL induced significant tau aggregation in this neuronal biosensor (Figure 7D,E).

These results indicate that tau released following uptake by microglia, including fibrils observed inside iMGL EVs by cryo‐EM, are seed‐competent, with the ability to seed aggregation in human neurons and could play a role in the cell‐to‐cell transmission of tau pathology.

## 4 DISCUSSION

**FIGURE 8:**
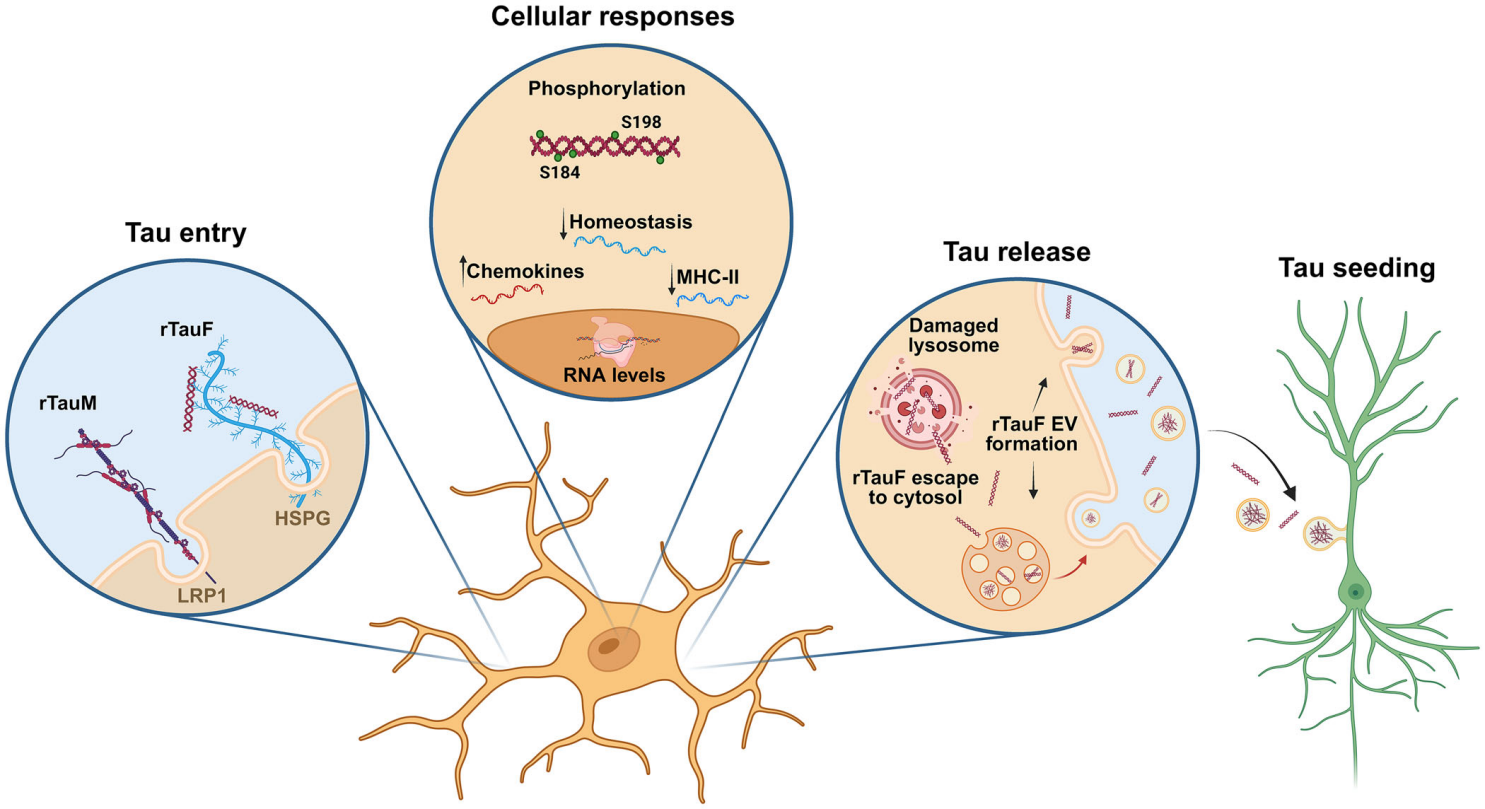
Graphic summary. A summary of tau processing by human iPSC‐microglia defined in this manuscript. Entry of monomeric tau occurs preferentially via LRP1, whereas fibrillar tau entry is in part facilitated by LRP1 and HSPGs. Cellular responses of microglia upon fibrillar tau internalization (both recombinant and human brain‐derived) include upregulation of chemokine and interferon pathway genes, downregulation of homeostatic and MHC‐II genes and proteomic changes mainly affecting phosphorylation states. In particular, phosphorylation of two serines on MAPT were detected. Evidence of tau release included its detection in CM and in EVs both by proteomics and ELISA. Furthermore, cryo‐electron tomography revealed the presence of fibril structures packed into individual EVs and the seeding capacity of this EV‐associated tau was verified in two biosensor systems. CM, conditioned medium; ELISA, enzyme‐linked immunosorbent assay; HSPG, heparan sulfate proteoglycans; iPSC, induced pluripotent stem cell; LRP1, lipoprotein receptor‐related protein 1; MAPT, microtubule‐associated protein tau.

Here we examined the uptake, processing, release, and seeding of tau in the context of human iPSC‐microglia, using endotoxin‐free recombinant tau and AD‐brain‐derived tau. We have demonstrated uptake of monomeric and fibrillar tau via LRP1 and HSPGs and have visualized escape into the cytoplasm of internalized fibrils. We have shown that recombinant or brain‐derived tau fibrils shift iMGL toward chemokine and interferon response subtypes and induce phosphoproteome remodeling. We have demonstrated that endogenous tau is not detectable in iMGL, that iMGL degrade fibrillar tau inefficiently and can phosphorylate undigested tau on specific residues. Finally, we have shown that iMGL release undegraded tau, including secretion as fibrils within EVs, and that these can seed tau aggregation in downstream cells, including neurons, as summarized in Figure 8.

We found that uptake of monomeric tau into iMGL was strongly reliant on LRP1, and only modestly on HSPGs, while uptake of tau fibrils was less dependent on LRP1, and neither form was affected by fractalkine (CX3CR1 ligand). Our LRP1 and HSPG results concur with recent mouse and human microglia data^28^, ^87^ and recent human iNeuron data,^67^, ^88^ demonstrating that the different uptake pathways for tau monomer versus fibrils are conserved across cell types and species. Also, in line with findings from iPSC‐neurons,^67^ we show that the microglial LRRK2 genotype influences tau uptake via LRP1, with G2019S LRRK2 increasing uptake and LRRK2 knockout reducing uptake and perturbing surface LRP1 levels. Since LRRK2 phosphorylates specific Rabs to modulate vesicle trafficking,^43^, ^89^ it is conceivable that LRRK2 regulates trafficking of LRP1 to/from the plasma membrane. Altogether, LRP1, possibly in conjunction with LRRK2, appears to have a broadly conserved role in regulating tau internalization by multiple brain cell types. Given that LRP1 knockdown has been shown to reduce tau spreading in vivo,^17^ perturbations to microglial LRP1 are also likely to influence tau spread by microglia. Mechanistically, this effect would likely stem from altering the total amount of tau internalized by microglia available for subsequent events. We have not directly tested the downstream consequences of LRP1 knockdown on tau processing and re‐release in this study, but reduction in tau uptake associated with LRP1 reduction or loss of function would be predicted to yield proportionally less tau entering the degradative pathways, escaping from damaged lysosomes into the cytoplasm, and entering the secretory pathways. Overall, this places LRP1 as a key determinant of overall intracellular tau burden and subsequent spread.

Endotoxin (LPS) is a strong inducer of TLR4‐mediated signaling, especially in myeloid cells (including microglia), leading to the production of classic inflammatory cytokines. Endotoxin removal from recombinant tau after purification is inefficient, presumably due to the establishment of strong interactions resulting from charge differences between tau and endotoxin.^90^, ^91^ We therefore developed a tau purification protocol that removes the majority of endotoxin straight after bacterial lysis, through an initial on‐column Triton X‐114 wash step. This procedure reduces endotoxin in the final purified tau to negligible levels (Figure S1). We detected minimal microglial response to our recombinant monomeric tau, and while recombinant fibrillar tau induced a transcriptomic shift to chemokine and interferon response subtypes and upregulation of *NLRP3* and *IL1B* transcripts, no significant upregulation of inflammasome‐associated protein products (NLRP3, PYCARD/ASC, CASP1, IL1B, IL18), and phosphopeptide enrichment of these proteins was not detected (Supplementary Data S1–8). Secretion of classic inflammatory cytokines IL‐1β and IL‐6 was not detected by ELISA, IL‐6 release to CM was only detected in the proteomics dataset of hTau‐treated iMGL – this discrepancy is likely due to differences in the sensitivity and dynamic range between the two methods, as the LC‐MS platform can detect low‐abundance peptides with high sensitivity. We did not detect any change in levels of IRF3 phosphopeptides following tau challenge, which would be indicative of cGAS‐STING pathway activation and would lead to IFN type I and IL‐6 secretion.

Pathological tau species were previously indicated to upregulate the production of pro‐inflammatory cytokines in microglia (reviewed in Laurent et al.^92^). Furthermore, several studies specifically reported microglial NLRP3 inflammasome activation in response to pathogenic tau derived from human cells or *post mortem* AD brain tissue.^93^, ^94^, ^95^ The overall muted cytokine response of microglia to tau in our study, and the absence of IL‐1β secretion is therefore notable. This may be attributable to several factors, including (a) the lack of endotoxin contamination in our recombinant and human brain‐derived tau preparations – reviewing the literature reveals that many microglia studies do not state the endotoxin levels in their recombinant tau preps (Table S1), so it is possible that some reported results are confounded by microglial responses to endotoxin (which primes NLRP3 inflammasome); (b) the concentration of tau we used (1 µg/mL, equating to ∼20 nM for monomeric tau and a high picomolar to low nanomolar range for fibrillar tau) was purposefully selected to fall within the likely range that microglia might encounter within the interstitial space^96^, ^97^; and (c) gene expression differences between human and rodent microglia have been consistently demonstrated,^98^, ^99^, ^100^ including divergence in inflammatory pathways and responses to pathological stimuli.^101^ Future studies should systematically compare microglial response to tau species, concentrations, priming conditions, and microglial origin under controlled endotoxin‐tested conditions to clarify the issue.

CLEM identified regions in tau‐fibril‐fed iMGL that contained tau fibrils within partial or damaged enclosing vesicles, suggesting that internalized fibrils can escape into the cytoplasm of iMGL. While we detected phosphorylation of proteins involved in the endolysosomal pathway, we did not detect changes in LRRK2 phosphorylation, or phosphorylation in the switch II domains of Rabs associated with LRRK2, which could lead to activation of effectors, including ESCRT subunit CHMP4B, which has been found to be associated with nanoscale lysosomal membrane tau‐induced damage in other cell types^102^, ^103^ (it should be noted that our proteomics results use iMGL from healthy control iPSC, not LRRK2 variants). ESCRT genes were also not significantly upregulated in the transcriptomic analysis. Assays for lysosomal leakage using acridine orange or sulforhodamine B or recruitment of galectin‐3 to damaged lysosomal membranes did not provide evidence for endolysosomal damage in these cells, possibly due to imaging/temporal resolution limitations (results not shown). Even so, the fact that we also see specific phosphorylation of internalized recombinant tau fibrils and can visualize them packaged into EVs confirms that tau can escape into the cytoplasm. It is in the cytoplasm that tau will most likely be phosphorylated and also be packaged into single‐membrane‐bound EVs.

Since endogenous tau protein was not detectable in iMGL by ELISA or proteomic analysis, and they have inherently low MAPT transcript levels, our results imply that iPSC‐microglia do not express readily detectable levels of tau, in contrast to a recent publication.^79^ Tau is expressed at high levels in neurons, where it binds to and stabilizes microtubules, creating a relatively stable cellular architecture for maintaining a lifelong synaptic network. Microglia, meanwhile, constantly remodel their Cytoskeleton, as their ramifications change over the scale of seconds/minutes, so endogenous tau expression is less likely to be required by microglia.

Our analyses show that monomeric exogenous tau is effectively removed/degraded following uptake by iMGL, while fibrillar tau is degraded inefficiently. Since cells burdened with excess internalized tau fibrils may need to engage other mechanisms of disposal, we looked at routes of tau secretion following uptake. ELISA showed that iMGL release undegraded tau into the supernatant, and that within the supernatant tau could be associated with EVs. While mouse studies^34^, ^36^, ^37^ have implicated microglia‐derived EVs in the spread of tau, we have extended this to show by cryo‐ET that tau fibrils are found packaged within the cytosol of EVs. We have explored whether they might be other fibrillar proteins, for example, microtubules^104^ or actin,^105^ but we did not see changes in the abundance of these or other likely fibrillar proteins in the EVs, and the crossover distance and approximate diameter is compatible with what has been previously demonstrated for heparin‐induced recombinant 2N4R tau fibrils.^85^ We have also quantified several fibril parameters that can help us understand their seeding capacity. Tau fibrils in EVs were approximately 1/10 of the mean length of the input tau “meal,” implying partial digestion and/or size sorting for inclusion within EVs by iMGL and the potential for production of more seeding‐competent fibril ends than the input fibrils. Importantly, by using recombinant tau, we can be confident that the tau‐laden EVs have been generated de novo by iMGL, as opposed to being residual EVs carried over from brain tau preps. While we found that most of the released tau was free in the CM, with EV‐tau comprising a relatively small fraction, the lipidic and protein composition of EVs may directly contribute to tau bioavailability, stability, and uptake in recipient cells,^106^ so further studies could explore uptake mechanisms of these EVs by target cells. For example, LRP1 mediates internalization of enveloped bunyaviruses,^107^ and our EV proteomics dataset includes potential LRP1 ligands (notably APOE, C1q, TIMP1, and A2M), supporting a hypothesis that LRP1 could contribute to EV uptake in recipient cells. Finally, a recent study^23^ explored the structure of tau fibrils within AD brain‐derived EVs by cryo‐EM and observed similar packaging of fibrils into EVs. In that study, it was not possible to attribute EVs to specific cell‐type origins, but the fact that we see very similar structures from iMGL‐EVs argues that in AD brains, microglia are likely contributing seeding‐competent tau‐fibril‐laden EVs to the EV pool.

In summary, using human iPSC‐microglia, coupled with ‐omics and advanced imaging methodologies, we have moved forward from previous rodent studies, providing a detailed subcellular understanding of how tau is handled by human microglia (Figure 8). We have shown that tau uptake is mediated by LRP1 and HSPGs in human microglia, that fibrillar tau shifts microglia to chemokine/interferon response subtypes, is resistant to microglial degradation, can be phosphorylated by microglia, and can be released in seedable form. Demonstration of the structure of intact fibrils within microglia EVs by cryo‐EM advances our understanding of how microglia package and dispose of aggregated proteins and ultimately contribute to pathological tau propagation in dementias.

## AUTHOR CONTRIBUTIONS

*Conceptualization*: M.K.K., A.D.S‐B., A.H., K.S.K., W.S.J., and S.A.C. *Methodology*: M.K.K., A.D.S‐B., A.H., Z.B., E.J, C.E.M., D.P.O., I.V., R.F., S.K., K.A.X.C., W.A.M., W.S.J., and S.A.C. *Software*: S.J.W. *Formal analysis*: A.H., S.J.W., and D.P.O. *Investigation*: M.K.K., A.D.S‐B., A.H., Z.B., D.P.O, I.V., S.S.H., E.J., C.E.M., T.R.S.M‐P., R.M., A.S., and M.A.M. *Data curation*: S.J.W. and D.P.O. *Writing – original draft*: M.K.K., A.D.S‐B., A.H., S.J.W., and S.A.C. *Writing – review and editing*: M.K.K., A.D.S‐B., A.H., S.J.W., Z.B., E.J, C.E.M., D.P.O., W.A.M., K.S.K., W.S.J., and S.A.C. *Visualization*: M.K.K., A.D.S‐B., A.H., S.J.W., and D.P.O. *Supervision*: M.V., K.S.K. T.A.D., W.S.J., and S.A.C. *Project administration*: S.A.C. *Funding acquisition*: T.A.D., K.S.K., W.S.J., and S.A.C.

## CONFLICT OF INTEREST STATEMENT

W.A.M. is an academic founder, shareholder, and scientific advisor for TRIMTECH Therapeutics. T.A.D. is an employee and minor shareholder of Eli Lilly and Company. A.H. was supported by a research grant to S.A.C. from Eli Lilly and Company. The other authors have no conflicts to disclose. Author disclosures are available in the supporting information.

## CONSENT STATEMENT

All authors have read the manuscript and agree to the publication of this manuscript.

## CODE AVAILABILITY

All code for RNAseq is available at https://github.com/S‐Washer/Karabova_2025_Tracking_tau_and_cellular_responses_in_microglia_RNAseq/tree/main

## Supporting information



Supporting Information

Supporting Information

Supporting Information

Supporting Information

Supporting Information

Supporting Information

Supporting Information

Supporting Information

Supporting Information

Supporting Information

Supporting Information

Supporting Information

Supporting Information

## Data Availability

Raw RNA‐seq .fastq, processed kallisto abundance.tsv, and abundance.h5 files have been deposited in Gene Expression Omnibus (GSE291195). The mass spectrometry data have been deposited in ProteomeXchange (PRIDE database).
