# Supplementary material for: Tracking tau and cellular responses in human iPSC‐microglia: from uptake to seedable secretion, including in extracellular vesicles
Source: Alzheimers Dement. 2026 Apr 6;22(4):e71337. doi: 10.1002/alz.71337 (PMC13053939; doi:10.1002/alz.71337)
Supplement: Supplementary file 10 — Supporting Information [file ALZ-22-e71337-s002.pdf]

Supplementary Video 1. 3D reconstruction (tomogram) of internalised Dylight 488-labelled rTauF shown in Fig. 5C.
