# Supplementary material for: Tracking tau and cellular responses in human iPSC‐microglia: from uptake to seedable secretion, including in extracellular vesicles
Source: Alzheimers Dement. 2026 Apr 6;22(4):e71337. doi: 10.1002/alz.71337 (PMC13053939; doi:10.1002/alz.71337)
Supplement: Supplementary file 12 — Supporting Information [file ALZ-22-e71337-s007.pdf]

Tracking tau and cellular responses in human iPSC-microglia: from uptake to seedable secretion, including in extracellular vesicles

Maria Kreger Karabova[1], Anna del Ser-Badia[1], Anne Hedegaard[1], Sam J. Washer[1], Zeynep Baykam[1], Darragh P. O'Brien[2], Iolanda Vendrell[2], Svenja S. Hester [2], Roman Fischer [2], Errin Johnson[3], Charlotte E. Melia[3], Teige R.S. Matthews-Palmer[4,5], Rishi Matadeen[4], Alessia Santambrogio[6], Michael A. Metrick II[6], Michele Vendruscolo[6], Sophie Keeling[7], Kimberly Ai Xian Cheam[7], William A. McEwan[7], Kenneth S. Kosik[8], Theresa A. Day[9], William S. James[1], Sally A. Cowley[1]*

Supplementary information

Supplementary Methods

Human recombinant 2N4R tau protein expression and purification

pETM11-Sumo3-TauWT2N4R plasmid was generated in-house through subcloning Tau protein sequence from pNIC28-Bsa4_6xHis-tag_TauWT 2N4R vector, a gift from Donatella Di Rienzo, Oxford Drug Discovery Institute, into pETM11-Sumo3-eGFP plasmid (EMBL) (Kreger Karabova, M. (2023). Investigating the role of human microglia in tau pathogenesis [PhD thesis]. University of Oxford https://ora.ox.ac.uk/objects/uuid:a9337b8a-d4bd-4bc1-8284-6b803c5e9f0a). A total of 15 ng of plasmid DNA with confirmed correct sequence were transformed into BL21(DE3) *E. coli* and glycerol stock made. Glycerol stocks were used to inoculate 40 mL LB broth with 50 µg/mL kanamycin. Starter cultures were grown overnight at 37°C with 180 rpm agitation. The overnight culture was further inoculated to 1 L of Terrific Broth (TB) broth supplemented with 50 µg/mL kanamycin and 1x TB salts and returned to the shaking incubator at 37 °C with 225 rpm agitation. At OD_{600} = 0.5-0.7, the cells were induced with 0.5 mM isopropyl β-D-1-thiogalactopyranoside (IPTG) and left to grow for 1 h at 37°C and 225 rpm. Cells were then incubated overnight at 25°C with 200 rpm agitation. Cell pellets were harvested by centrifugation at 5,000 xg for 30 min at 4°C using the Beckman Coulter Avanti JXN-26 centrifuge (JLA-8.1000 rotor). Pellets were resuspended in 25 mL ice-cold lysis buffer per 1 L of pelleted culture and stored at -80°C until required.

On the day of the purification, bacterial cells were thawed, lysed using EmulsiFlex-C5 French Press homogeniser operating at 15 kpsi, and centrifuged at 4,500 xg for 1 h. An additional step of 10 min of direct heating in a 70°C water bath, followed by 4,500 xg centrifugation for 1 h at 4°C, was used for the large-scale purification of SUMO-tagged tau. Pellets were discarded and supernatant passed through a 0.45 µm filter.

Samples were kept on ice throughout the entire purification procedure except during the cell heating step.

Purification steps were done using ÄKTA Pure 25M. As all steps were carried out at 4°C, buffer pH was adjusted at 4°C and following sterile-filtering and degassing, were kept at 4°C. The crude extract was applied to HisTrap column pre-equilibrated with 5 column volumes (CV) of buffer A1. The column was then washed with 50 CV buffer A2, which contains 0.1% Triton X-114[1] and it is a critical step for endotoxin removal from the preparation, followed by 20 CV buffer A1. Protein was eluted from the column with a linear gradient of buffer B. Overnight dialysis at 4°C in SnakeSkinT^{M} Dialysis Tubing in the presence of Sentrin-specific protease 2 (SENP2) protease (1:100 protease:protein ratio), against 4 L of dialysis buffer was used to remove imidazole and cleave the 6xHis-SUMO tag.

SENP2 protease, purified in-house from *E. coli,* was a generous gift from Tanmay Bharat lab. The dialysed and cleaved product was re-applied to a HisTrap column pre-equilibrated with 5 CV of buffer C. Tag-less tau was eluted from the column with a linear gradient of buffer D to isolate the tag-less tau. Fractions containing cleaved tau were pooled and further purified using the CaptoS column pre-equilibrated with 5 CV of buffer E. The column was washed with 6 CV of buffer E and eluted with linear gradient of buffer F. Purest tau-containing fractions were concentrated to 2 mL using 10K MWCO Pierce Protein concentrator and filtered through a 0.22 µm cut-off filter. Residual endotoxin was removed from the final product using Pierce High-Capacity Endotoxin Removal Spin Column, as per manufacturer’s instructions. Endotoxin levels were quantified with Pierce Limulus Amoebocyte Lysate (LAL) Chromogenic Endotoxin Quantitation Kit. The concentration of endotoxin-free 2N4R tau monomer was measured using Pierce BCA Protein Assay Kit - Reducing Agent Compatible due to the presence of β-mercaptoethanol in the elution buffer. Sample was aliquoted into Protein LoBind® tubes, and stored at -80°C.

If columns were re-used multiple times, a column cleaning procedure (1 h soak with 1 M NaOH followed by 10 CV ddH2O and 10 CV buffer A1 or buffer E washes) would precede sample application. Eluted protein fractions were analysed with SDS-PAGE gel electrophoresis. A total volume of 15 µL eluate was mixed with NuPAGE™ 4x LDS sample buffer and boiled at 70°C for 10 min. Samples were loaded on Novex™ WedgeWell™ 8-16%, Tris-Glycine pre-cast mini gels, and ran in Novex™ Tris-Glycine SDS Running Buffer at 200 V for 45 min. A 1 h RT gel incubation with SimplyBlue™ Safe Stain was used for protein detection.

All resources and buffer compositions are described in Supplementary Tables 5 and 6.

Enrichment of tau PHFs from human AD brains

Brain-derived tau (hTau) was enriched from Alzheimer’s patient brains and QCed by E.M and Western blot and toxicity assessed (Supp. Fig 2A-C,E). The treatment dose of 0.25 µg/mL was chosen as this conferred no toxicity to iMGL after 24 h incubation.

Samples of brain material were obtained from the London Neurodegenerative Diseases Brain Bank at King's College London and processed at Eli Lilly and Company's facility in Indianapolis, IN, US. Seeding-competent tau was extracted as previously described[2] with modifications noted below from samples of frontal and temporal cortex, obtained from individuals diagnosed with AD (BNE/Braak stage 5-6), with an average age of 80.2 ± 2.3 years (range: 63-98 years). Tissues were dissected after an average postmortem delay of 30.6 ± 4.2 h (range: 10-66 h). Numerous brain tissue pieces from each AD patient tissue slab were preliminarily screened using a proprietary AT8 tau Alphascreen assay developed by Lilly (AT8 and DA9 antibodies obtained from Peter Davies). Each preparation utilized tissue pieces with high, medium, and low AT8 tau signal with a cut-off of > 0.25 ug/mL AT8 tau signal. Percentages of high, medium, and low AT8 tau signal tissue pieces were maintained across preparations to ensure consistency.

A total of 100 g of tissue per donor was processed by repeated homogenisation and filtration as follows: Frozen postmortem tissue samples were shipped on dry ice and stored at -80˚C until needed. Tissues were defrosted on ice and broken into smaller pieces before being added to cold homogenisation buffer (4 mL/1 g of tissue, sterile PBS (#14190-144, Gibco) containing Complete protease inhibitor cocktail (#11836145001, Roche)). Homogenisation was achieved using an Ultra Turrax (IKA T25, 20,000 rpm, 10min), after which the homogenate was centrifuged at 10,000 rpm for 10 min at 4°C. The supernatant was carefully removed and filtered through a 2-ply tissue into a beaker on ice, upon which the pellet was resuspended again in 400 mL homogenisation buffer and the homogenisation and filtration process repeated for a total of three times.

The resulting homogenate (~1000 mL) was then split between 500 mL centrifuge bottles (#431123, Corning) and appropriate volumes of 30% sarkosyl solution (N-Lauroylsarcosine sodium salt 30% aqueous solution, #61747, Sigma-Aldrich-Aldrich) were added for a final concentration of 1% sarkosyl. Following a 1 h incubation at room temperature on a flat rotating shaker at a medium speed, samples were centrifuged for 1 h at 45,000 rpm at 4°C (Optima XPN-80 ultracentrifuge (45Ti rotor), Beckman Coulter 65 mL ultracentrifuge tubes #355655). The extracted pellet was washed once by carefully adding and removing 1 mL homogenisation buffer, before being resuspended in 50 µL/initial gram of sample (5 mL/100 g tissue), of PBS containing 0.5x EDTA-free complete protease inhibitor cocktail (#11873580001, Roche) and transferred to a 15 mL falcon tube for sonication. Each sample was sonicated (Fisherbrand™ Model 120 Sonic Dismembrator with CL-18 probe) as follows until visible clumps were gone: 10 x 1 sec pulses (1 sec on, 1 sec off) at 40% amplitude ~four times, and then again ~twice after combining the samples. For this study, three separate preparations comprising a total of 17 AD patient tissue samples (12 females and 5 males at 63-90 years old) were pooled. The total-tau concentration of the pooled preparation was assessed by a proprietary Alphascreen assay developed by Lilly (DA9 and TG5 antibodies obtained from Peter Davies) in the presence of guanidine or PBS, using monomeric tau and PHF as standard curves and resulted in 78.7 µg/mL.

A rat cortical neuron seeding cell assay was performed to determine the optimal seeding concentration for the pooled tau seed preparation. Briefly, the pooled tau

seed preparation was diluted to 2 µg/mL total tau in cell culture medium, sonicated (60 x 1 sec pulses at 20% amplitude), and filtered through a 0.22 µm filter into a deep-well plate. The resulting filtrate was serially diluted two-fold for a nine-point concentration-response curve. Enough media was removed from the rat cortical neuron cells at DIV8 (plated at 30K cells/well at DIV0) to leave 50 µL in the well, and 50 µL of cell medium or serially diluted pooled tau seed was added for a final concentration range of 0.0039-1 µg/mL total tau pooled tau seed. Half of the treated cell medium was removed and replaced with fresh medium on DIV16. On DIV22, all the medium was removed and the cells washed 3x with 100 µL PBS. After the final wash, the PBS was removed and 100 µL of 100% methanol (chilled at -20˚C) was added for 15 min at 4˚C to remove soluble proteins and fix the cells. Cells were washed 3x with 100 µL PBS and a final 100 µL PBS was added for storage at 4˚C prior to immunostaining and imaging.

To determine the amount of tau seeding, PBS was removed from the fixed cells and 100 µL of Odyssey blocking buffer with 0.1% Triton was added and incubated at room temperature for 60 min. The blocking buffer was removed and 100 µL of primary antibodies diluted in Odyssey blocking buffer with 0.1% Triton was added and incubated overnight at 4˚C while shaking at a slow speed. Cells were stained for rodent total tau using the T49 tau antibody at a 1:2000 dilution and co-stained with a NeuN antibody at a 1:1000 dilution to detect neurons. Primary antibodies were removed, and cells were washed 3x with 100 µL PBS, followed by incubation with 100 µL of appropriate secondary antibodies and nuclei-stain Hoechst 33342 at a 1:1000 dilution in Odyssey blocking buffer with 0.1% Triton for 1 h at room temperature. Secondary antibodies were removed and cells were washed 3x with 100 µL PBS and a final 100 µL PBS was added for storage in the dark at 4˚C until imaged using an Operetta instrument and data analysis software to determine the number of T49 tau inclusions per number of nuclei at each seed concentration. The resulting average EC80 seed concentration (0.1 µg/mL) determined from two separate tests was used as the optimal seeding concentration for additional seeding studies.

Proteomics

Sample Preparation

iMGL were lysed in RIPA buffer supplemented with 5% SDS and protease and phosphatase inhibitors (Roche). Proteins secreted to the conditioned medium (CM) were precipitated by overnight incubation with 6% trichloroacetic acid (TCA) at 4°C in agitation. CM was then centrifuged at 10,000 xg for 10 min at 4°C, the supernatant was discarded, and the pellet was air-dried for 10 min and lysed in RIPA with 5% SDS. EVs were isolated as described above and lysed on ice for 10 min with 10x RIPA supplemented with 5% SDS and protease and phosphatase inhibitors.

Proteomic Digestion

Approximately 300 µg of protein material was digested using an S-Trap™ sample processing procedure in a 96 well format according to the manufacturer's instruction[3]. Briefly, proteins were reduced with 20 mM DTT for 30 min at RT and alkylated with 40

mM of iodoacetamide, also for 30 min at RT and in the dark. Proteins were acidified to reach a final concentration of 1.2% phosphoric acid. Samples were then subjected to a six-fold dilution with 90% methanol/100 mM tetraethylammonium bromide (TEAB). Diluted protein material was then loaded and washed on an S-Trap™ plate. Each protein sample was digested with 6 µg of trypsin overnight at 37°C, achieving a final protein:enzyme ratio of 50:1. Tryptic peptides underwent sequential elution from the column, with a final elution buffer of 50% acetonitrile (ACN). EV and conditioned media samples were subjected to an identical procedure, except that in the starting volume of 50 µL, the concentration/amount of material was unknown.

Phosphopeptide Enrichment - Bravo Enrichment

Approximately 10% of each tryptic peptide digest was kept aside to perform a total proteome analysis. The remaining sample was desalted using SOLA HRP cartridges and adapted with minor changes to manufacturer's protocol for phosphopeptide enrichment. Briefly, cartridges were first conditioned with solvent B (70% ACN, 0.1% trifluoroacetic acid (TFA)) and then washed with solvent A (0.1% TFA in H2O). Peptides were loaded in a 1:1 ratio (sample/solvent A). Bound peptides were then washed with solvent A, followed by elution with 1M glycolic acid in 50% ACN, 5% TFA. Phosphopeptide enrichment was performed using the pre-defined Agilent Bravo AssayMap liquid handler workflow for TiO2 cartridges (AssayMap 5 µL Titanium Dioxide TiO2 cartridges, Catalogue Number G5496-60016, Agilent). The prime and syringe wash solution consisted of 50% ACN, 5% NH3 in H2O and the equilibration and cartridge wash solution was 50% ACN, 2% TFA, 1 M glycolic acid in H20. Phosphopeptides were eluted in 15% ACN, 5% NH3. Samples were loaded onto the cartridges at 2.5 µL/min. Eluted phospho-enriched peptides were dried down and resuspended in 5% DMSO and 5% formic acid (FA).

Manual Tip Enrichment for Purified Tau Inputs

Briefly, 250 µg of dried samples were re-suspended in 50 µL loading buffer (80% ACN, 5% TFA and 1M glycolic acid). TiO2 columns (Glycogen) were first spun down for 30 sec at 2,000 x g with the caps on to ensure all material was at the bottom of the tip. Columns were then washed once with 50 µL of elution buffer (5% ammonium hydroxide) at 1,500 x g for 2 min, followed by three washes of 65 µL loading buffer at 2,000 x g for 5 min. Following this, the LoBind® Eppendorf tubes were replaced with fresh ones. Samples were then loaded in 20 µL volumes and at 2,000 x g for 5 min until all material was bound to the column. Columns were washed twice with 65 µL loading buffer, once with 65 µL wash buffer 1 (80% ACN, 0.2% TFA), and then finally, two washes of 65 µL wash buffer 2 (20% ACN). All spins were performed at 2,000 x g for 5 min. After changing to fresh LoBind® Eppendorf tubes, 20 µL of 20% FA was added to each tube. Phosphopeptides were eluted by two washes of 20 µL elution buffer into the same tube. To recover residual eluate, a spin at 2,000 x g for 2 min was performed.

LC-MS/MS of total proteome and phosphoproteome samples

Peptides were chromatographically separated on a 15 cm x 150 µm x 1.5 µm analytical column (EV1137, Evosep) using the 30 samples per day (spd) gradient method of an Evosep One LC system. Eluted peptides were directed for mass spectrometry (MS) analysis on a timsTOF Pro mass spectrometer (Bruker). Phosphopeptide MS data were acquired in ddaPASEF mode. The ion mobility window was 0.60-1.60 Vs/cm2, with an accumulation and ramp time of 100 ms. The mass range of MS and MS/MS scans was *m/z* 100 – 1,700. MS/MS spectra were acquired in 10 PASEF ramps with a 4-frame overlap, giving a cycle time of 1.17 sec. Ions were selected for PASEF MS/MS if they met an intensity threshold of 2,500 and were sampled multiple times until a target intensity of 20,000 was reached. A polygon filter was used to exclude singly charged ions from MS/MS selection. Ions selected for fragmentation were isolated by the quadrupole and fragmented using an ion mobility-dependent collision energy that increased nonlinearly over the ion mobility range as follows: a collision energy (eV) of 20 at a 1/K0 of 0.60 Vs/cm2, 22 at 0.70, 25 at 0.75, 30 at 0.80, 35 at 0.85, 40 at 0.90, 45 at 0.95, 50 at 1.00, 55 at 1.10, 60 at 1.20, 65 at 1.30, 70 at 1.40, 75 at 1.50, and 80 at 1.60. A dynamic exclusion time of 24 sec was used. Total proteome MS data were acquired in diaPASEF mode using 16 diaPASEF scans per TIMS-MS scan with an accumulation and ramp time of 100 msec, for a total cycle time of 1.80 sec. The ion mobility range was set to 0.6-1.6 Vs/cm2. Each mass window isolated was *m/z* 26 wide, ranging from *m/z* 400-1200 with an ion mobility-dependent collision energy that increased linearly from 20 eV to 59 eV between 0.6-1.6 Vs/cm2.

LC-MS/MS of EV and conditioned media samples

Samples were analysed by LC-MS/MS using the Vanquish Neo UHPLC connected to Thermo Orbitrap Ascend mass spectrometer equipped with a high-field asymmetric ion mobility spectrometry (FAIMS) Pro Duo interface (all Thermo Fisher Scientific). The Vanquish Neo was operated in “Trap and Elute” mode using a PepMap Neo trap (5 µm, 300 µm x 5 mm; Thermo Fisher) and EASY-SPRAY PepMapNeo column (50 cm x 75 µm, 1500 bar; Thermo Fisher). Tryptic peptides were trapped and separated over a 75 min gradient, going from 2 to 18% B (0.1% FA in 100% ACN) in 40 min, to 35% in 20 min, up to 99% B in 1 min and then staying at 99% for 14 min. The flow rate was maintained at 300 nL/min throughout the gradient. The FAIMS Pro™ interface was operated in standard resolution with carrier gas flow rate of 3.8 L/min and compensation voltage set to -45. MS data were acquired in Data Independent Acquisition (DIA) mode, with minor changes from our previously described method[4-6]. Briefly, MS1 scans were collected in the Orbitrap mass analyser at a resolving power of 45K at *m/z* 200 over a range of *m/z* 350 – 1650. The MS1 normalised AGC was set at 125% (5e5 ions) with a maximum injection time of 91 msec and a RF lens at 30%. DIA MS2 scans were then acquired using the tMSn scan function at an Orbitrap resolution of 30K over 40 scan windows of variable width, with a normalised AGC target of 1000%, maximum injection time set to auto and a 30 % collision energy.

Data Analysis

MS raw files were searched against the Homo sapiens database downloaded from UniProtKB on November 7th 2023. Total proteome and phosphoproteome Data Dependant Acquisition (DDA) data were searched using Fragpipe (v22.0), while EV and conditioned media DIA data was searched using DIA-NN Software (v1.9) in library-free mode. Carbamidomethyl of Cys residues was set as a fixed modification, while oxidation of Met and N-term acetylation were set as variable modifications. Phosphorylation of Ser, Thr, and Tyr was set as a variable modification, where relevant. A maximum of 1 missed cleavage was allowed and the matching between runs function was turned off. Search results were analysed and visualised in Perseus (v. 2.0.10.0) by means of Principal Component Analysis (PCA), Volcano and Scatter plots. Dysregulated proteins and phosphopeptides across conditions were subjected to Gene Ontology and pathway enrichment analysis in STRINGdb (https://string-db.org).

Supplementary Tables

Supplementary Table 1: Endotoxin removal from recombinant tau in published studies

**Publication**	**Recombinant tau source**	**Endotoxin removal methods used**	**Endotoxin detection test**	**Test results provided**
Asai et al., 2015	rPeptide (T-1001-1), *E. coli* purification	N	N	N
Zhu et al., 2022	AnaSpec (AS-55556), *E. coli* purification	N	N	N
Udeochu et al., 2023	non-disclosed	N	N	N
Perea et al., 2022	ClearColi purification	Y	Y	N/A
Funk et al., 2015	*E. coli* purification	N	N	N
Ising et al., 2019	*E. coli* purification	N	Y	N
Stancu et al., 2019	*E. coli* purification	N	N	N
Pampuscenko et al., 2020	*E. coli* purification	N	Y	Y (0.001-0.007% w/w LPS/tau)
Zilkova et al., 2020	*E. coli* purification	Y	N	N

Das et al., 2020	*E. coli* purification	N	N	N
Pampuscenko et al., 2021	*E. coli* purification	N	Y	Y (0.001-0.007% w/w LPS/tau)
Jin et al., 2021	*E. coli* purification	N	N	N
Wang et al., 2022	*E. coli* purification	N	Y	Y (<1 EU/mL in working concentration)
Chinnathambi and Das, 2023	*E. coli* purification	N	N	N
Falkon et al., 2024	StressMarq Biosciences (SPR-327, SPR-329) E. coli BL21	Y	Y	Y (<0.5 EU/mL at 2 mg/mL)
Bolos et al., 2017	*E. coli* purification	N	N	N
Chidambaram et al., 2024 (bioRxiv)	*E. coli* BL21 purification	N	N	N
Crotti et al., 2019	*E. coli* BL21 Star (DE3) pLysS purification	N	N	N
Funk, 2024	*E. coli* purification	N	N	N
Kovac et al., 2011	*E. coli* purification	N	Y	Y (<0.001 EU/mL)

Supplementary Table 2: Cell lines used and donor information

ID #	Cell line	Donor age	Donor sex	Diagnosis	APOE isoform	LRRK2 genotype	Symbol
1	SFC840-03-03	65-69	F	Healthy control	E3/E3	WT/WT	circle
2	SFC841-03-01	35-39	M	Healthy control	E3/E3	WT/WT	square
3	SFC856-03-04	75-79	F	Healthy control	E3/E4	WT/WT	triangle
4	KOLF2.1S	55-59	M	Healthy control	E3/E3	WT/WT	diamond
5	SFC840-03-03 D10	65-69	F	CRISPR/Cas9 edited	E3/E3	-/-	half black circle
6	SFC832-03-06	75-79	F	G2019S PD	E3/E4	G2019S/WT	dotted square

7	SFC833-03-05	80-84	M	G2019S PD	E2/E3	G2019S/WT	dotted diamond
8	SFC855-03-06	55-59	M	G2019S PD	E2/E3	G2019S/WT	dotted hexagon

Supplementary Table 3: Overview of cell lines used in figures

**Main figures**	**Panel**	**Cell line ID**	**Cell type**
Fig. 1	B	1	iMGL
	C	1, 2, 3, 4	iMGL
	D	1, 2, 3	iMGL
	E	1, 3	iMGL
Fig. 2	B-F	3	iMGL
Fig. 3	A-E	3	iMGL
Fig. 4	B	1, 2, 3	iMGL
	C	3	iMGL
Fig. 5	B-D	1, 3, 4	iMGL
Fig. 6	A	3, 4	iMGL
	B	3, 4	iMGL
	D-G	3	iMGL
Fig. 7	B	1, 3, 4	iMGL
	C	3	iMGL
	D, E	1, 3	iMGL, iNeurons
**Supplementary figures**	**Panel**	**Cell line ID**	**Cell type**
Supp.Fig.1	G		HEK293
Supp. Fig.2	D	1, 3, 4	iMac, iMGL
	E	4	iMGL
Supp.Fig.3	A	1	iMGL

	B	1	iMac
	C	1, 2, 3, 4	iMac
Supp. Fig. 4	A	1, 2, 3	iMac
	B	1, 3, 4	iMac
	C	1, 3	iMGL
Supp. Fig. 5	A	1, 3, 5, 6, 7, 8	iMac
	B	1, 3, 4, 5, 6, 7, 8	iMac
	C	1, 4, 5, 6, 8	iMac
Supp. Fig. 6	B	1, 2, 3	iMGL
	C, D, E	3	iMGL
Supp. Fig. 7	D	3	iMGL
Supp. Fig. 8	B	1, 2, 3, 4	iMGL
	C	2	iMGL
Supp. Fig. 9	A-D	1, 3, 4	iMGL
Supp.Fig. 10	A-C	1, 3, 4	iMGL
Supp. Fig. 11	A	1, 2, 3, 4	iMGL
	B	2, 3	iMGL
	C	1, 3, 4	iMGL
	D-F	1, 3, 4	iMGL
Supp. Fig. 12	B	3	iMGL
	C	1	iMGL
	D	1, 3	iMGL
Supp. Fig. 13	C	4	iMGL
	D	1, 3, 4	iMGL

Supplementary Table 4: iMac, iMGL and iNeuron differentiation media

**Medium**	**Reagent**	**Supplier**	**Catalogue number**	**Final concentration**

OXE8	Advanced DMEM/F12	Thermo Fisher	12634010	1x
	GlutaMAX	Thermo Fisher	35050-038	1x
	Heparin solution 0.2%	StemCell Technologies	07980	100 ng/mL
	Ascorbic Acid	Sigma-Aldrich	A8960	0.22 mM
	HEPES	Thermo Fisher	15630080	15 mM
	FGF-2	R&D Systems	4114-TC-01M	100 ng/mL
	TFG-β	Peprotech	AF-100-21C	2 ng/mL
Embryoid body (EB) medium	OXE8	-	-	1x
	BMP4	Peprotech	PHC9534	50 ng/mL
	VEGF	Peprotech	PHC9394	50 ng/mL
	SCF	Miltenyi Biotec	130-096-695	20 ng/mL
XVIVO precursor medium	X-VIVO 15	Lonza	BE02-060F	1x
	GlutaMAX	Thermo Fisher	35050-038	1x
	2-mercapto-ethanol	Gibco	31350-010	1%
	IL-3	Invitrogen	PHC0033	25 ng/mL
	M-CSF	Invitrogen	PHC9501	50 ng/mL
	Pen/Strep	Thermo Fisher	15140-122	1%
OXM macrophage medium	Advanced DMEM/F12	Thermo Fisher	12634010	1x
	GlutaMAX	Thermo Fisher	35050-038	1x
	Human recombinant insulin solution	Sigma-Aldrich	19278-5ML	5 µg/mL
	HEPES 1 M pH 7.4	Thermo Fisher	15630080	15 mM
	M-CSF	Invitrogen	PHC9501	50 ng/mL
ITMG microglia medium	Advanced DMEM/F12	Thermo Fisher	12634010	1x
	GlutaMAX	Thermo Fisher	35050-038	1x
	M-CSF	Invitrogen	PHC9501	25 ng/mL
	GM-CSF	Invitrogen	PHC2013	10 ng/mL
	IL-34	Peprotech	200-34	100 ng/mL
	TGFβ1	Peprotech	100-21C	50 ng/mL
iNeuron medium	Neurobasal Plus	Thermo Fisher	A3582901	1x
	GlutaMAX	Thermo Fisher	35050-038	1x
	BDNF	Peprotech	450-02-10	10 ng/mL
	B27 Plus	Thermo Fisher	A35828-01	1x
	L-Ascorbic Acid	Sigma-Aldrich	A0278	0.2 µM
	NT3	Peprotech	450-03-10	10 ng/mL
	Geltrex	Invitrogen	A1413302	3.3 µL/mL
	Doxycycline	Sigma-Aldrich	D9891	2 µg/mL

Supplementary Table 5: List of reagents for recombinant tau expression and purification

Reagent or resource	Supplier	Catalogue number
**PLASMIDS**		
pNIC28-Bsa4_6xHis-tag_TauWT 2N4R	Donatella Di Rienzo	
pETM11-Sumo3-eGFP	EMBL	pETM11-Sumo3-eGFP
pETM11-Sumo3-TauWT2N4R	In house	
**BACTERIAL STRAIN**		
BL21(DE3) *E. coli*	Thermo Fisher	EC0114
**ENZYMES**		
SUMO-Specific Protease 2 (SENP2)	Tanmay Bharat	
SUMO-Specific Protease 2 (SENP2)	Leadgene	LDG0015RG
**CHEMICALS/REAGENTS**		
LB broth		
Kanamycin	Sigma	K4000
Potassium phosphate monobasic	Sigma	P5379-500G
Potassium phosphate dibasic	Sigma	P8281-500G
Glycerol	Sigma	G6279
Sodium chloride	Sigma	S7653-1KG
Yeast extract	Sigma	Y1625-1KG
Tryptone	Sigma	T7293-1KG
IPTG	Sigma	I5502-5G
HEPES	Sigma	H3375-500G
Triton X-114	Sigma	X114
Imidazole	Millipore	288-32-4
β-mercaptoethanol	MP Biomedicals	190242
SimplyBlue™ SafeStain	Thermo Fisher	46-5044
Bond-breaker™ TCEP Solution	Thermo Fisher	77720
DTT	Sigma Aldrich	43816
DNase	Invitrogen	18047-019
Heparin	Sigma Aldrich	H3393
WF1 for cell culture	Gibco	A12873-01
Pierce Protease Inhibitor XL Capsules, EDTA-free	Thermo Fisher	A37989
**OTHERS**		
Novex WedgeWell 8-16% Tris-Glycine Gel 1mmx15 wells	Invitrogen	XP08165BOX
NuPAGE™ 4x LDS sample buffer	Invitrogen	NP0007
Precision Plus Protein Dual Colour Standards	Bio-Rad	1610374

Tris-Glycine SDS Running buffer	Thermo Fisher	LC2675
0.45 µm filters	Starlab	E4780-1456
0.22 µm filters	Starlab	E4780-1226
HisTrap™ HP	Cytiva	17524802
HiTrap™ Capto™S	Cytiva	17544123
SnakeSkin™ Dyalisis Tubing	Thermo Fisher	68035
Pierce Protein Concentrator PES, 10K MWCO, 5-20 ml	Thermo Fisher	88528
Pierce High Capacity Endotoxin Removal Spin Column, 0.5 ml	Thermo Fisher	88274
Pierce LAL Chromogenic Endotoxin Quant kit	Thermo Fisher	A39553
Pierce BCA Protein Assay Kit - Reducing Agent Compatible	Thermo Fisher	23250
**PLASTICWARE**		
Cuvettes	VWR	634-0676
1 L Polypropylene Bottle Assembly	Beckman Coulter	C31597
75mm bottle top filter - 500 ml	Nalgene filtration products	291-4520
15 mL Protein LoBind Tube	Eppendorf	0030122208
50 mL Protein LoBind Tube	Eppendorf	0030122240
0.5 mL Protein LoBind Tube	Eppendorf	022431064
1.5 mL Protein LoBind Tube	Eppendorf	022431081
**EQUIPMENT**		
Innova®44 Incubator Shaker	New Brunswick Scientific	
SpectraMax M5 microplate reader	Molecular Devices	
Avanti JXN-26	Beckman Coulter	
JLA-8.1000 Fixed-Angle Aluminium rotor	Beckman Coulter	363688
EmulsiFlex-C5 French Press homogeniser	Avestin	
AKTA Pure 25M	Cytiva	

Supplementary Table 6: Buffer composition for recombinant tau expression and purification

Buffer	Component	Final concentration
Terrific broth (TB)	Tryptone	12 g/L
	Yeast extract	24 g/L
	Glycerol	0.4% v/v

10x TB salt	Potassium phosphate monobasic	0.17 M
	Potassium phosphate dibasic	0.72 M
Lysis (pH 7.6)	HEPES	25 mM
	NaCl	150 mM
	Imidazole	10 mM
	Bond Breaker ™ TCEP	1 mM
	DNase I	1.68 U/mL
	Pierce™ Protease Inhibitor XL Capsules, EDTA-free	1x
HisTrap A1 (pH 7.6)	HEPES	25 mM
	NaCl	150 mM
	Imidazole	10 mM
	Bond Breaker ™ TCEP	1 mM
	Pierce™ Protease Inhibitor XL Capsules, EDTA-free	1:500
HisTrap A2 (pH 7.6)	HEPES	25 mM
	NaCl	150 mM
	Imidazole	10 mM
	Bond Breaker ™ TCEP	1 mM
	Triton™X-114	0.1%
HisTrap B (pH 7.6)	HEPES	25 mM
	NaCl	150 mM
	Imidazole	500 mM
	Bond Breaker ™ TCEP	1 mM
	Pierce™ Protease Inhibitor XL Capsules, EDTA-free	1:500
Dialysis (pH 7.6)	HEPES	25 mM
	NaCl	100 mM
	β-mercaptoethanol	5 mM
HisTrap Rebind C (pH 7.1)	HEPES	25 mM
	NaCl	100 mM
	Imidazole	10 mM
	β-mercaptoethanol	5 mM
HisTrap Rebind D (pH 7.1)	HEPES	25 mM
	NaCl	100 mM
	Imidazole	500 mM
	β-mercaptoethanol	5 mM
CIEX E (pH 7.1)	HEPES	25 mM
	NaCl	100 mM
	β-mercaptoethanol	5 mM
CIEX F (pH 7.1)	HEPES	25 mM
	NaCl	1 M
	β-mercaptoethanol	5 mM

Supplementary Table 7: iMac and iMGL pharmacological and LentiCRISPR treatments

Reagent	Supplier	Catalogue Number	Final concentration
IFNγ	Gibco	PHC0044	100 ng/mL
LPS	Invivogen	Tlrl-eklps	100 ng/mL
sRAP	Ximbio	153996	5-500 nM
Heparin	Sigma-Aldrich	H3393	10 μg/mL
FKN	PeproTech	300-31	5 ng/mL
Leupeptin	Enzo Life Sciences	ALX-260-009-M005	50 μM
Pepstatin A	Enzo Life Sciences	ALX-260-085-M005	50 μM
E64d	Abcam	ab144048	50 μM
MG-132	Sigma-Aldrich	474791	25 μM
**Target**	**gRNA #**	**Sequence**	
LRP1	1	GTCTCGATGCGGTCGTAGA	
LRP1	2	TCTGTACTGGACGGACGAT	
LRP1	3	GCCGAGACCGCTCAATACG	
INTG	1	TTGGGCAGAATGTCTGCCC	
INTG	2	AAGCTCCTCACCATGCCCA	
INTG	3	CAGTTGCCTAACAGGAGCA	
INTG	4	ACCAAGGGTTACCAAGAAG	
INTG	5	CACCTCCCAGTGTCTTGAA	
INTG	6	CAGAGGTCAACCTTGACCC	
INTG	7	CTTATTAGGGATCAAGGGT	
INTG	8	CTAGATCTAGGGTGTGTTG	

Supplementary Table 8: List of antibodies

Antibody	Supplier	Catalogue Number	FACS	ICC	WB	ID
LRP1	Abcam	ab92544	1:100	1:200		
LRP1	Cell Signaling	64099			1:500	
Tau12	Sigma-Aldrich	MAB2241		1:200	1:250	
MAP2	Cell Signalling	8707		1:200		
AT8	Thermo Fisher	MN1020		1:500		
DA9	Peter Davies	N/A			1:1000	
T49	Millipore	MABN827		1:2000		
PHF-1	Peter Davies	N/A			1:1000	
Vinculin	Bio-Rad	MCA465GA			1:2000	
Flotilin-I	Cell Signaling	18634			1:500	
Annexin-V	Cell Signaling	8555			1:500	

CD9	Cell Signaling	13174			1:500	
CD9	Abcam	ab263019			1:500	
Calnexin	Abcam	ab133615			1:2000	
NeuN	Millipore	ABN91		1:1000		
HT7	Invitrogen	MN1000				50 µg
Mouse IgG1	Santa Cruz	sc-3877				50 µg
Donkey anti-Mouse A647	Invitrogen	A31571		1:500		
Donkey anti-Rabbit A568	Invitrogen	A10042		1:500		
Goat anti-Mouse IgG1 647	Invitrogen	A21240		1:500		
Goat anti-Chicken 488	Invitrogen	A32931TR		1:500		
IRDye®800CW Goat anti-Rabbit	LI-COR	926-32211			1:2000	
IRDye®680RD Goat anti-Mouse	LI-COR	926-68072			1:2000	

Supplementary Table 9: List of reagents

Reagent	Supplier	Catalogue Number
mTESR1	StemCell Technologies	85850
Geltrex	Invitrogen	A1413302
EDTA	Invitrogen	15575-020
TrypLE	Gibco	12604013
Aggrewell 800 plates	StemCell Technologies	34815
ROCK inhibitor Y-27632	Abcam	1201029
Poly-L-Ornithine	Sigma-Aldrich	P4957
Laminin	Sigma-Aldrich	L2020
Puromycin	Thermo Fisher	A1113803
AraC	Sigma-Aldrich	C1768
DMEM	Sigma-Aldrich	D6429
FBS	Sigma-Aldrich	F9665
Human IgG	Sigma-Aldrich	I8640-100MG
Sodium Azide	Sigma-Aldrich	08591
OptiMEM	Gibco	31985-062
Lipofectamine2000	Invitrogen	11668027
StemPro Accutase	Gibco	A1110501
PBS	Gibco	14190-144
Complete protease inhibitor cocktail	Roche	11873580001

30% sarkosyl solution	Sigma-Aldrich	61747
Dynabeads® Antibody Coupling kit	Life Technologies	14311D
4% PFA	Thermo Fisher	J61899.AP
Triton-X100	Sigma-Aldrich	X100
Donkey serum	Bio-Rad	C06SB
DAPI	Abcam	ab228954
Hoechst 33342	Invitrogen	H3570
HEPES	Sigma-Aldrich	H0887
300-mesh carbon-coated copper grids	TAAB Laboratories	C267
3nm lacey carbon grids	Agar Scientific	AGS178-4
200-mesh carbon-coated copper grids	TAAB Laboratories	C101
Uranyl acetate	Agar Scientific	R1260A
Glutaraldehyde	Agar Scientific	R1020
PIPES buffer	Sigma-Aldrich	P6757
Glycine	Sigma-Aldrich	G7126
Osmium tetroxide	TAAB Laboratories	O001/1
Potassium ferrocyanide	Acros Organic	223111000
Agar 100-Hard epoxy resin	Agar Scientific	AGR1140
BEEM capsules	Agar Scientific	AGG360-1
Hexamethyldisilazane	Sigma-Aldrich	440191
RIPA buffer	Thermo Fisher	89901
Complete protease inhibitor cocktail	Roche	5892791001
Pierce Phosphatase inhibitor mini tablets	Thermo Fisher	A32957
10x RIPA buffer	Cell Signaling	9806
Pierce™ BCA Protein Assay Kit	Thermo Fisher	23227
NuPAGE™ 4x LDS sample buffer	Invitrogen	NP0007
NuPAGE™ 10x sample reducing agent	Invitrogen	NP0009
DTT	Sigma-Aldrich	43816
Novex™ WedgeWellTM 8-16%, Tris-Glycine pre-cast mini gels	Invitrogen	XP08165BOX
Precision Plus Protein Dual Color Standards	Bio-Rad	161-0374
NovexTM Tris-Glycine SDS Running Buffer	Invitrogen	LC2675
Low-Fluorescence PVDF transfer membrane	Thermo Fisher	22860
Trans-Blot Turbo Transfer Buffer	Bio-Rad	10026938
iBind™ Flex blocking solution	Thermo Fisher	SLF1020
Tween® 20	Sigma-Aldrich	P7949

SDS	Sigma-Aldrich	75746
Invitrogen™ Tau (Total) Human ELISA kit	Thermo Fisher	KHB0041
IL-1β ELISA kit	Thermo Fisher	88-7261-88
Human Uncoated IL-6 ELISA kit	Thermo Fisher	88-7066-88
3.5 mL polycarbonate tubes	Beckman Coulter	349622
13.2 mL ultra-clear polypropylene tubes	Beckman Coulter	344059
Resazurin	Sigma-Aldrich	199303
LDH detection kit	Biolegend	426401
ThT	Sigma-Aldrich	T3516
GuHCl	Merck	G3272
Superdex 75 10/300 column	Cytiva	17517401
Silver stain kit for Mass Spectrometry	Thermo Fisher	24600
Ponceau S stain	Sigma-Aldrich	P3504
Trizma® base	Sigma-Aldrich	T1503

Supplementary References

1. Reichelt P, Schwarz C, Donzeau M. Single step protocol to purify recombinant proteins with low endotoxin contents. *Protein Expression and Purification*. 2006/04/01/ 2006;46(2):483-488. doi:https://doi.org/10.1016/j.pep.2005.09.027
2. Katsikoudi A, Ficulle E, Cavallini A, et al. Quantitative propagation of assembled human Tau from Alzheimer's disease brain in microfluidic neuronal cultures. *Journal of Biological Chemistry*. 2020;295(37):13079-13093. doi:10.1074/jbc.RA120.013325
3. HaileMariam M, Eguez RV, Singh H, et al. S-Trap, an Ultrafast Sample-Preparation Approach for Shotgun Proteomics. *Journal of Proteome Research*. 2018/09/07 2018;17(9):2917-2924. doi:10.1021/acs.jproteome.8b00505
4. Dellar ER, Vendrell I, Talbot K, et al. Data-independent acquisition proteomics of cerebrospinal fluid implicates endoplasmic reticulum and inflammatory mechanisms in amyotrophic lateral sclerosis. *Journal of Neurochemistry*. 2024/02/01 2024;168(2):115-127. doi:https://doi.org/10.1111/jnc.16030
5. Muntel J, Kirkpatrick J, Bruderer R, et al. Comparison of Protein Quantification in a Complex Background by DIA and TMT Workflows with Fixed Instrument Time. *Journal of Proteome Research*. 2019/03/01 2019;18(3):1340-1351. doi:10.1021/acs.jproteome.8b00898
6. O'Brien DP, Jones HBL, Guenther F, et al. Structural Premise of Selective Deubiquitinase USP30 Inhibition by Small-Molecule Benzosulfonamides. *Molecular & Cellular Proteomics*. 2023;22(8)doi:10.1016/j.mcpro.2023.100609

Supplementary figures and legends

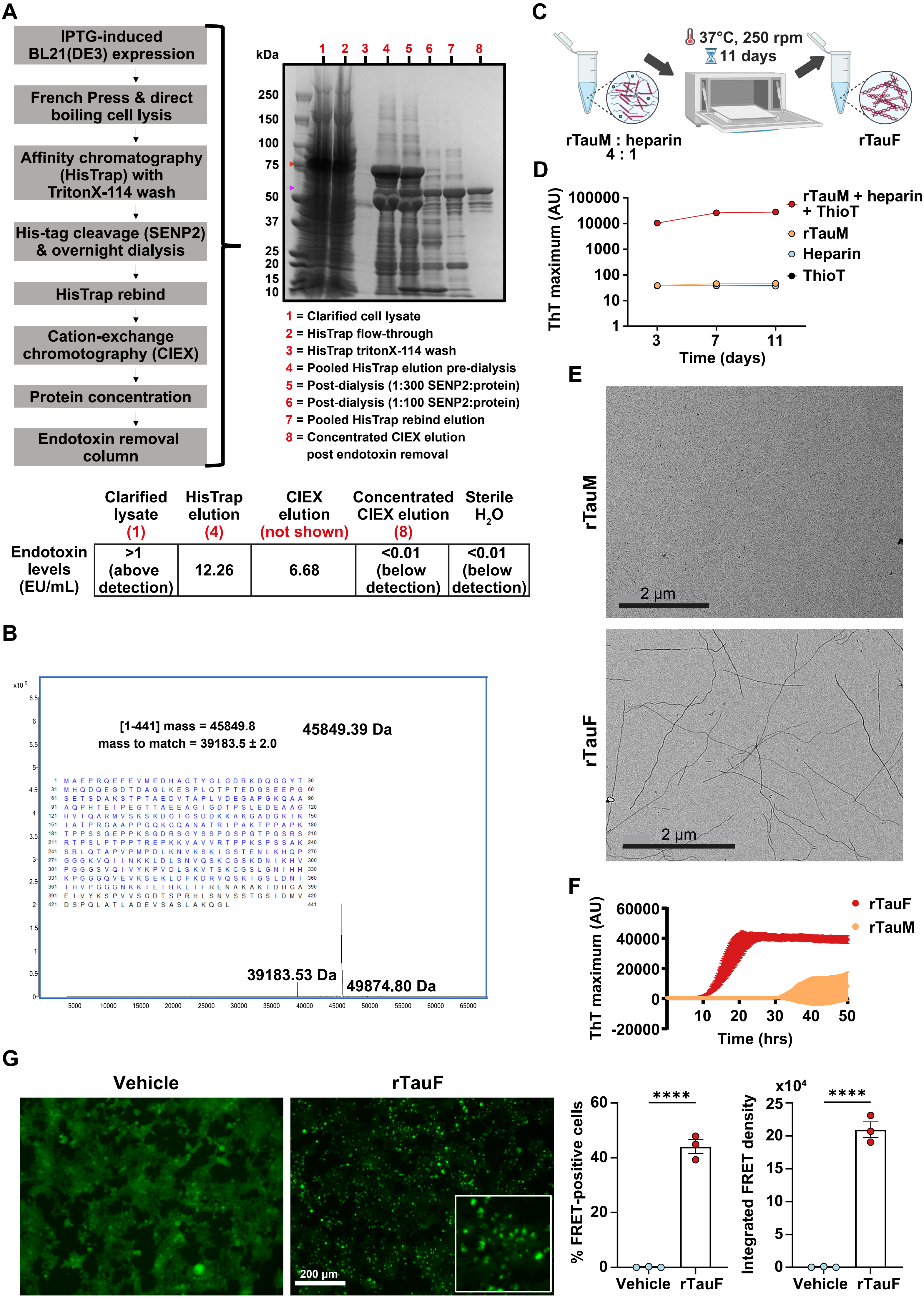

	Clarified lysate (1)	HisTrap elution (4)	CIEX elution (not shown)	Concentrated CIEX elution (8)	Sterile H_2O
Endotoxin levels (EU/mL)	>1 (above detection)	12.26	6.68	<0.01 (below detection)	<0.01 (below detection)

Supplementary Figure 1. Endotoxin-free production of recombinant 2N4R tau.

A Purification workflow and SimplyBlue™-stained gel showing eluted protein fractions. Red arrow points to 6xHis_SUMO3 tagged tau product, the purple arrow points to the tag-less tau product post SENP2 proteolytic cleavage. A 6L bacterial culture yielded a total of 9.1 mg of recombinant tau. Endotoxin levels (EU/mL) in individual purified tau fractions were determined by LAL assay. Numbers in red correspond to fractions shown on the blot. Protein levels were normalised to 1 mg/mL in sterile ddH_2O. **B** Results of intact protein characterisation by LC/MS. **C** Workflow diagram of heparin-induced *in vitro* rTauM aggregation. **D** ThT assay confirming presence of β-sheet structures in rTauF preparation. **E** Negative stain TEM images of rTauM and rTauF. **F** Results of 4R tau RT-QuIC assay investigating seeding of truncated tau substrate (K11, tau aa residues 244-394) in the presence of rTauM and rTauF. A total of 4 technical replicates were run per condition. Data is presented as mean ± SD. **G** Representative images of tau inclusions in FRET Biosensor HEK cells treated with vehicle or transfected with 50 nM rTauF, and quantification of FRET-positive cells and integrated FRET density. Data is represented as mean ± SEM n = 3 technical replicates. Two-tailed unpaired Student t-test was used. ****$P < 0.0001$.

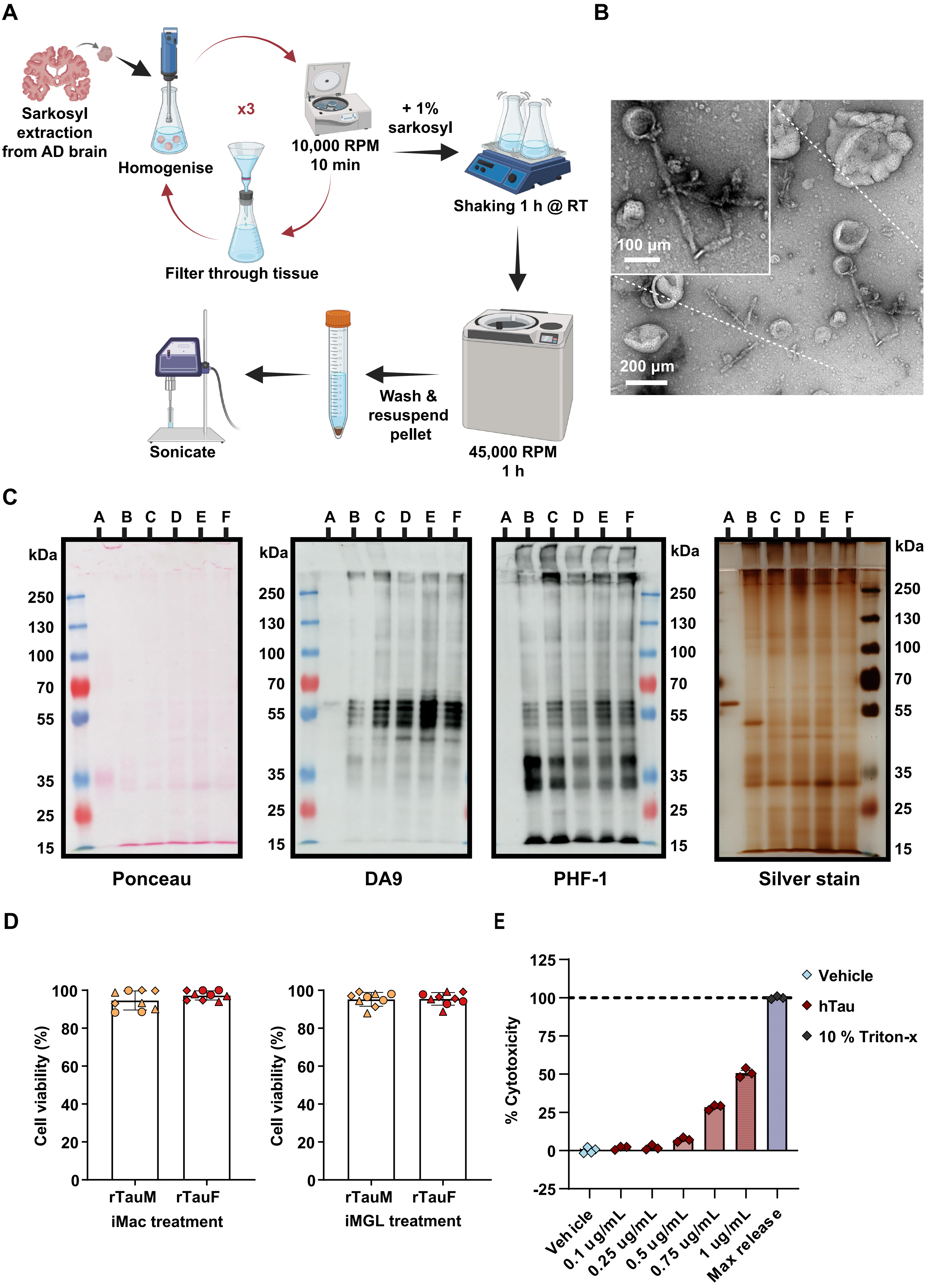
A
Sarkosyl extraction from AD brain
Homogenise
x3
10,000 RPM 10 min
Filter through tissue
+ 1% sarkosyl
Shaking 1 h @ RT
45,000 RPM 1 h
Wash & resuspend pellet
Sonicate
B
100 µm
200 µm
C
A B C D E F
kDa
250
130
100
70
55
35
25
15
Ponceau
DA9
PHF-1
Silver stain
D
Cell viability (%)
rTauM
rTauF
iMac treatment
iMGL treatment
E
% Cytotoxicity
Vehicle
hTau
10 % Triton-x
0.1 ug/mL
0.25 ug/mL
0.5 ug/mL
0.75 ug/mL
1 ug/mL
Max release

Supplementary Figure 2. Purification of hTau from AD brain samples.

A Workflow diagram of sarkosyl-insoluble tau PHFs (hTau) extraction from human AD brain. **B** Negative stain TEM image of extracted hTau preparation with blow-up of a representative fibril with visible helical twists. **C** Quality control western blots for hTau preparations. Markers (left to right): Ponceau (total protein), DA9 (total tau, aa102–140), PHF-1 (pTau S396/S404), silver stain (aggregated tau). Samples (same order for all blots): A: 2N4R/Tau 441 (control), B: Tau seed prep #1, C: Tau seed prep #1 flocculent, D: Tau seed prep #2, E: Tau seed prep #3, F: Pooled tau seed (from preparation B, D and E). **D** Resazurin cell viability assay in iMac/iMGL after 24 h incubation with 4 µg/mL rTauM or rTauF relative to unstimulated control. Data is represented as mean ±SEM of n = 3 in 3 control cell lines. **E** LDH cytotoxicity assay conducted on iMGL supernatants after 24 h incubation with increasing amounts of hTau. Percentage cytotoxicity calculated in relation to the maximum LDH measure from cells treated with 10% Triton-x, vehicle represents untreated iMGL. Data is represented as mean ±SEM of n = 3-4 technical replicates from one control cell line.

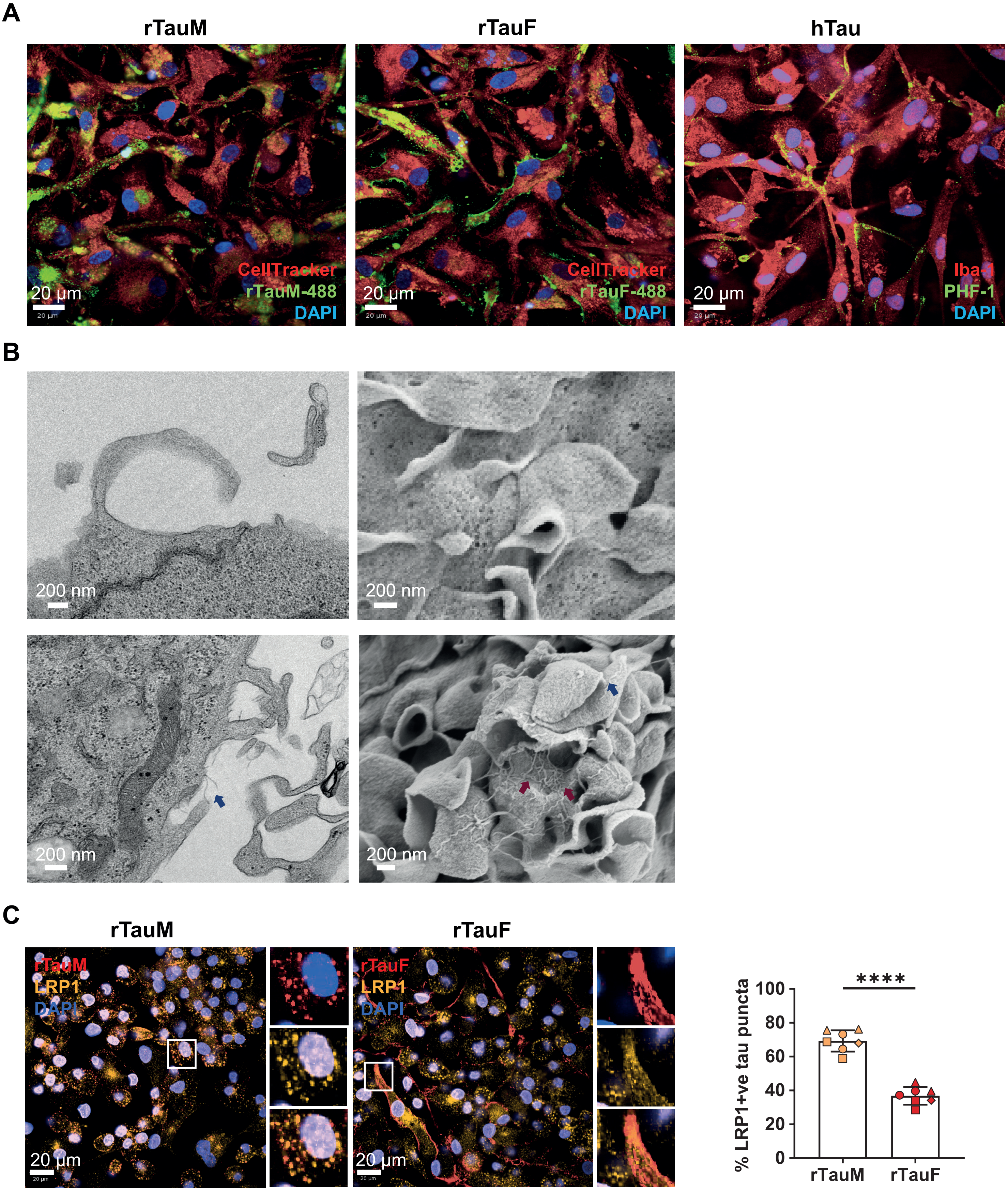

A
rTauM
rTauF
hTau
CellTracker
rTauM-488
DAPI
CellTracker
rTauF-488
DAPI
Iba-1
PHF-1
DAPI
20 µm
B
200 nm
C
rTauM
rTauF
rTauM
LRP1
DAPI
rTauF
LRP1
DAPI
20 µm
% LRP1+ve tau puncta
****
100
80
60
40
20
0
rTauM
rTauF

Supplementary Figure 3. Tau internalisation by iMGL and related iMac

A Representative confocal microscopy images of healthy control-derived iMGL following overnight incubation with DyLight 488-conjugated rTauF, rTauM, or hTau (PHF-1). **B** Transmission (left) and scanning (right) electron micrographs depicting vehicle treated iMac (top) or rTauF tethered to the iMac plasma membrane (bottom, blue arrows) and during uptake (red arrows). Magnification = 69.75 K (iMac) and 40.48 K (iMGL). **C** Representative confocal microscopy images of iMac showing LRP1 colocalising with DyLight 488-conjugated tau monomer and fibrils after 2 h incubation. Quantification of (C) on the right, two-way ANOVA with Šídák multiple comparison test, n = 1-2 in 4 control cell lines.

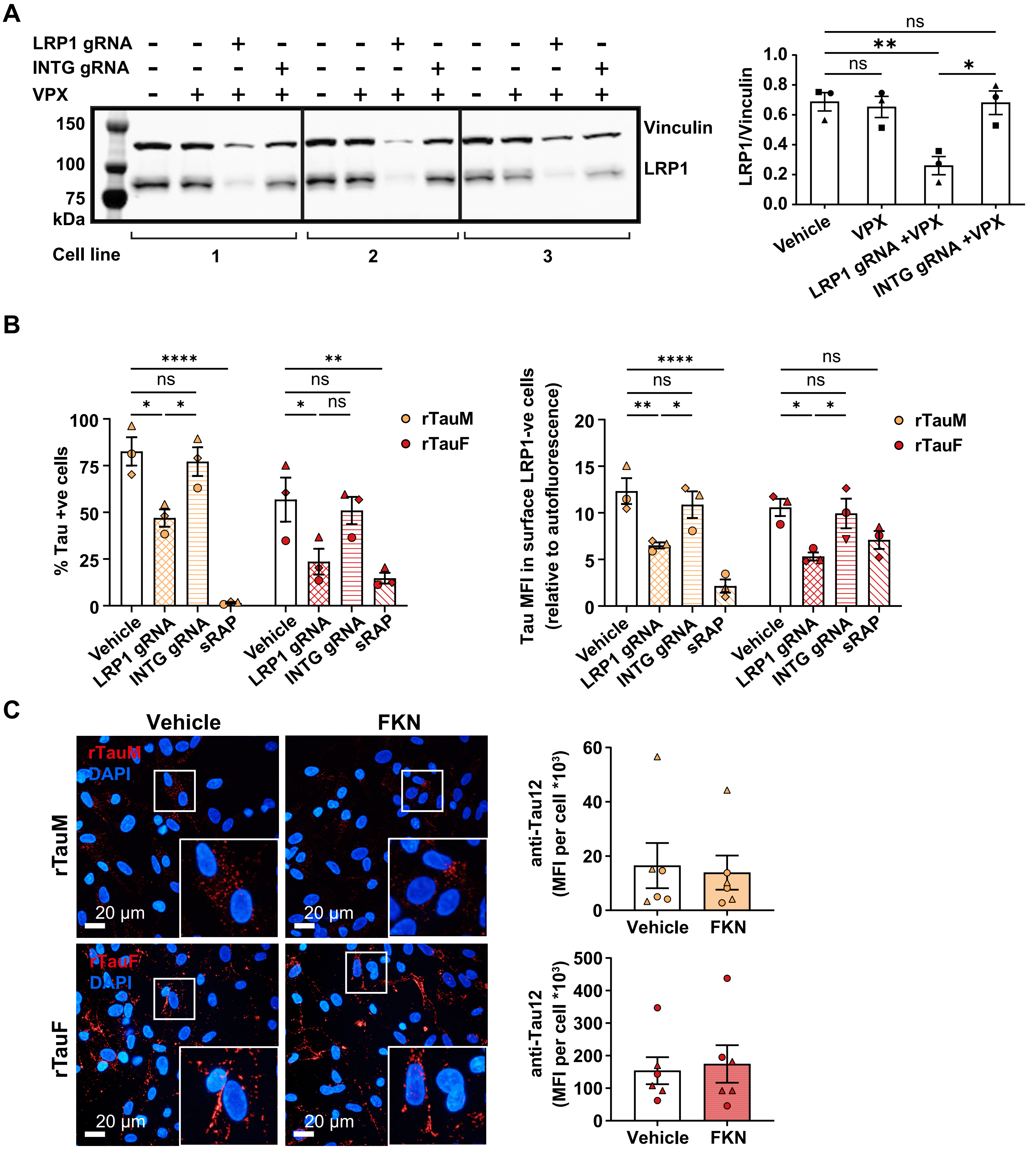
A
LRP1 gRNA
INTG gRNA
VPX
150
100
75
kDa
Vinculin
LRP1
Cell line
1
2
3
LRP1/Vinculin
ns
**
ns
*
Vehicle
VPX
LRP1 gRNA +VPX
INTG gRNA +VPX
B
% Tau +ve cells
****
ns
*
**
rTauM
rTauF
Vehicle
LRP1 gRNA
INTG gRNA
sRAP
Tau MFI in surface LRP1-ve cells (relative to autofluorescence)
C
Vehicle
FKN
rTauM
DAPI
rTauF
20 µm
anti-Tau12 (MFI per cell *10³)

Supplementary Figure 4. Tau entry in iMGL/iMac is LRP1- but not CX3CR1-dependent.

A Western blot confirmation of CRISPR-Cas9 mediated LRP1 KD in iMac. LRP1-targeting versus Intergenic region-targeting (INTG) gRNAs, and transduction enhancer VPX (accessory protein delivered via virus-like particles to facilitate the degradation of SAMHD1, resulting in increasing the availability of dNTPs for viral reverse transcription). Data is displayed as mean ± SEM of n = 1 in 3 control cell lines. One-way ANOVA with Tukey's multiple comparison test was used. **P* < 0.05, ***P* < 0.01. **B** Cas9-LRP1 gRNA lenti-transduction of iMac (versus Intergenic region-targeting gRNA INTG), or blockade with sRAP are associated with a decrease in % of tau+ve cells (left graph) and internalised tau levels in surface LRP1-ve cells (right graph, Mean Fluorescence Intensity relative to autofluorescence). Two-way ANOVA with Tukey's multiple comparisons test, n = 1 in in 3 control cell lines. **C** Representative confocal microscopy images and quantification of Tau12 mean integrated density in iMGL treated with 5 ng/mL FKN for 1 h and challenged with 1 µg/mL rTauM or rTauF for 2 h. Data is represented as mean ± SEM of n = 3 in 2 control cell lines. Two-tailed unpaired Students t-test.

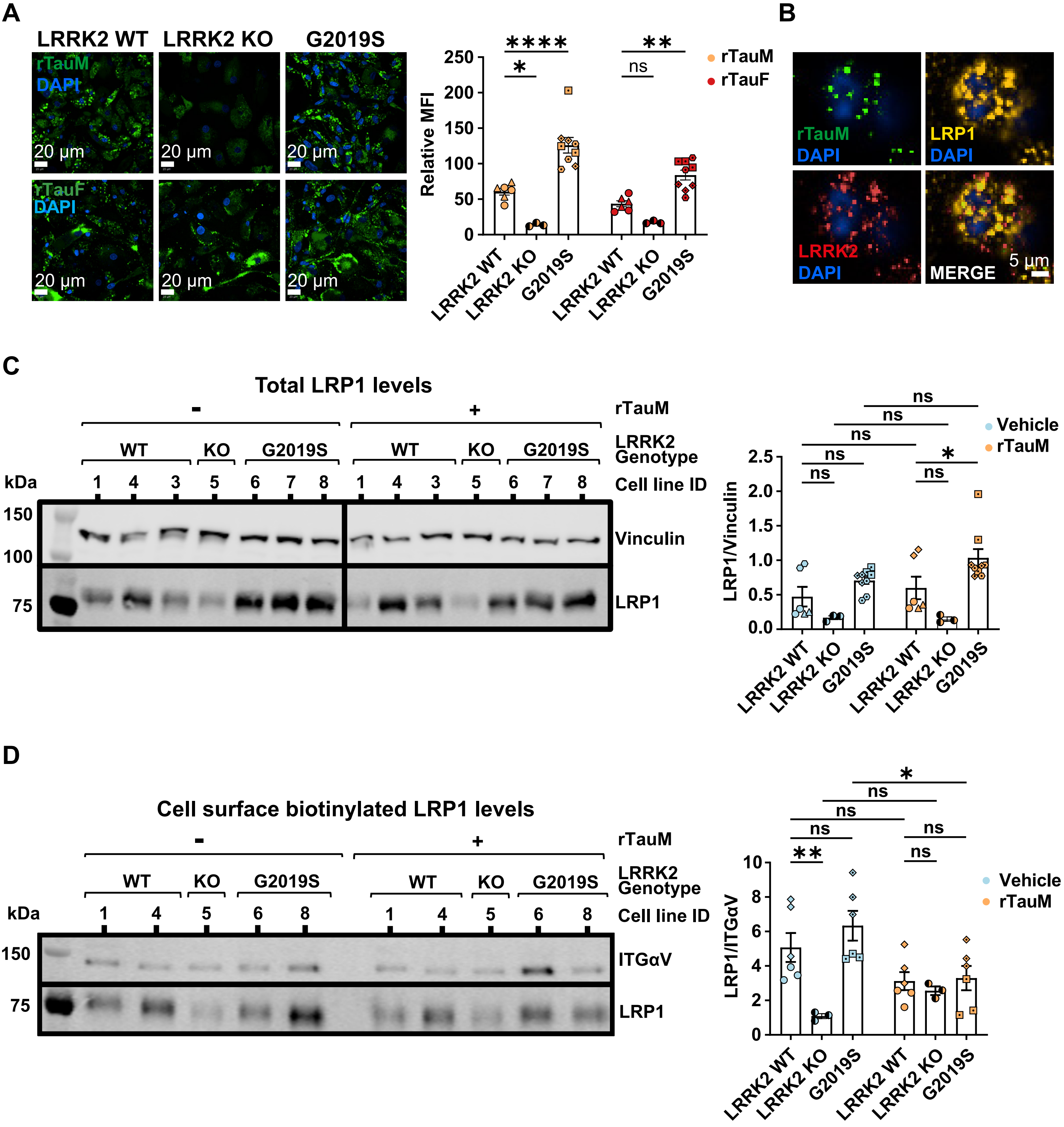
A
LRRK2 WT
LRRK2 KO
G2019S
rTauM
DAPI
rTauF
DAPI
20 µm
Relative MFI
rTauM
rTauF
B
rTauM DAPI
LRP1 DAPI
LRRK2 DAPI
MERGE
5 µm
C
Total LRP1 levels
rTauM
LRRK2 Genotype
Cell line ID
kDa
Vinculin
LRP1
LRP1/Vinculin
Vehicle
rTauM
D
Cell surface biotinylated LRP1 levels
rTauM
LRRK2 Genotype
Cell line ID
kDa
ITGαV
LRP1
LRP1/ITGαV
Vehicle
rTauM

Supplementary Figure 5. LRRK2 genotype influences uptake of DyLight 488-conjugated rTauM and rTauF.

A Representative confocal microscopy images (left) and flow cytometry analysis (right) after 6 h incubation with DyLight 488-conjugated rTauM or rTauF in iMac. MFI of internalised rTauM/rTauF is quantified relative to individual cell-line autofluorescence. Data is shown as mean ± SEM of n = 3 in 2 LRRK2 WT lines, 1 LRRK2 KO line, and 3 G2019S LRRK2 cell lines. Two-way ANOVA with Dunnett's multiple comparisons test *P <0.05, **P < 0.01, ****P < 0.0001. **B** Representative confocal microscopy images showing overlap of LRRK2 puncta with LRP1-rTauM complexes in iMac after 2 h of incubation. **C** Western blot analysis of total and **D** biotinylated surface LRP1 levels in iMac at baseline and after 2 h of rTauM incubation. Data is represented as mean ± SEM of n = 2 in 3 LRRK2 WT, 1 LRRK2 KO, 3 G2019S cell lines for the total LRP1 protein levels, and n = 3 in 2 LRRK2 WT, 1 LRRK2 KO, 2 G2019S cell lines for the surface LRP1 protein levels. Two-way ANOVA with Šídák's multiple comparisons test was used. *P < 0.05, **P < 0.01.

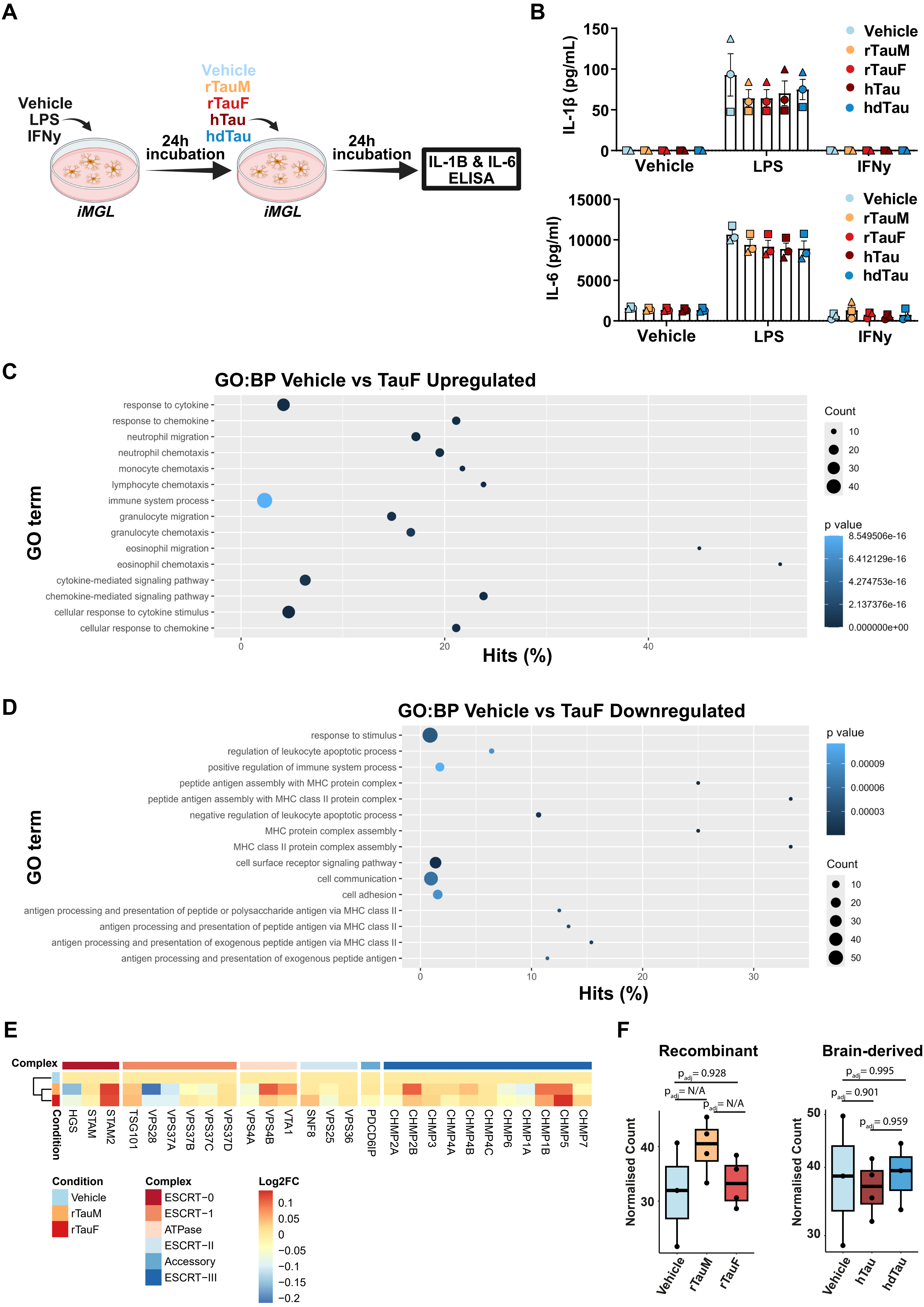
A
Vehicle
LPS
IFNy
iMGL
24h incubation
Vehicle
rTauM
rTauF
hTau
hdTau
iMGL
24h incubation
IL-1B & IL-6 ELISA
B
IL-1β (pg/mL)
Vehicle
LPS
IFNy
IL-6 (pg/ml)
C
GO:BP Vehicle vs TauF Upregulated
response to cytokine
response to chemokine
neutrophil migration
neutrophil chemotaxis
monocyte chemotaxis
lymphocyte chemotaxis
immune system process
granulocyte migration
granulocyte chemotaxis
eosinophil migration
eosinophil chemotaxis
cytokine-mediated signaling pathway
chemokine-mediated signaling pathway
cellular response to cytokine stimulus
cellular response to chemokine
GO term
Hits (%)
Count
p value
D
GO:BP Vehicle vs TauF Downregulated
response to stimulus
regulation of leukocyte apoptotic process
positive regulation of immune system process
peptide antigen assembly with MHC protein complex
peptide antigen assembly with MHC class II protein complex
negative regulation of leukocyte apoptotic process
MHC protein complex assembly
MHC class II protein complex assembly
cell surface receptor signaling pathway
cell communication
cell adhesion
antigen processing and presentation of peptide or polysaccharide antigen via MHC class II
antigen processing and presentation of peptide antigen via MHC class II
antigen processing and presentation of exogenous peptide antigen via MHC class II
antigen processing and presentation of exogenous peptide antigen
GO term
Hits (%)
p value
Count
E
Complex
Condition
HGS STAM STAM2 TSG101 VPS28 VPS37A VPS37B VPS37C VPS37D VPS4A VPS4B VTA1 SNF8 VPS25 VPS36 PDCD6IP CHMP2A CHMP2B CHMP3 CHMP4A CHMP4B CHMP4C CHMP6 CHMP1A CHMP1B CHMP5 CHMP7
Condition: Vehicle, rTauM, rTauF
Complex: ESCRT−0, ESCRT−1, ATPase, ESCRT−II, Accessory, ESCRT−III
Log2FC
F
Recombinant
Brain-derived
Normalised Count
Vehicle rTauM rTauF
Vehicle hTau hdTau

Supplementary Figure 6. Cytokine and Supplementary RNA-seq analyses.

A Schematic of experimental workflow. iMGL were pre-incubated for 24 h with inflammatory stimulants lipopolysaccharide (LPS) or interferon gamma (IFNy) and subsequently treated with rTau or hTau for 24 h, after which supernatants were collected. **B** IL-1β and IL-6 detected in iMGL supernatants by ELISA, n = 1 in 3 control cell lines. **C** Up-regulated Gene Ontology terms for vehicle vs rTauF. **D** Down-regulated Gene Ontology terms for vehicle vs rTauF. **E** Heatmap of Log2FC of the ESCRT pathway genes from DESeq2 comparing Vehicle to rTauM and Vehicle to rTauF. There was only one significant gene expression change, VPS28 being downregulated in rTauM vs Vehicle (Log2FC -0.215, padj=0.003). **F** Normalised read counts for MAPT in iMGL treated with recombinant tau monomer (rTauM) or recombinant tau fibrils (rTauF), human brain-derived tau (hTau), and human brain-derived depleted tau (hdTau). padj values from group comparisons using DESeq2.

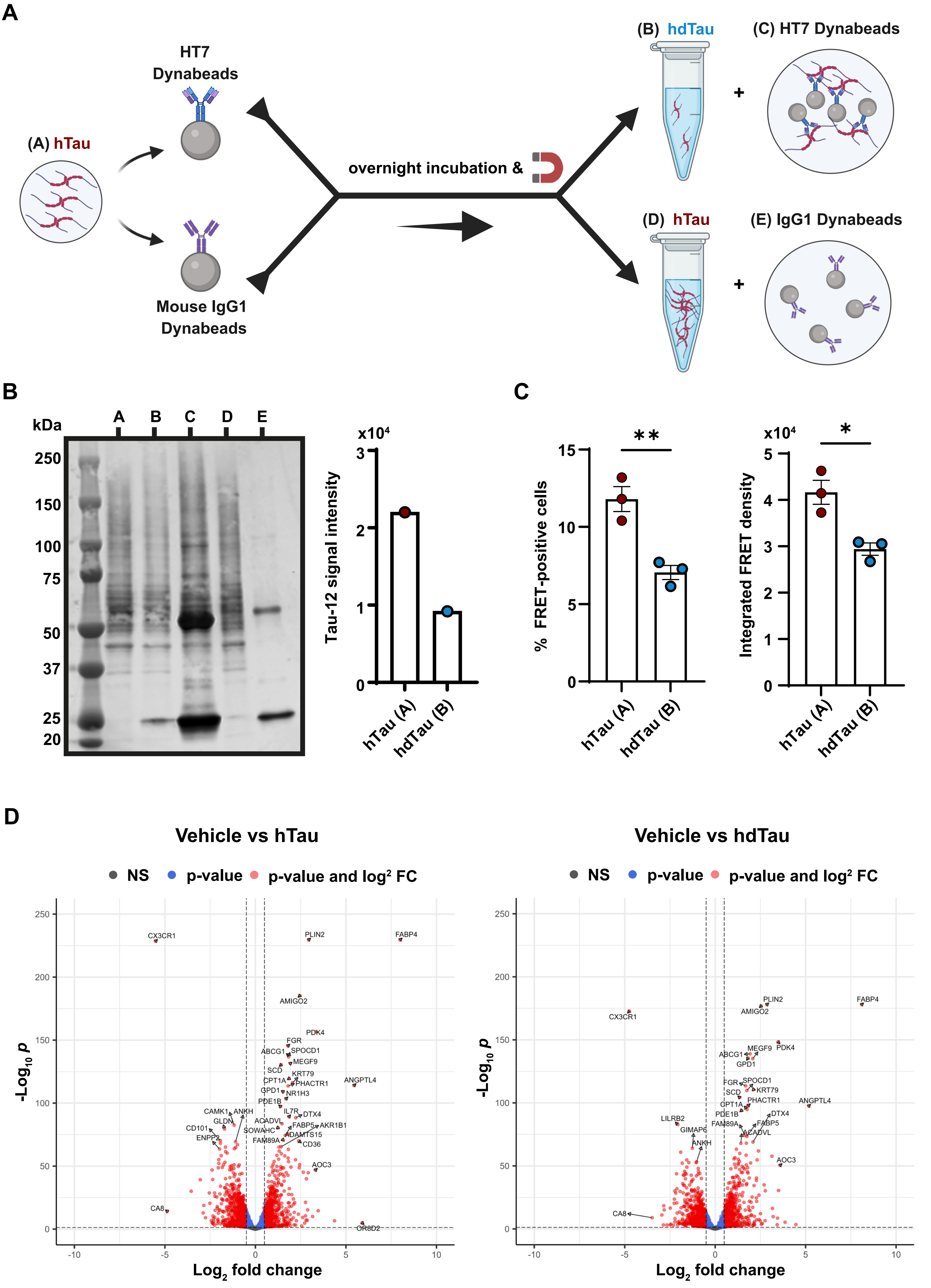
A
(A) hTau
HT7 Dynabeads
Mouse IgG1 Dynabeads
overnight incubation &
(B) hdTau
(C) HT7 Dynabeads
(D) hTau
(E) IgG1 Dynabeads
B
kDa
A B C D E
250
150
100
75
50
37
25
20
x10⁴
Tau-12 signal intensity
hTau (A)
hdTau (B)
C
% FRET-positive cells
Integrated FRET density
**
*
D
Vehicle vs hTau
Vehicle vs hdTau
NS
p-value
p-value and log² FC
-Log₁₀ p
Log₂ fold change
CX3CR1
PLIN2
FABP4
AMIGO2
PDK4
FGR
ABCG1
SPOCD1
MEGF9
SCD
KRT79
CPT1A
PHACTR1
ANGPTL4
GPD1
NR1H3
PDE1B
CAMK1
ANKH
IL7R
DTX4
GLDN
ACADVL
CD101
SOWAHC
FABP5
AKR1B1
ENPP2
ADAMTS15
FAM89A
CD36
AOC3
CA8
OR8D2
LILRB2
GIMAP6

Supplementary Figure 7. hTau immunodepletion.

A Schematic diagram of tau and control IgG immunodepletion from hTau preparation. **B** Western blot confirmation of tau reduction in hdTau preparation (left). Individual lane labels correspond to the schematic labels from (A). Quantification of total tau signal reduction from lanes A and B (right). **C** Percentage of FRET-positive cells and integrated FRET density in Tau RD P301S FRET Biosensor HEK cells transfected with 1 ng of hTau or hdTau. Data is represented as mean ± SEM, n = 3 technical replicates. $^*P < 0.05$, $^{**}P < 0.01$. Two-tailed unpaired Student t-test was used. **D** Volcano plots for vehicle vs hTau (left) and vehicle vs tau-depleted brain sample hdTau (right).

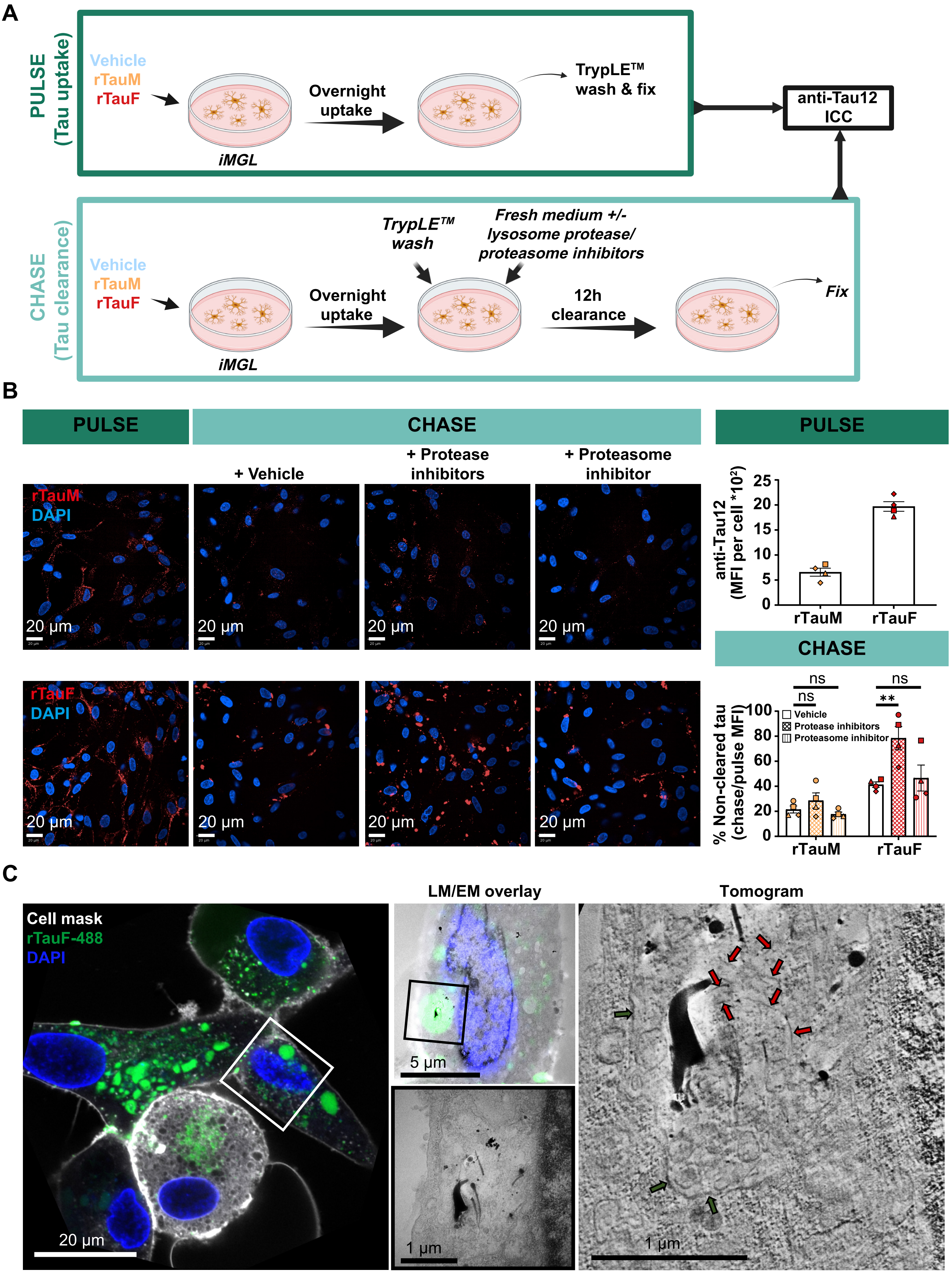
A
PULSE (Tau uptake)
Vehicle
rTauM
rTauF
iMGL
Overnight uptake
TrypLE™ wash & fix
anti-Tau12 ICC
CHASE (Tau clearance)
Vehicle
rTauM
rTauF
iMGL
Overnight uptake
TrypLE™ wash
Fresh medium +/- lysosome protease/ proteasome inhibitors
12h clearance
Fix
B
PULSE
CHASE
+ Vehicle
+ Protease inhibitors
+ Proteasome inhibitor
rTauM
DAPI
rTauF
DAPI
20 µm
PULSE
anti-Tau12 (MFI per cell *10²)
rTauM
rTauF
CHASE
% Non-cleared tau (chase/pulse MFI)
Vehicle
Protease inhibitors
Proteasome inhibitor
ns
ns
ns
**
rTauM
rTauF
C
Cell mask
rTauF-488
DAPI
20 µm
LM/EM overlay
5 µm
1 µm
Tomogram
1 µm

Supplementary Figure 8. Tau degradation pathways and further correlative light and electron microscopy of iMGL treated with Dylight-488-labelled rTauF.

A Treatment schematic. Pulse phase involved only overnight incubation with rTauM/rTauF and enzymatic wash to assess baseline uptake of tau, whereas for the chase conditions, iMGL were incubated with either protease or proteasome inhibitors during the 12 h clearance phase. **B** Representative confocal microscopy images (left) and quantification of Tau12 mean integrated density (right) showing the effect of lysosomal protease inhibition or proteasome inhibition on rTauM and rTauF clearance in iMGL. Data is represented as mean ± SEM of n = 1-2 in 3 control cell lines (pulse) or n = 1 in 4 control cell lines (chase). Two-way ANOVA with Dunnett's multiple comparisons test $^{**}P < 0.01$. **C** Correlative light and electron microscopy (CLEM) images overlay (left) of iMGL incubated overnight (16 h) with Dylight 488-labelled rTauF. Tomogram z-projection (average intensity, right) of the same region containing rTauF (red arrows) within a partial enclosing membrane (green arrows).

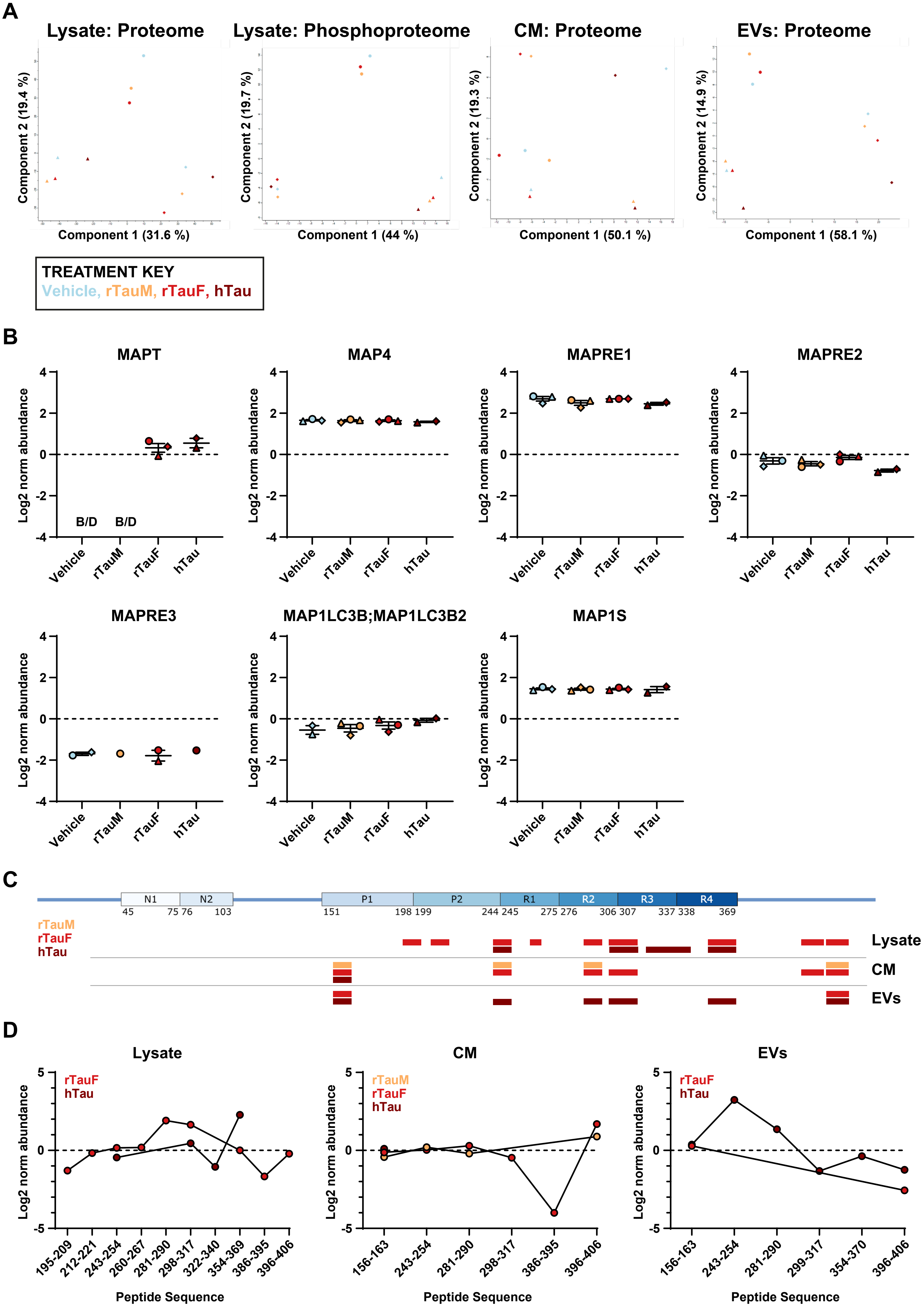

A
Lysate: Proteome
Lysate: Phosphoproteome
CM: Proteome
EVs: Proteome
Component 2 (19.4 %)
Component 1 (31.6 %)
Component 2 (19.7 %)
Component 1 (44 %)
Component 2 (19.3 %)
Component 1 (50.1 %)
Component 2 (14.9 %)
Component 1 (58.1 %)
TREATMENT KEY
Vehicle, rTauM, rTauF, hTau
B
MAPT
MAP4
MAPRE1
MAPRE2
MAPRE3
MAP1LC3B;MAP1LC3B2
MAP1S
Log2 norm abundance
B/D
B/D
Vehicle
rTauM
rTauF
hTau
C
N1
N2
P1
P2
R1
R2
R3
R4
45
75 76
103
151
198 199
244 245
275 276
306 307
337 338
369
rTauM
rTauF
hTau
Lysate
CM
EVs
D
Lysate
CM
EVs
rTauF
hTau
rTauM
Log2 norm abundance
195-209
212-221
243-254
260-267
281-290
298-317
322-340
354-369
386-395
396-406
156-163
299-317
354-370
Peptide Sequence

Supplementary Figure 9. Additional proteomics, MAP family and MAPT peptide mapping.

A Principal Component Analysis plots of all iMGL samples analysed by proteomics (cell lysate, CM and EVs) and phosphoproteomics (cell lysates). **B** Relative abundance of MAPT and other microtubule-associated proteins (MAPs) in lysates of iMGL treated with rTauM, rTauF or hTau for 16 h uptake and 24 h clearance. MAPT peptides in vehicle and rTauM conditions were not identified. Data is represented as mean ± SEM, n = 2-3. B/D: below detection limit. **C** Schematic of 2N4R tau isoform with tau peptides identified by LC-MS/MS. **D** Relative abundance of tau peptides in cell lysates, CM and EVs from iMGL treated with rTauM, rTauF and hTau.

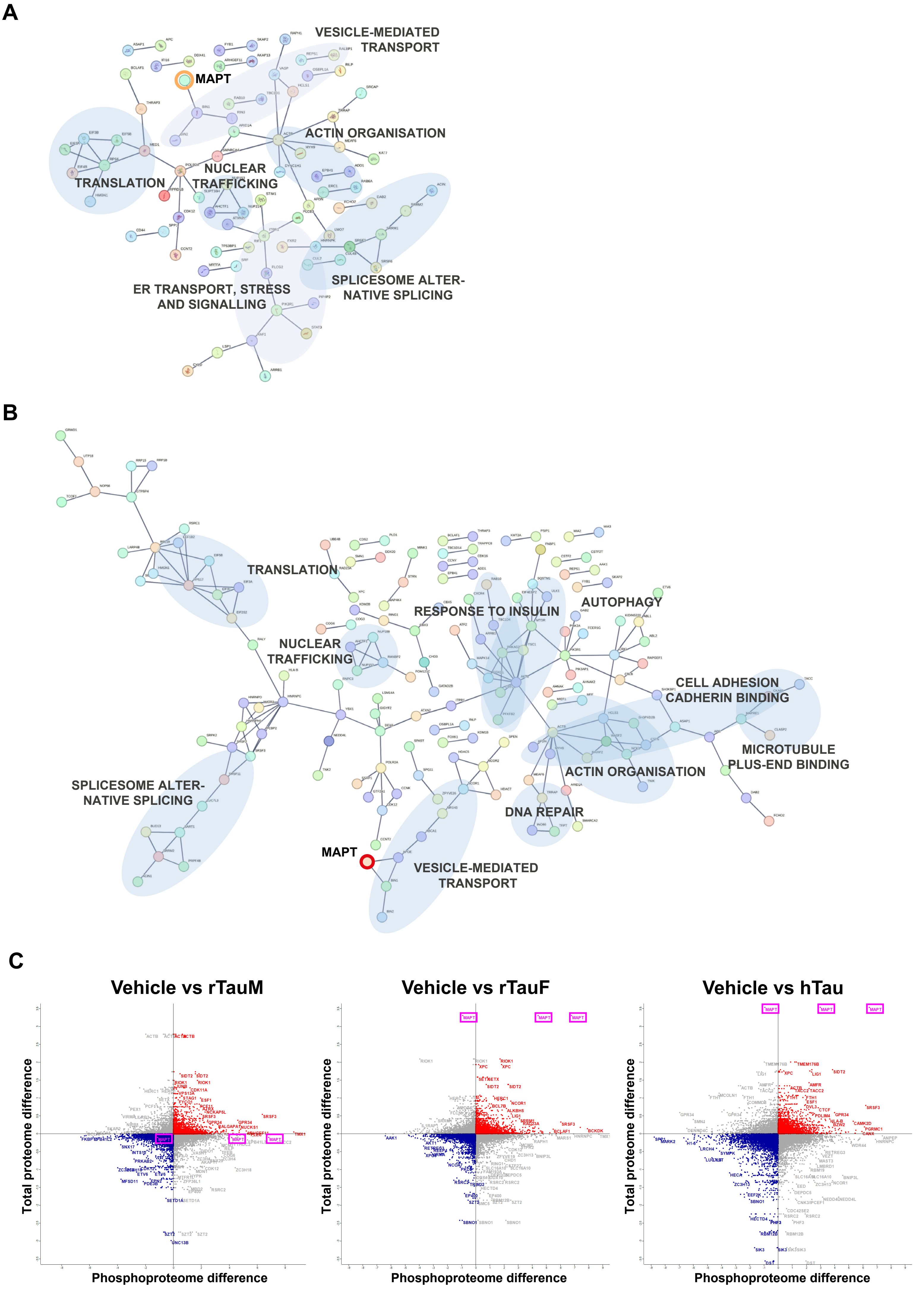
A
VESICLE-MEDIATED TRANSPORT
MAPT
ACTIN ORGANISATION
NUCLEAR TRAFFICKING
TRANSLATION
ER TRANSPORT, STRESS AND SIGNALLING
SPLICESOME ALTER-NATIVE SPLICING
B
TRANSLATION
RESPONSE TO INSULIN
AUTOPHAGY
NUCLEAR TRAFFICKING
CELL ADHESION CADHERIN BINDING
MICROTUBULE PLUS-END BINDING
ACTIN ORGANISATION
SPLICESOME ALTER-NATIVE SPLICING
DNA REPAIR
MAPT
VESICLE-MEDIATED TRANSPORT
C
Vehicle vs rTauM
Vehicle vs rTauF
Vehicle vs hTau
Total proteome difference
Phosphoproteome difference
MAPT

Supplementary Figure 10. Phosphoproteomics of iMGL

A-B STRING analysis of differentially expressed phosphopeptides in iMGL treated with rTauM **(A)** or hTau **(B)**. MAPT highlighted with orange (rTauM, A) or red circle (hTau, B). Only high-confidence interactions (interaction score > 0.9) are displayed. **C** Volcano plots of phosphopeptides versus total proteins identified in iMGL treated with rTauM, rTauF, or hTau compared to vehicle. MAPT peptides highlighted with pink boxes.

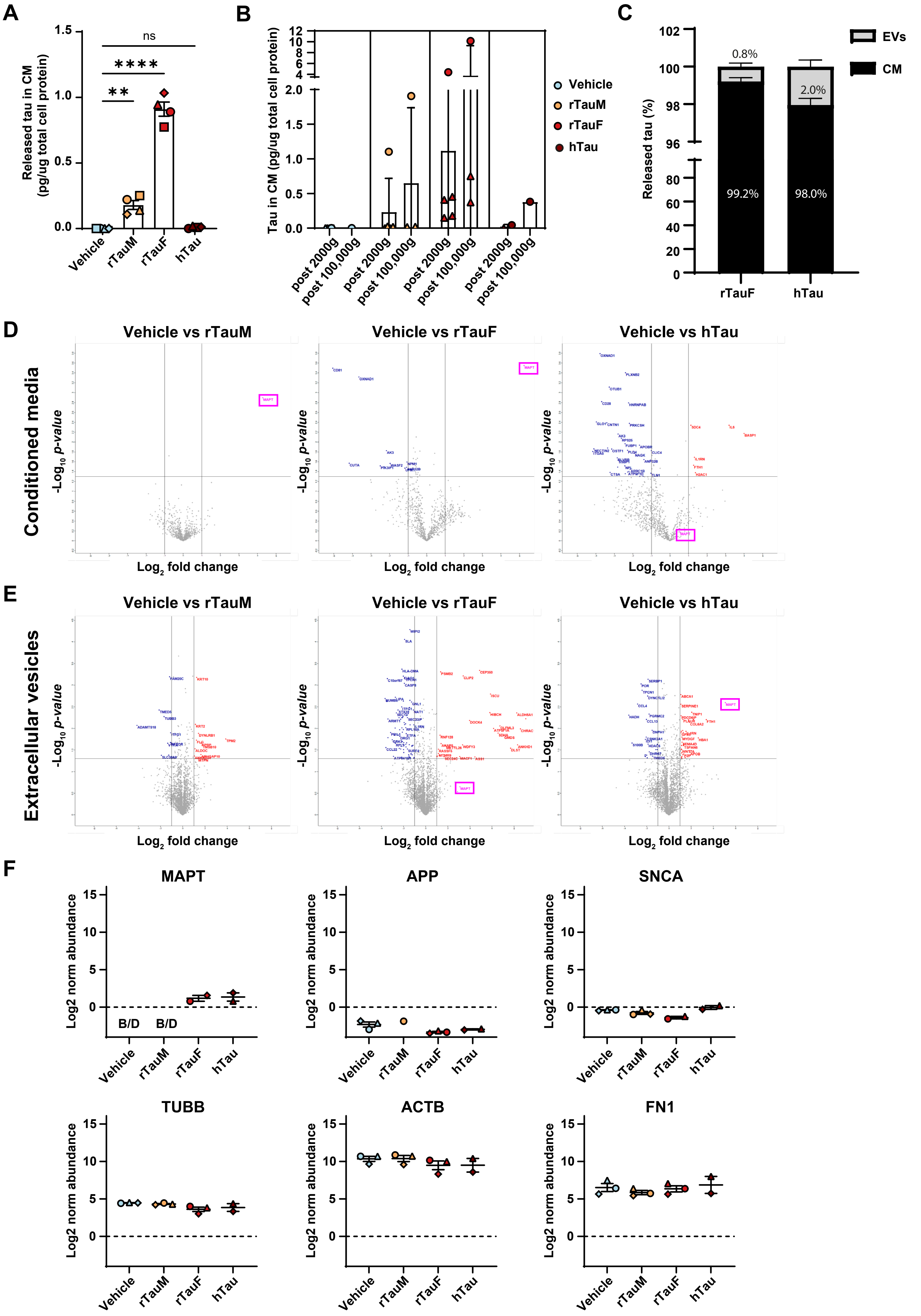
A
Released tau in CM (pg/ug total cell protein)
ns
****
**
Vehicle
rTauM
rTauF
hTau
B
Tau in CM (pg/ug total cell protein)
post 2000g
post 100,000g
Vehicle
rTauM
rTauF
hTau
C
EVs
CM
Released tau (%)
0.8%
2.0%
99.2%
98.0%
rTauF
hTau
D
Conditioned media
Vehicle vs rTauM
Vehicle vs rTauF
Vehicle vs hTau
-Log10 p-value
Log2 fold change
E
Extracellular vesicles
Vehicle vs rTauM
Vehicle vs rTauF
Vehicle vs hTau
-Log10 p-value
Log2 fold change
F
MAPT
APP
SNCA
TUBB
ACTB
FN1
Log2 norm abundance
B/D
B/D
Vehicle
rTauM
rTauF
hTau

Supplementary Figure 11. iMGL secrete tau to the conditioned media and inside extracellular vesicles.

A Tau concentration in the CM of iMGL cells treated with rTauM, rTauF or hTau compared to vehicle, measured by ELISA and normalised to total cell protein. Low levels of detection in the hTau condition could be attributed to lower input amount of tau used for iMGL treatment to avoid toxicity (0.25 ug/mL versus 1 ug/mL used for rTauF) as well as truncated forms of tau not being detected by this ELISA kit. Data is represented as mean ± SEM of n = 1 from 4 control cell lines. One-way ANOVA comparing both rTauM and rTauF to vehicle, with Dunnett's multiple comparisons test. $^{**}P < 0.01$, $^{****}P < 0.0001$ **B** Tau concentration in CM fractions after the 2000 g and the 100,000 g centrifugation steps in the EV purification process, measured by ELISA and normalised to total cell protein. Data is represented as mean ± SEM of n = 1-5 from 2 control cell lines. **C** The percentage of total released tau associated with EVs and the CM, measured from the same samples, n = 1 from 2-3 control cell lines . **D** Differentially expressed proteins in the CM of iMGL cells challenged with rTauM, rTauF or hTau. MAPT marked with pink boxes. Vertical line: fold change above 2; Horizontal line: significant proteins (-logp >1.301 or p<0.05). **E** Differentially expressed proteins in isolated EVs from iMGL challenged with rTauM, rTauF or hTau. **F** Relative abundance of MAPT, amyloid precursor protein (APP), α-synuclein (SNCA), tubulin (TUBB), actin (ACTB) and fibronectin (FN1) in isolated EVs from iMGL treated with rTauM, rTauF or hTau. Data is represented as mean ± SEM, n = 2-3. B/D: below detection limit.

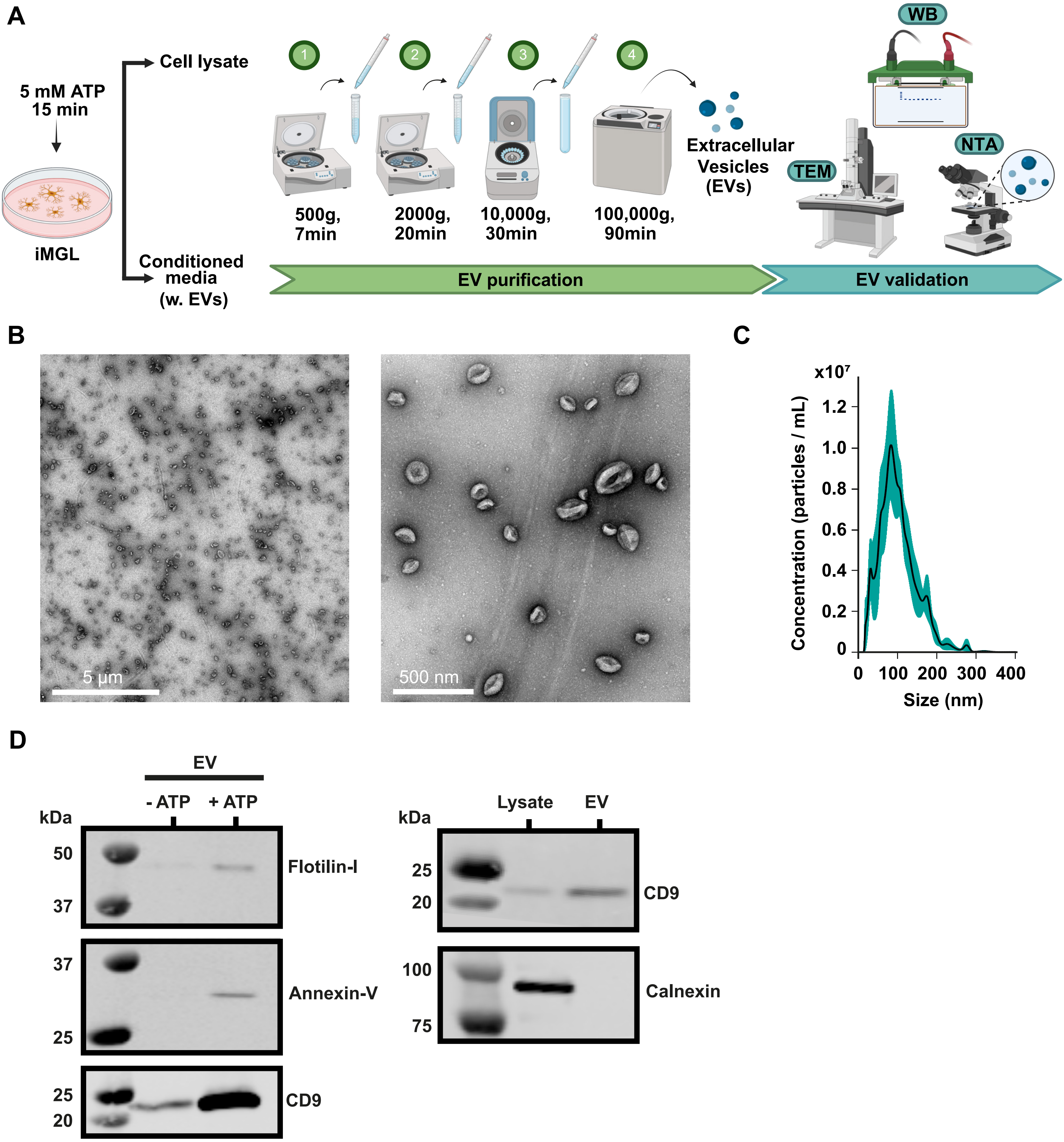
A
5 mM ATP
15 min
iMGL
Cell lysate
Conditioned media (w. EVs)
1
2
3
4
500g, 7min
2000g, 20min
10,000g, 30min
100,000g, 90min
Extracellular Vesicles (EVs)
WB
TEM
NTA
EV purification
EV validation
B
5 µm
500 nm
C
x10⁷
Concentration (particles / mL)
1.2
1.0
0.8
0.6
0.4
0.2
0
0
100
200
300
400
Size (nm)
D
EV
- ATP
+ ATP
kDa
50
37
Flotilin-I
37
25
Annexin-V
25
20
CD9
Lysate
EV
kDa
25
20
CD9
100
75
Calnexin

Supplementary Figure 12. Validation of the EV purification process.

A Experimental workflow illustrating the purification of EVs from iMGL cells treated with 15 mM ATP (15 min) by a series of centrifugation steps of increasing speed and validated with both transmission electron microscopy (TEM), nanoparticle tracking analysis (NTA) and western blotting (WB). **B** Representative TEM images at different magnifications illustrating shape and sizes of purified EVs. **C** Quantification of particle sizes via NTA. **D** Enriched expression of EV markers Flotilin-I, Annexin-V and CD9 as a result of ATP stimulation (left). Validation of EVs with positive control marker CD9 and negative control marker calnexin, only expressed in cell lysate (right).

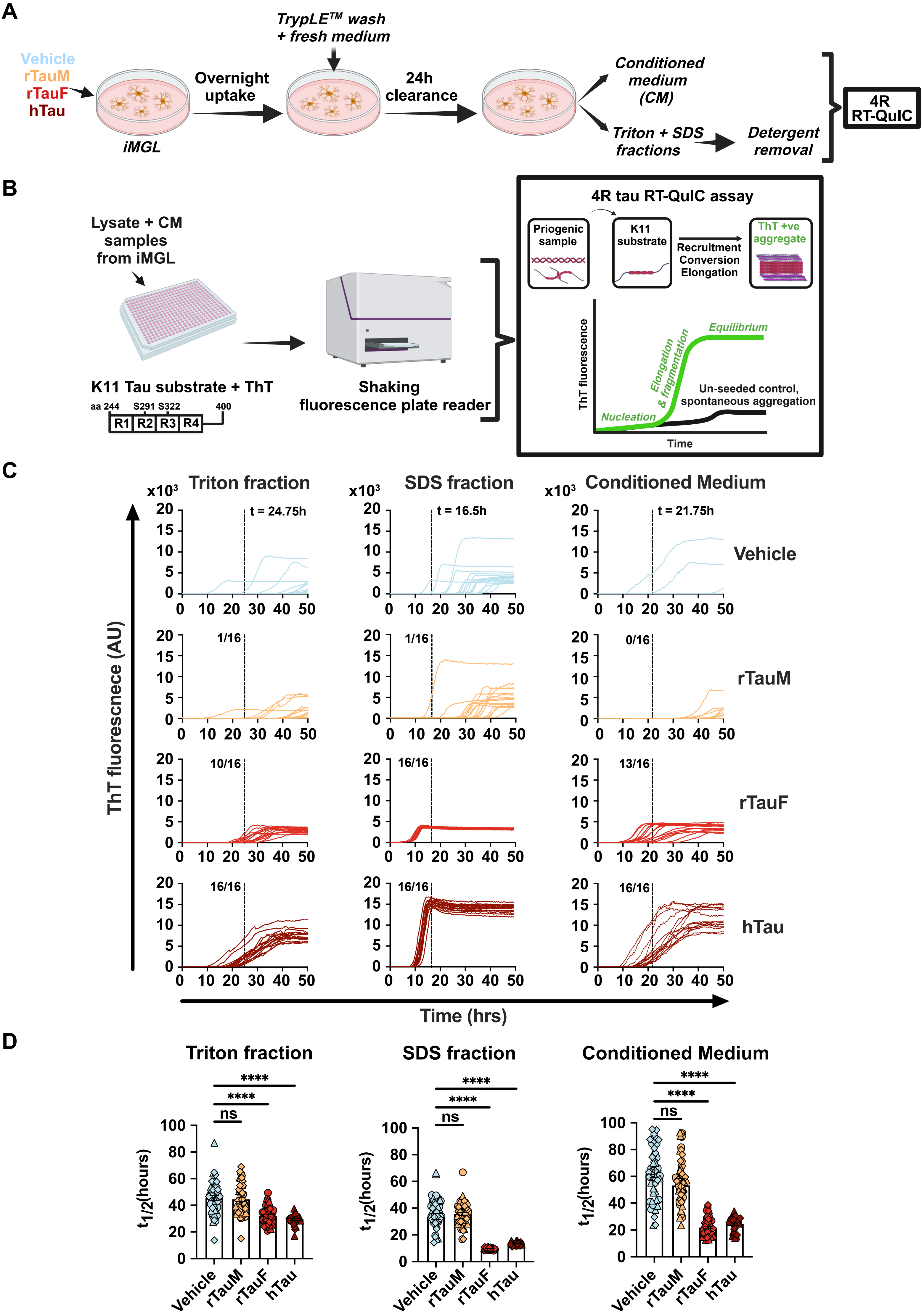
A
Vehicle
rTauM
rTauF
hTau
iMGL
Overnight uptake
TrypLE™ wash + fresh medium
24h clearance
Conditioned medium (CM)
Triton + SDS fractions
Detergent removal
4R RT-QuIC
B
Lysate + CM samples from iMGL
K11 Tau substrate + ThT
aa 244
S291 S322
400
R1 R2 R3 R4
Shaking fluorescence plate reader
4R tau RT-QuIC assay
Priogenic sample
K11 substrate
Recruitment Conversion Elongation
ThT +ve aggregate
ThT fluorescence
Time
Nucleation
Elongation & fragmentation
Equilibrium
Un-seeded control, spontaneous aggregation
C
Triton fraction
SDS fraction
Conditioned Medium
x10³
t = 24.75h
t = 16.5h
t = 21.75h
Vehicle
rTauM
rTauF
hTau
1/16
1/16
0/16
10/16
16/16
13/16
16/16
16/16
16/16
ThT fluorescnece (AU)
Time (hrs)
D
Triton fraction
SDS fraction
Conditioned Medium
ns
****
****
t1/2(hours)
Vehicle
rTauM
rTauF
hTau

Supplementary Figure 13. Seeding capacity of tau taken up by iMGL, assessed with RT-QuIC.

A Experimental workflow. **B** 4R tau RT-QuIC workflow and illustration of expected curves from the assay and their interpretation. **C** Representative seeding activity of samples collected from a control iMGL cell line. Total protein levels in cell lysate fractions and media were normalised to 1 mg/mL. Triton and SDS lysate fractions were applied to the reaction buffer at 1:10,000 dilution, conditioned media at 1:100, to account for tau concentration differences. Dashed line is set at a kinetic cut-off between templated substrate seeding vs spontaneous aggregation. A reaction was considered seeded upon reaching ThT fluorescence threshold > 100 * SD baseline fluorescence[1-4]. Numbers refer to the fraction of the 16 replicates for a given sample type that reaches the kinetic cut-off. **D** t1/2 calculated from raw RT-QuIC ThT reactions as sigmoidal dose-response, variable slope logEC50. Data is represented as mean ± SEM of n = 1 in 3 control cell lines and 16 technical replicates per sample. Two-way ANOVA with Tukey's multiple comparisons test. $^{*}P < 0.05$, $^{**}P < 0.01$, $^{****}P < 0.0001$.
